# Origin and characterization of cyclodepsipeptides: Comprehensive structural approaches with focus on mass spectrometry analysis of alkali‐cationized molecular species

**DOI:** 10.1002/mas.21904

**Published:** 2024-08-21

**Authors:** Sophie Liuu, Annelaure Damont, Alain Perret, Olivier Firmesse, François Becher, Gwenaëlle Lavison‐Bompard, Amandine Hueber, Amina S. Woods, Ekaterina Darii, François Fenaille, Jean‐Claude Tabet

**Affiliations:** ^1^ Staphylococcus, Bacillus & Clostridium (SBCL) unit, Laboratory for Food Safety French Agency for Food, Environmental and Occupational Health & Safety (ANSES), Université Paris‐Est Maisons‐Alfort France; ^2^ Université Paris‐Saclay, CEA‐INRAE, Laboratoire Innovations en Spectrométrie de Masse pour la Santé (LI‐MS), DRF/Institut Joliot/DMTS/SPI, MetaboHUB CEA Saclay Gif sur Yvette France; ^3^ Génomique métabolique, Genoscope Institut François Jacob, CEA, CNRS, Univ Evry, Université Paris‐Saclay Evry France; ^4^ Pesticides and Marine Biotoxins (PBM) unit, Laboratory for Food Safety French Agency for Food, Environmental and Occupational Health & Safety (ANSES), Université Paris‐Est Maisons‐Alfort France; ^5^ National Institute on Drug Abuse Intramural Research Program (NIDA IRP) National Institute of Health (NIH) Baltimore Maryland USA; ^6^ Johns Hopkins School of Medicine Pharmacology and Molecular Sciences Baltimore Maryland USA; ^7^ Faculté des Sciences et de l'Ingénierie, Institut Parisien de Chimie Moléculaire (IPCM) Sorbonne Université Paris France

**Keywords:** alkali‐cationized molecule, cyclodepsipeptide, mass spectrometry, MS/MS, structure

## Abstract

Cyclodepsipeptides (CDPs) represent a huge family of chemically and structurally diverse molecules with a wide ability for molecular interactions. CDPs are cyclic peptide‐related natural products made up of both proteinogenic and nonproteinogenic amino acids linked by amide and ester bonds. The combined use of different analytical methods is required to accurately determine their integral structures including stereochemistry, thus allowing deeper insights into their often‐intriguing bioactivities and their possible usefulness. Our goal is to present the various methods developed to accurately characterize CDPs. Presently, Marfey's method and NMR (nuclear magnetic resonance) are still considered the best for characterizing CDP configuration. Nevertheless, electrospray‐high resolution tandem mass spectrometry (ESI‐HRMS/MS) is of great value for efficiently resolving CDP's composition and sequences. For instance, recent data shows that the fragmentation of cationized CDPs (e.g., [M + Li]^+^ and [M + Na]^+^) leads to selective cleavage of ester bonds and specific cationized product ions (**b** series) useful to get unprecedented sequence information. Thus, after a brief presentation of their structure, biological functions, and biosynthesis, we also provide a historic overview of these various analytical approaches as well as their advantages and limitations with a special emphasis on the emergence of methods based on HRMS/MS through recent fundamental works and applications.

**Abbreviations**
AA: amino acid
APCI: atmospheric pressure chemical ionization
API: atmospheric pressure ionization
BEQ: magnetic, electric and quadrupole instrument
CBC: covalent bond cleavage
CD: circular dichroism
CDP: cyclodepsipeptide
CI: chemical ionization
CID: collision induced dissociation
CS: cation solvated or charge solvated
DAD: diode array detector
DART: direct analysis in real time
DC: direct current
DCI: desorption chemical ionization
DESI: desorption electrospray ionization
DHOYA: 2,2‐dimethyl‐3‐hydroxy‐7‐octynoic acid
EBE: Electric, magnetic and electric triple sector instrument
EBEB: Electric, magnetic, electric and magnetic four sector instrument
ECD: electron capture dissociation
EE: even electron
EI: electron ionization
EID: electron induced dissociation
ERMS: energy resolved mass spectrometry
ESI: electrospray ionization
ETD: electron transfer dissociation
EV: electron volt
FAB: fast atom bombardment
FD: field desorption
FFR: field‐free region
FT: Fourier transform
GC: gas chromatography
GC‐EIMS: gas chromatography electron ionization mass spectrometry
HA: hydroxy acid
HAA: hydroxy amino acid
HCD: higher collision energy
HFA: hydroxy fatty acid
HMBA: 2‐hydroxy‐3‐methylbutyric acid
HMOA: ß‐hydroxy‐4‐methyl octanoic acid
HMOAA: (3 S,2 R)‐3‐hydroxy‐2‐methyloct‐7‐anoic acid
HMOEA: (3 S,2 R)‐3‐hydroxy‐2‐methyloct‐7‐enoic acid
HMOYA: (3 S,2 R)‐3‐hydroxy‐2‐methyloct‐7‐ynoic acid
HMPA: 2‐hydroxy‐3‐methyl‐pentanoic acid
HPLC: high performance liquid chromatography
HRMS: high resolution mass spectrometry
ICR: ion cyclotron resonance
IDA: isotope dilution assay
IR: infrared
IRLD: infrared laser desorption
IRMPD: infrared multiphoton dissociation
KE: kinetic energy
LC: liquid chromatography
LD: laser desorption
LIT: linear ion trap
LLE: liquid‐liquid extraction
LMCO: low‐mass cut‐off
LOD: limit of detection
M/Z: mass over charge
MAD: metastable atom‐activated dissociations
MALDI: matrix assisted laser desorption ionization
MIKE: mass‐analyzed‐ion kinetic energy
MRM: multiple reaction monitoring
MS/MS: tandem mass spectrometry
MS: mass spectrometry
NAC: N‐acyl
NME: N‐methyl
NMR: nuclear magnetic resonance
NRPS: non‐ribosomal peptides synthesases
OE: odd‐electron
OTMSi: O trimethylsilyl
PCPs: peptidyl carrier proteins
PIC: precursor ion current
PLA: phenyl lactic acid
PS: protonated salt
QUECHERS: quick, easy, cheap, effective, rugged, and safe
RF: radio‐frequency
RP: reverse phase
SAA: Sugar Amino Acids or sugar amino alcohols
SID: surface induced dissociation
SIDA: stable isotope dilution analysis
SIMS: secondary ion mass spectrometry
SPE: solid phase extraction
TIC: total ion current
TOF: time of flight
TPIC*: total product ion current without alkali metal ion abundance
TPIC: total product ion current
UV: ultra‐violet

## 1 INTRODUCTION

Most pharmaceuticals are based on natural product molecules from living organisms (Li & Vederas, 2009) and are derived from secondary metabolism. Unlike primary (or central) metabolism, which is assumed to be conserved in all organisms and generates compounds essential for cellular homeostasis, secondary metabolism uses primary metabolites to form molecules of adaptation that evolved for purposes other than primary metabolism. In contrast to primary metabolites, they are synthesized by individual species or genera for specific physiological, social or predatory purposes. Among this immense chemical diversity, cyclodepsipeptides (CDPs) form a huge family of compounds of great interest both for their therapeutic potential and their structural diversity (Taevernier et al., 2017). Thus, since the 1960s, much work was undertaken for their structural elucidation.

One of the most precise and accurate analytical method for obtaining accurate chemical characterization of CDPs (including distinction of isomeric, stereoisomeric structures, and conformation) is nuclear magnetic resonance (NMR). NMR‐based approaches have been successfully used for characterizing such cyclic molecular systems, sometimes bearing an exocyclic peptide or fatty acid alkyl side chain. However, this approach is often intrinsically limited by its detection sensitivity, as it may require several tens of micrograms of pure sample to yield informative spectra. Unfortunately, such pure sample amounts can be difficult to obtain from most naturally occurring CDPs which are present in trace amounts in complex mixtures. On the other hand, the popular Edman sequencing method (Edman, 1949; Gomes, 2022) is irrelevant because CDPs are cyclic systems without an N‐terminus accessible to the formation of phenyl iso‐thiocyanide (PITC) derivatives.

In general, mass spectrometry (MS)‐based structural analytical methods for CDPs (and peptides) have been influenced by the progressive evolution of this technique over the last six decades. Initially, the only MS ionization technique used was the gas‐phase electron ionization (EI). It occurs as a very rapid step (~10^−19^ s) yielding the molecular ion M^+·^ (with an odd‐electron number, OE). Then, M^+·^ species undergo prompt fragmentation (albeit slower, reaching less than the 10^−6 ^s) in the ion source, sometimes leading to its complete disappearance. In fact, the transition into the gas phase of CDPs as intact ionized species was the first challenge. The second difficulty was to rationalize the dissociation processes of the M^+·^ ion, which acquired a high internal energy (~up 15 eV) during ionization due to “vertical ionization” (Franck–Condon principle based on vibronic transitions). This internal energy consists of a vibrational energy distribution in both the ground and excited electronic states, which is impossible to control with commercial instruments. Thus, chemical interpretation of mass spectra is sometimes difficult, given the complexity of possible mechanisms depending on the origin of this internal energy, where the chemistry of radicals can be confounded with that of cations with an even‐electron number (EE). The much lower energetic chemical ionization (CI), based on the gas phase ion‐molecule reactions (mainly involving proton exchange with ionized reactive gases), leads to positive and negative EE ions. They are more stable than OE molecular ions and their dissociations are promoted by the charge. The first major MS advances were made in desorption methods in the mid 1970s, initially with field desorption (FD), then bombardment either by particles such as fast fragments from ^252^Cf decay (plasma desorption MS), ions (secondary‐ion MS, SIMS), and atoms (fast atom bombardment MS, FABMS), or by radiation (laser desorption MS, LDMS). In the middle of the next decade, matrix‐assisted laser desorption/ionization (MALDI, with spontaneous aggregate desolvation) in vacuum and electrospray desorption/ionization (ESI) in atmospheric pressure (AP) conditions, emerged as modern approaches. They provide access to stable charged molecular species where the main part of internal energy is transferred during desolvation of desorbed ionic aggregates. This energy is better controlled in ESI, so that only (or not) molecular species can be obtained within a protomer (or deprotomer) distribution depending on the solvent and desolvation conditions. This mode of desorption/ionization, coupled with various forms of liquid chromatography (LC‐MS), is nowadays the most widely used for CDP analysis. Meanwhile, in the 1970s, tandem mass spectrometry (MS/MS) was introduced using multisector instruments, enabling the structural analysis of ions through their specific selection and their subsequent dissociations, metastable or collision‐induced, at several keV energies. At the end of this decade, by using triple quadrupole instruments, dissociations were provided by low‐energy collisional processes (i.e., collision‐induced dissociation, CID). For instance, dissociation of protonated linear peptides (with n residues) leads to product ion series with charge retention at either the N‐terminal side (primary amine) or the C‐terminal side (carboxylic acid), respectively such as the **b**_***i***_ series (and/or the **a**_*i*_ series) and the **y*_n_***_***‐i***_ series, according to the Biemann/Roepstorff nomenclatures (Biemann, 1988; Roepstorff & Fohlman, 1984). The orientation in favor of one or the other ion series (or reinforcement of a particular product ion) depends on the functional groups on the side chain of the residues and the N‐terminus. In the case of protonated cyclopeptides with n residues, the ring‐opening promoted by the proton occurring at each amide bond results in a mixture of peptide **b**_***n***_ isomeric fragment ions, that is, with various N‐terminus and various C‐terminus with an acylium end group (intact or rearranged into protonated oxazolone; Paizs & Suhai, 2005). Consequently, the collisional spectrum must display, at least, a set of product ions b_i_ characterizing each sequence of the isomeric open cyclopeptide **b**_***n***_ precursor ions. Despite an overlapping of different sequences, it is possible to obtain the sequence without any difficulty.

The behavior of protonated CDPs towards collisional activation is drastically more complicated than that of linear peptides of similar length, as in some cases ring‐opening may occur at the ester or amide bonds in a rather random fashion, thus yielding a complex set of “opened peptides” with varying termini. To date, the mechanisms involved in these seemingly random dissociative processes have not been fully rationalized. The real challenge lies in understanding the dissociation of ions in the gas phase, which depends on variability in the chemical reactivity of solvent‐free ions. An ideal alternative would be to find an “in situ” derivatization method that would facilitate the interpretation of collisional spectra. For such purpose, a specific ring‐opening would be very useful, especially if followed by a privileged orientation of the decomposition of the open forms to give the product ions with retention of the derivatization tag as an initial labelling of the terminal group.

High‐ and ultra high‐resolution mass analyzers such as time of flight (ToF) and Fourier transform analyzers (i.e., FT ion cyclotron resonance, FTICR, and Orbitrap) are routinely used for many biological applications including CDPs analysis. This non‐exhaustive review is dedicated to the characterization of CDPs and the analytical methods used to achieve it. Thus, after a presentation of their structures and their biological functions and biosynthesis, we will briefly present the NMR methods initially developed along with their advantages and limitations, both used alone or in combination with MS. Then this review will detail the various MS‐based methods^1^ that have been developed since the early 1960s to the most recent developments. We will specifically emphasize how the latest methodological and experimental refinements using high‐resolution tandem mass spectrometry (HRMS/MS) can give access to CDP's proteinogenic and non‐proteinogenic amino acid residue composition and sequence. We will also illustrate some applications of MS‐based methods in the food safety and pharmaceutical sectors.

## 2 STRUCTURE, FUNCTIONS, AND BIOSYNTHESIS OF CDPs

### 2.1 Structure

CDPs constitute a large family of cyclic peptide‐related products in which at least one of the amide groups is replaced by the corresponding ester (International Union of Pure and Applied Chemistry [IUPAC], 2006). A consequence of replacing the NH group in the amide bond with an oxygen atom is a more flexible structure for depsipeptides than their native analogues (Avan et al., 2014). CDPs therefore have complex and diverse structures that vary according to the nature and order of the amino and hydroxy acid residues that make up their ring (Avan et al., 2014; Taevernier et al., 2017). CDPs are not only composed of proteinogenic amino acids but also of many other building blocks, including d‐amino acids, non‐proteinogenic amino acids, N‐terminally attached fatty acid chains, N‐ and C‐methylated residues, N‐formylated residues, heterocyclic elements, and glycosylated amino acids, as well as phosphorylated residues (Grünewald & Marahiel, 2006). These chemical modifications along with the cyclization add much to the stability against proteolytic digestion. Although these unusual peptides usually contain fewer than 20 amino and hydroxy acid residues, more than 500 monomer substrates are known to be incorporated in nonribosomal peptides (Caboche et al., 2010).

In addition, CDPs can adopt an α‐helix, β‐sheet, loop or mixed tertiary structure (or random coil). The α‐helix structure is the most common among CDPs, and it is often stabilized by hydrogen bonds between the amide groups and the carboxyl groups of the amino acid residues. The β‐sheet structure is less common in CDPs, but it can be observed in some CDPs with specific sequences. Finally, CDPs can also form complex structures with specific spatial conformations, such as U‐shaped or l‐shaped structures, where hydrophobic interactions and salt bridges (see end of § 3.2.2.3.1) play an important role in their biological activity (Wang et al., 2022). This is the case for valinomycin produced by bacterial strains of *Streptomyces,* a CDP consisting of six (L)‐valine residues (^(L)^Val) and six (D)‐α‐hydroxy isovaleric acid residues (noted as ^(D)^°Val). It adopts a tertiary structure in the form of a crown, which is formed by a self‐winding α‐helix surrounding a potassium ion. This tertiary structure is important for activity as an ionophore antibiotic to transport potassium ion across cell membranes (Huang et al., 2021). Similarly, beauvericin, which can be produced by *Beauveria* and *Fusarium* fungi, is a CDP consisting of six amino acids, with a mixed α‐helix and β‐sheet structure (Liuzzi et al., 2017). Due to this diversity of structural elements, more than 1300 natural CDPs have been described, Taevernier et al. have proposed a detailed classification and a database of these CDPs based on their chemical characteristics (Taevernier et al., 2017). This classification differentiates CDPs based on whether they have one or more ester bonds, whether these multiple ester bonds occur regularly or not, and according to the position of the ester oxygen atom (α, β…).

### 2.2 Functions and origins

CDPs are secondary metabolites that are not essential for organism survival in permissive growth conditions but can confer advantage to their producers by poisoning neighboring organisms in nutrient‐poor environments (Ekman et al., 2012; Raymond et al., 2003). For instance, CDPs can function as cytotoxic potassium ionophores that kill the affected cells, primarily due to the dissipation of membrane potential (Ekman et al., 2012; Huang et al., 2021). CDPs can also act as virulence factors for plant pathogens (Guenzi et al., 1998) or participate in symbiotic relationships between various organisms (Oh et al., 2009; Schoenian et al., 2011; Seipke et al., 2011; Zan et al., 2019). Although many CDPs have been isolated from terrestrial bacteria and fungi (Liu et al., 2023), the main sources of CDPs have been found in marine environments, whose main sources are cyanobacteria, followed by sponges, mollusks, bacteria, fungi and algae (Zeng et al., 2023), detailed below. However, it should be noted that the complex associations of sponges with marine microorganisms make it extremely difficult to define the biosynthetic source (Agrawal et al., 2016).

CDPs can be produced by a wide variety of bacteria, including soil, marine, and pathogenic bacteria. CDPs produced by bacteria can play important roles in (i) interspecies competition, (ii) defense against predators and pathogens and (iii) regulation of symbiotic interaction. The bacterial genera isolated from soil and most frequently associated with the production of these molecules are *Streptomyces* and *Bacillus* (Ribeiro et al., 2022). They produce CDPs to defend themselves against other organisms or for intraspecies competition. Also, marine bacteria and in particular cyanobacteria belonging to the genera *Lyngbya* and *Symploca* produce a wide variety of CDPs, mainly used for their cytotoxic activities (Table 1). Those belonging to the genera *Pseudoalteromonas* produce CDPs involved in defense against predators and pathogens (Taniguchi et al., 2010). Bacteria that live in symbiosis with insects, plants or animals produce CDPs involved in regulation of symbiotic interaction. For example, the entomopathogenic bacterium *Xenorhabdus nematophilus* lives symbiotically in the gut of the nematode, which can infect insects and release bacteria into their hemolymph. The bacteria then proliferate and contribute to the insect's death by producing CDPs (Lang et al., 2008).

**Table 1:** Biological activities of nonexhaustive cyclodepsipeptides from biological sources.

Compound	Exocyclic side chain	Biological source	Biological activity	References
Cyclotridepsipeptides				
Largazole	Exocyclic S‐Acyl substituent on HFA side chain, nonnatural AA	*Symploca* spp	Cytotoxic	Poli et al. (2017)
PM181110	Exocyclic disulfide bridge between two endocyclic viscinal Cys residues	*Phomopsis glabrae*	Cytotoxic	Verekar et al. (2014)
Stereocalpin A	Exocyclic side chain of endocyclic HA	*Stereocaulon alpinum*	Cytotoxic	Byeon et al. (2012)
Cyclotetradepsipeptides				
AM‐Toxins	Exocyclic homo‐benzylic side chain of endocyclic HA unit, nonnatural AA	*Alternaria alternate*	Phytotoxic	Miyashita et al. (2003)
Antillatoxin	Exocyclic 1‐3 dienyl alkyl substituent of endocyclic HA	*Lyngbya majuscula*	Cytotoxic	Burja et al. (2001)
Clavatustides A and B	Endocyclic nonnatural AA	*Aspergillus clavatus*	Cytotoxic	Jiang et al. (2013)
Cryptophycins	Exocyclic aryl‐alkyl substituent of endocyclic HA unit, nonnatural AA	*Nostoc* spp.	Cytotoxic	Schmidt et al. (2020)
Fusarisatin A	Exocyclic dienone alkyl of endocyclic HA unit	*Fusarium graminearum*	Cytotoxic	Hegge et al. (2015)
Geodiamolides A and B	Endocyclic nonnatural AA	*Geodia* sp.	Antifungal	Chan et al. (1987)
Hoiamide A	Hydroxy alkyl of endocyclic heterocyclic HA unit	*Lyngbya majuscula, Phormidium gracile*	Neurotoxic	Pereira et al. (2009)
Jasplakinolide	Endocyclic nonnatural AA	*Jaspis* sp.	Insecticidal, antifungal	Scott et al. (1988)
Lyngbyabellins A and B Dichlorained	Alkyl dichlorine substituent of endocyclic HA unit	*Lyngbya majuscula*	Antifungal	Milligan et al. (2000)
Miuraenamides A‐C	‐	*Paraliomyxa miuraensis*	Cytotoxic	Iizuka et al. (2006)
Neosiphoniamolide A	‐	*Neosiphonia suprtes*	Antifungal	D'Auria et al. (1995)
Cyclopentadepsipeptides				
Alternaramide	‐	*Alternaria* sp. SF‐5016	Antibiotic	Kim et al. (2009)
Apratoxin A	‐	*Lyngbya majuscula*	Cytotoxic	Luesch et al. (2001)
Microspinosamide	Exocyclic N‐Acyl end nonnatural octapeptide amide bonded to endocyclic HAA	*Sidonops microspinosa*	HIV infection inhibitor	Rashid et al. (2001)
neo‐N‐methylsansalvamide	‐	*Fusarium solani* KCCM90040	Cytotoxic	Song et al. (2011)
N‐methylsansalvamide	‐	*Fusarium* (strain CNL‐619)	Cytotoxic	Cueto et al. (2000)
Obyanamide	‐	*Lyngbya confervoides*	Cytotoxic	Williams et al. (2002)
Romidepsin	Disulfide bridge linking Cyst and thioalkyl chain of endocyclic HA unit	*Chromobacterium violaceum*	Cytotoxic	Smolewski and Robak (2017)
Sansalvamide A	‐	Marine *Fusarium*	Cytotoxic	Belofsky et al. (1999)
Tiglicamide A‐C	Exocyclic acyl dipeptide side chain, nonnatural AA	*Lyngbya confervoides*	Protease inhibitor	Matthew et al. (2009)
Tumescenamide C	Exocyclic fatty acid amide linkage with endocyclic HAA	*Streptomyces* sp.	Antimicrobial	Kishimoto et al. (2012)
Unnarmicin A and C	Alkyl chain of endocyclic HA	Extracts from fungi and bacteria	Antifungal	Tanabe et al. (2007)
Zygosporamide	‐	*Zygosporium masonii*	Cytotoxic	Oh et al. (2006)
Cyclohexadepsipeptides				
Beauvericins	‐	*Acremonium* sp.	Cytotoxic, antimalarial	Urbaniak et al. (2019)
Celebeside A	HAA phosphate, HAA carbamate, alkyl substituent of endocyclic HA	*Siliquaria‐spongia mirabilis*	HIV infection inhibitor	Plaza et al. (2009)
Dehydrodidemnin B	Exocyclic (i) alkyl substituent of HA and (ii) N‐Acyl end of dipeptides amide bonded to HAA	*Aplidium albicans*	Cytotoxic	Newman and Cragg (2004)
Desmethylisaridin C1	Alkyl substituent of endocyclic HA	*Beauveria felina*	Antibacterial	Du, Zhang, et al. (2014)
Destruxins	Nonnatural AA, alkyl (or alkenyl, or oxidized) exocyclic side chain,	*Metarrhizium anisoplia*	Insecticidal, phytotoxic	Lange et al. (1991)
Dudawalamide B	Alkynyl substituent of endocyclic HA	*Lyngbya* sp.	Antiparasitic	Almaliti et al. (2017)
Emericellamides A and B	Alkyl substituent of endocyclic HA	*Emericella* sp.	Antibacterial	Oh et al. (2007)
Enniatins	Hydroxy alkyl substituent of endocyclic HA	*Fusarium and verticium* sp.	Mycotoxin, cytotoxic	Sy‐Cordero et al. (2012)
Exumolides A and B	‐	*Scytalidium* sp.	Antimicroalgal	Jenkins et al. (1998)
Guangomides A and B	Exocyclic side chain of endocyclic HA, nonnatural AA	sponge‐derived fungus	Antibacterial	Amagata et al. (2006)
Hantupeptin A	Alkynyl substituent of endocyclic HA	*Lyngbya majuscula*	Cytotoxic	Tripathi et al. (2009)
Himastatin	Nonnatural AAs, covalent dimer	*Strepthomyces hygroscopicus*	Antibacterial	Lam et al. (1990)
Hirsutatin B	‐	*Hirsutella nivea* BCC 2594	Antimalarial	Isaka et al. (2005)
Hirsutellide A	‐	*Hirsutella kobayasii* BCC 1660	Antimycobacterial	Vongvanich et al. (2002)
Isaridin E	‐	*Beauveria felina*	Antibacterial	Du, Zhang, et al. (2014)
Kahalalide F	Exocyclic N‐Acyl end AA amide bonded to endocyclic HAA	*Elysia rufescens*	Antitumor, antiparasitic	Cruz et al. (2009)
Kutznerides	Several nonnatural AAs, alkyl substituent of endocyclic HA	*Kutzneria* sp. 744	Antifungal	Broberg et al. (2006)
Micropeptins	Endocyclic nonnatural AA and amide linkage with exocyclic N‐Acyl AA	Cyanobacterial Bloom	Anti‐inflammatory	Kirk et al. (2021)
Mollemycin A	Glycosylated chain	*Streptomyces* sp.	Antimicrobial	Raju et al. (2014)
Monamycins	Several nonnatural AAs	*Streptomyces jamaicensis*	Antibacterial	Hall (1970)
Nagahamide A	Several nonnatural AAs	*Theonella swinhoei*	Antibacterial	Okada et al. (2002)
Paecilodepsipeptide A	A nonnatural AA	*Paecilomyces cinnamomeus* BCC 9616	Antimalarial, cytotoxic	Isaka, Palasarn, et al. (2007)
Palmyramide A	Alkane substituent of endocyclic HA	*Lyngbya majuscule*	Cytotoxic	Taniguchi et al. (2010)
Pullularins A‐E	Modified and nonnatural AAs	*Pullularia* sp. BCC 8613	Antimalarial, antiviral	Isaka, Berkaew, et al. (2007)
Tasipeptin A	Nonnatural AA, exocyclic N‐Acyl AA amide bonded to endocyclic HAA	*Symploca* sp.	Cytotoxic	Williams et al. (2003)
Tasipeptin B	Endocyclic nonnatural AA, amide llinkage with exocyclic N‐Acyl alkenyl substituents of endocyclic HAs	*Symploca* sp.	Cytotoxic	Williams et al. (2003)
Tiahuramide	Alkynyl substituent of endocyclic HA	*Lyngbya majuscula*	Cytotoxic	Levert et al. (2018)
Tutuilamides A‐C	Exocyclic N‐Acyl end dipeptide amide bonded to endocyclic HAA, nonnatural AA	*Schizothrixsp*	Cytotoxic	Keller et al. (2020)
Salinamides A and B	Exo‐ and endocyclic Nonnatural AAs and exocyclic linkage between two side chains of nonviscinal AAs	*Streptomyces* sp. CNB‐091	Antimicrobial	Moore et al. (1999)
Symplocamide A	Exocyclic N‐Acyl AA amide bonded to endocyclic HAA, nonnatural AA	*Symploca* sp.	Cytotoxic	Linington et al. (2008)
Veraguamide A‐G	Alkynyl substituent of endocyclic HA	*Symploca cf. hydnoides*	Cytotoxic	Salvador et al. (2011)
Cycoheptadepsipeptides				
Aurilide	Alkyl and Alkenyl substituents of endocyclic HAs	*Dolabella auricularia*	Cytotoxic	Kang et al. (2018)
Callipeltin A	Exocyclic N‐Acyl end tetrapeptide amide bonded to endocyclic HAA, nonnatural AAs	*Callipelta* sp.	HIV infection inhibitor	Zampella et al. (1996)
Coibamide A	Exocyclic HA and tripeptide amide bonded to endocyclic HAA	*Leptolyngbya* sp.	Cytotoxic	He et al. (2014)
Dudawalamides A, C and D	Alkynyl substituents of endocyclic HA	*Lyngbya* sp.	Antiparasitic	Almaliti et al. (2017)
HUN‐7293	Nonnatural AAs and alkenyl substituents of endocyclic HAs	Fungal broth	Anti‐inflammatory	Chen et al. (2002)
Lagunamides A and B	Alkyl substituent of endocyclic HA	*Lyngbya majuscula*	Antimalarial	Tripathi et al. (2010)
Mirabamides A‐H	Exocyclic N‐Acyl end nonnatural tetrapeptide amide bonded to endocyclic HAA; monoglycosidic nonnatural AAs	*Siliquariaspongia mirabilis, Stelletta clavosa*	HIV infection inhibitor	Lu et al. (2011); Plaza et al. (2007)
Palau'amide	Nonnatural AAs and Alkyl nitrile substituent of endocyclic HAs	*Lyngbya* sp.	Cytotoxic	Williams et al. (2003a)
Papuamides A and B	Exocyclic N‐Acyl end, nonnatural tripeptide amide bonded to endocyclic HAA and nonnatural AAs	*Theonella mirabilis, Theonella swinhoei*	HIV infection inhibitor	Ford et al. (1999)
Pitipeptolides	Alkyl and various unsaturated alkyl substituents of endocyclic HA	*Lyngbya majuscula*	Antimycobacterial	Montaser, Paul, et al. (2011)
Stellettapeptins A and B	Exocyclic N‐Acyl end, nonnatural tripeptide amide bonded to endocyclic HAA and nonnatural AAs	*Stelletta* sp.	HIV infection inhibitor	Shin et al. (2015)
Ulongapeptin	Exocyclic alkynyl substituent of endocyclic nonnatural AA	*Lyngbya* sp.	Cytotoxic	Williams et al. (2003b)
Cyclooctadepsipeptides				
Aureobasidins	‐	*Aureobasidium pullulans R106*	Antifungal	Awazu et al. (1995)
Bassianolide	‐	*Beauveria bassiana, Verticillium lecanii*	Insecticidal, cytotoxic	Suzuki et al. (1977)
Clavariopsin A and B	Nonnatural AA	*Clavariopsis aquatic*	Antifungal	Kaida et al. (2001)
Emodepside	Aryl substitution of HA	Semi synthetic derivative of PF1022A	Anthelmintic	Ohyama et al. (1999)
Fijimycins A–C	Nonnatural AA, exocyclic amide bond with hydroxy pyridine carboxylic acid	*Streptomyces* sp.	Antibacterial	Sun et al. (2011)
Glomosporin	Hydroxy alkyl substituent of endocyclic HFA	*Glomospora* sp.	Antifungal	Ishiyama et al. (2000)
Grassypeptolide	Nonnatural heterocyclic AA	*Lyngbya confervoides*	Cytotoxic	Kwan et al. (2008)
Homodolastatin 16	Nonnatural AA	*Lyngbya majuscula*	Cytotoxic	Davies‐Coleman et al. (2003)
Homophymine A	Exocyclic HFA, exo and endocyclic nonnatural AAs	*Homophymia* sp.	HIV infection inhibitor	Zampella et al. (2008)
Nobilamide I	Exocyclic tripeptide with N‐Acyl end	*Saccharomonospora* sp	Cytotoxic	Le et al. (2022)
PF1022A	‐	*Mycelia sterilia*	Anthelmintic	Sasaki et al. (1992)
Pitiprolamide A	Bon‐natural AA, exocyclic alkyl of HA	*Lyngbya majuscula*	Cytotoxic	Montaser, Abboud, et al. (2011)
Surfactin	Exocyclic alkyl pf HA	*Bacillus subtilis*	Antibiotic	Horng et al. (2019)
Theopapuamide B‐D	Exocyclic N‐Acyl end, nonnatural tripeptide amide bonded to endocyclic HAA	*Siliquariaspongia mirabilis*	HIV infection inhibitor	Plaza et al. (2009)
Thiocoraline	Exocyclic (i) N‐Acyl A and, (ii) disulfide bridge, endocyclic thioester linkages	*Actinomycete*	Cytotoxic, antimicrobial	Romero et al. (1997)
Verticilide	Several exocyclic alkyl substituents of the endocyclic HAs	*Verticillium* sp. *FKI‐1033*	Insecticidal	Shiomi et al. (2010)
Cyclononadepsipeptides				
Aureobasidin A	‐	*Aureobasidium pullulans*	Antifungal	Takesako et al. (1991)
Cyclolithistide A	Exocyclic N‐Acyl‐AA amide bonded to endocyclic HAA	*Theonella swinhoei*	Antifungal	Clark et al. (1998)
Desmethoxymajusculamide C	‐	*L. majuscula*	Cytotoxic	Simmons et al. (2009)
Majusculamide C	Exocyclic alkyl substituent of HA, nonnatural AA	*Lyngbya majuscula*	Cytotoxic	Robles‐Bañuelos et al. (2022)
Phomafungin	Exocyclic alkyl substituent of HFA, nonnatural AA	*Phoma* sp.	Antifungal	Herath et al. (2009)
Wewakpeptins A‐D	Exocyclic alkynyl substituent of HA	*Lyngbya semiplena*	Cytotoxic	Han et al. (2005)
Cyclodecadepsipeptides				
A54145	Exocyclic N‐Acyl end nonnatural tripeptide amide bonded to endocyclic HAA; nonnatural AAs	*Streptomyces fradiae*	Antibiotic	Miao et al. (2006)
CDA	Exocyclic N‐Acyl AA amide bonded to endocyclic HAA and nonnatural AAs	*Streptomyces coelicolor*	Antibiotic	Hojati et al. (2002)
Daptomycin	Exocyclic N‐Acyl end tripeptide amide bonded to endocyclic HAA; nonnatural AAs	*Streptomyces roseosporus*	Antibiotic	Debono et al. (1988)
Cycloundecadepsipeptides				
Neamphamide A	Exocyclic N‐Acyl end, nonnatural tripeptide amide bonded to endocyclic HAA and nonnatural AAs	*Neamphius huxleyi*	HIV infection inhibitor	Oku et al. (2004)
Neamphamide B	Exocyclic N‐Acyl end, nonnatural tripeptide amide bonded to endocyclic HAA and nonnatural AAs	*Neamphius* sp.	Antimycobacterial	Yamano et al. (2012)
Skyllamycins	Exocyclic aryl‐alkenyl amide bonded to endocyclic HAA and nonnatural AAs	*Streptomyces* sp	Cytotoxic	Giltrap et al. (2018)
Cyclododecadepsipeptides				
Bacillistatins 1 and 2	‐	*Bacillus silvestris*	Cytotoxic, antimicrobial	Pettit et al. (2009)
Cereulide	‐	*Bacillus cereus*	Toxin	Mikkola et al. (1999)
Valinomycin	‐	*Streptomyces* sp.	Antiparasitic	Huang et al. (2021); Matter et al. (2009); Pimentel‐Elardo et al. (2010)
Cyclotridecadepsipeptide				
Petriellin A	Endocyclic nonnatural AAs	*Petriella sordida*	Antifungal	Lee et al. (1995)

*Note*: The structural classification proposed here is based solely on the number of endocyclic amide and ester bonds of the compound.

Abbreviations: AA, amino acid; HA, hydroxy acid; HAA, hydroxy AA; HFA, hydroxy fatty acid.

CDP‐producing fungi are found in a variety of natural habitats such as soils, plants, lichens, tree barks, plant debris and dead insects. Some of them may also be plant or animal pathogens (Sivanathan & Scherkenbeck, 2014). Large and diverse fungal genera produce CDPs with interesting biological properties. Among the known fungal genera are *Acremonium*, *Beauveria*, *Fusarium*, *Aspergillus*, *Altenaria,* and several others that produce CDPs with antifungal, antibacterial, antiviral, and insecticidal properties that can help protect them from pathogens or predators (Table 1).

CDPs can also be isolated from marine sponges. However, the complex associations of sponges with marine microorganisms make it extremely difficult to determine the biosynthetic source (Agrawal et al., 2016). Sponges belong to the *Porifera* phylum and are immobile animals that filter seawater to feed. Several species of sponges have been identified as producers of CDPs, including the genera *Theonella*, *Geodia*, and *Aplidium* among others (Table 1).

### 2.3 Biosynthesis

**Figure 1:**
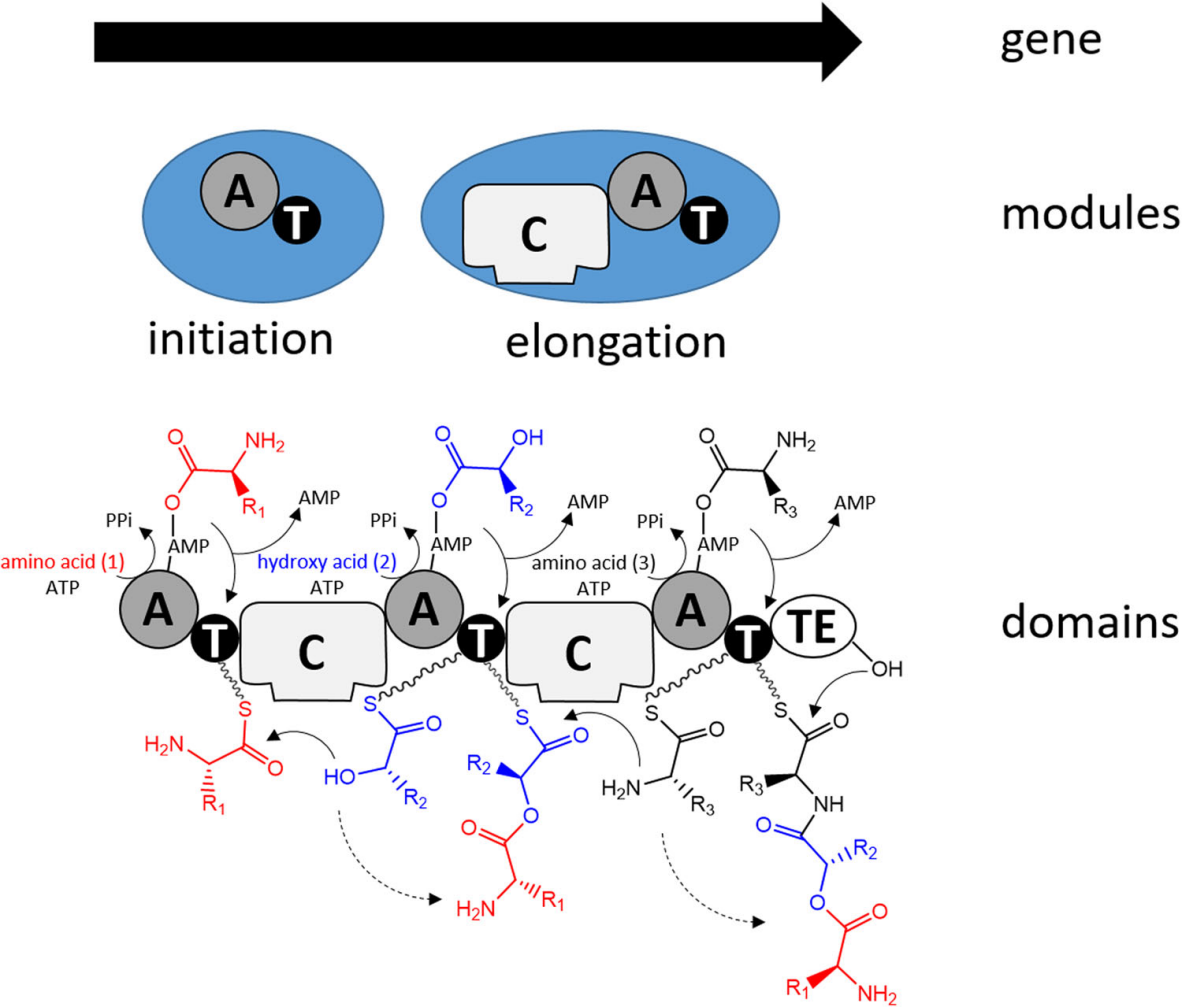
Biosynthesis of depsipeptides by NRPSs. From the gene, modules that are responsible for the incorporation of one amino acid can be identified on the protein level. Modules can be subdivided into domains that harbor catalytic activities. Substrates are activated through reaction with ATP to form an aminoacyl‐AMP intermediate, catalyzed by an adenylation domain (A). The aminoacyl‐AMP intermediate is next transferred to the thiol group of the flexible 4′‐phosphopantetheine arm linked to a thiolation domain (T). Condensation domains (C) catalyze successive amide/ester bond formation between the thioester intermediates loaded onto adjacent T domains. Modules that lack a C domain initiate the nonribosomal peptide synthesis, while those harboring a C domain participate in elongation. A given module accepts a single substrate. Therefore, the number of modules defines the number of building blocks in the final peptide product. The last module contains an additional thio esterase (TE) domain that catalyzes hydrolysis or cyclization to release the final peptide from NRPS.

These nonribosomal peptides are synthesized by large modular multifunctional enzymes known as non‐ribosomal peptide synthetases (NRPSs). Their biosynthesis is different from that of ribosomal proteins since it does not occur through the translation of messenger RNA (mRNA) into an amino acid sequence. NRPSs consist of a set of modules (Finking & Marahiel, 2004) each corresponding to a section of the NRPS polypeptide chain that is responsible for incorporating a specific building block into the elongating polypeptide chain. Modules can in turn be subdivided into domains that have specific enzymatic activities (Figure 1). The first module is the initiation module, which can be further split into an adenylation domain (A) and a thiolation domain (T), also known as peptidyl carrier protein domain. Next comes several elongation modules also containing A and T domains with an additional upstream condensation domain (C). The last module contains a thioesterase (TE) termination domain.

NRPSs require their posttranslational modifications to become catalytically active. The conversion of inactive apoproteins to their active holoforms requires the priming of a conserved serine residue within the T domain by the addition of a flexible 4′‐phosphopantetheine prosthetic group, catalyzed by a 4′‐phosphopantetheinyl transferase. This flexible linker enables attached intermediates to be transferred from one domain to another along the assembly chain. Then, the A domain of the initiation module activates its amino acid substrate while a molecule of ATP is consumed to generate an aminoacyl^−^AMP intermediate, which is attacked by the thiol group of the T domain, leading to the formation of an aminoacyl thioester (Figure 1). The A domain of the second module (elongation module) similarly activates its own substrate (here a hydroxy acid) to generate a second acyl thioester bound to the T domain of the second module. The condensation domain of this second module catalyzes in this instance the formation of an ester bond between the two molecules, which remain linked to the second T domain. The forming depsipeptide is transferred from one module to the other with a single amino/hydroxy acid being added to each module. Finally, the full‐length molecule is released by a terminating thioesterase (TE) domain, which either hydrolyses the linear product or catalyzes cyclisation during the release *via* an ester bond.

In addition to these standard modules, other enzymatic activities may be involved in the maturation of NRPS products. These additional enzymes can modify the peptide backbone, by performing C‐ and N‐methylation (Chow et al., 2020; Maruyama et al., 2020), or halogenation (Matthew, Salvador, et al., 2010), which enlarge the structural diversity of depsipeptides.

### 2.4 Genomic organization of NRPS

Modules are usually distributed over several NRPSs whose genes are organized in operon‐like structures, where the substrate activating/modifying units are aligned in a sequence that is (usually) collinear with the amino acid sequence of the assembled peptide (Stachelhaus & Marahiel, 1995). Several genes can encode various modules in which each module bears condensation, adenylation, and thiolation domains.

**Figure 2:**
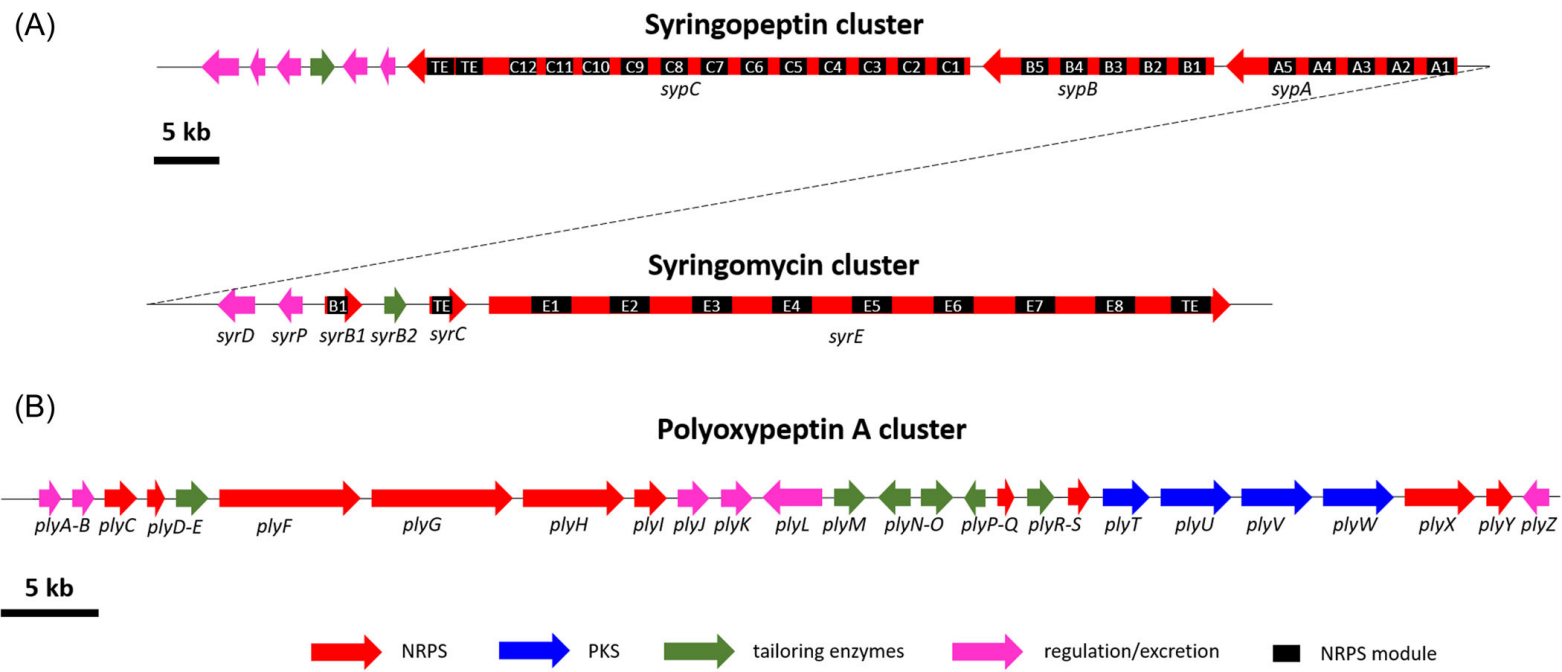
Clusters of genes involved in cyclodepsipeptide biosynthesis. The positions and orientations of the known genes are shown as horizontal arrows. Their putative functions are indicated by color‐labeling. (A) Organization of the chromosomic region in *Pseudomonas syringae pv. syringae* B301D containing both syringomycin (syr) and syringopeptin (syp) gene clusters. (B) the polyoxypeptin A biosynthetic gene cluster. Figure adapted from Wang, Lu, Wang, et al. (2006) and Du, Wang, et al. (2014).

This organization is illustrated in the bacterium *Pseudomonas syringae*, which produces the phytotoxins syringopeptin and syringomycin. These CDPs have similar structures consisting of cyclic peptide heads attached to 3‐hydroxy fatty acid tails. The syringomycin (*syr*) and syringopeptin (*syp*) gene clusters (Figure 2A) are located adjacent to one another on the chromosome and are approximately 42 and 90 kb in size, respectively (Scholz‐Schroeder et al., 2003). The two gene clusters include biosynthesis, regulatory, and secretion genes for the production of both phytotoxins. Syringopeptin is assembled from three large open reading frames, *sypA*, *sypB*, and *sypC*, that are 16.1, 16.3, and 40.6 kb in size. They code for peptide synthetases that contain five, five, and 12 amino acid activation modules, respectively. Each module exhibits characteristic domains for condensation, aminoacyl adenylation, and thiolation. The syringopeptin synthetases, which carry 22 NRPS modules, represent the largest linear NRPS system described for a prokaryote (Scholz‐Schroeder et al., 2001). For the synthesis of syringomycin, the nine modules involved in the binding of the nine amino acids are localized on SyrB and SyrE, the latter carrying eight modules. The genetic organization of syringomycin synthetase is unusual since it does not respect the “colinearity rule” (the order of the amino acid binding modules along the chromosome does not parallel the order of the amino acids on the peptide). *SyrB* encodes two proteins; SyrB1 that carries the typical adenylation and thiolation domains and which recognizes the last amino acid of syringomycin, and SyrB2, a halogenase. *SyrC* encodes a thioesterase, a protein often found at one end of bacterial peptide synthetase operons. *SyrD*, transcribed in the opposite orientation with respect to *syrB* and *syrC* is involved in syringomycin excretion; SyrP codes for a regulatory protein (Wang, Lu, Yang, et al., 2006).

To further illustrate the complexity of CDP synthesis, NRPS assembly chains, in addition to using a wide range of unusual amino acids, further add structural diversity through the coordinated action of sets of dedicated tailoring enzymes. Some of these tailoring enzymes act after the peptide has been released from its assembly chain, while others act during chain elongation whereas the growing peptide chain is attached to specific checkpoints of the T domain. Sometimes the adapting enzymes during peptide chain synthesis act as separate subunits, in *trans*, or they are integrated into the assembly chain of the relevant module where adaptation occurs, thus acting in *cis* (Walsh et al., 2001). NRPS systems are very often combined with polyketide synthases to make hybrid peptide‐ketide products. Polyketide synthases catalyze the assembly of complex natural products from simple precursors (e.g., propionyl‐CoA and methyl‐malonyl‐CoA) in a biosynthetic process that resembles fatty acid biosynthesis (Khosla et al., 1999). Such a combination of biosynthetic processes further increases chemical diversity (Alonzo & Schmeing, 2020).

The synthesis of polyoxypeptin A (PLYA) by *Streptomyces* sp. MK498‐98 F14 is representative of the complexity that CDP biogenesis could require (Du, Zhang, et al., 2014). No fewer than 37 open reading frames that span 75 kb in the chromosome are anticipated to constitute the *ply* gene cluster. Among them, four modular type I PKS genes (*ply*TUVW) and four modular NRPS genes (*ply*XFGH) encoding four PKS modules and six NRPS modules are present for the assembly of the PLYA core structure (Figure 2B). Other six NRPS genes (*ply*CDQISY) encode an A domain, two PCPs, and three TEs. There are at least six genes (*ply*EMOPR) encoding tailoring enzymes annotated as hydroxylases or monooxygenases and an aminotransferase (*ply*N), thought to be involved in the biosynthesis of unusual building blocks or post‐modifications.

### 2.5 Exploitation of NRPS‐based biosynthetic pathways to produce new CDPs

Starting in the 1949s, drugs derived from secondary metabolite have become crucial for curing infectious diseases (Clardy et al., 2006; Nussbaum et al., 2006). Due to the complexity of their structures, many of these compounds are chemically modified after their isolation from biological sources (Kirschning & Hahn, 2012).

Thus, because of the modular nature of NRPS, it is appealing to reprogram NRPS systems to design assembly lines that ideally could provide peptide structures of choice. Many efforts to challenge the functions or biosynthetic pathways for the synthesis of novel or modified NRP structures have been made (*vide infra*). These efforts are mainly based on the following methods: chemoenzymatics, precursor‐directed biosynthesis, mutasynthesis and combinatorial biosynthesis.

**Chemoenzymatics** techniques use enzyme preparations (single domains/modules, native NRPS, and tailoring enzymes). As an illustration, CDP analogues have been synthesized using enniatin (Feifel et al., 2007), PF1022 (Müller et al., 2009), and beauvericin synthetase (Matthes et al., 2012).

**Precursor‐directed biosynthesis** provides synthetic building blocks to wild‐type strains with an intact biosynthetic assembly chain. A nice demonstration is its application to halogenated compounds by varying the media for derivatives of enniatins (Krause et al., 2001) and beauvericin (Xu et al., 2007).

**Mutasynthesis** makes use of auxotroph strains with impaired building block biosynthesis. The disrupted pathway can be reactivated by feeding synthetic analogues of the missing intermediate. For example, in mutants of the CDA (see Table 1) biosynthesis, resulted in the directed biosynthesis of novel CDPs (Hojati et al., 2002).

With **combinatorial biosynthesis**, NRPSs are modified at different levels of the assembly line, such as module fusion that led to the synthesis of daptomycin derivatives (Doekel et al., 2008). In another study, hybrid genes between the daptomycin and A54145 biosynthetic pathways were constructed and led to the production of biologically active hybrid molecules (Nguyen et al., 2010).

## 3 STRUCTURAL CHARACTERIZATION OF CDPs

CDPs are an interesting and promising class of natural products displaying a variety of biological activities such as antimicrobial (Fotso et al., 2018; Maharani et al., 2014; Ojima et al., 2016), antiviral (Qi et al., 2016; Yeh et al., 1996; Zampella et al., 2008), antimalarial (Tripathi et al., 2010), antitumor or cytotoxic (Boudreau et al., 2012; Chen et al., 2014; Kwan et al., 2010; Lee & Lee, 2012; Levert et al., 2018; Li, Yu, et al., 2020; Martín et al., 2014; Mevers et al., 2011; Nakamukai et al., 2018; Ohsawa et al., 2022; Terracciano et al., 2005; Walser et al., 2021) properties and activities (Gala et al., 2009; Kang et al., 2022; Keller et al., 2020; Matthew et al., 2009; Matthew, Ratnayake, et al., 2010; Salvador et al., 2013) (see Table 1 for a nonexhaustive sample panel). Their intriguing molecular scaffold, featuring at least one endocyclic ester linkage, has attracted much attention for the development of potential therapeutic agents. Predominantly isolated from marine cyanobacteria, a number of CDP analogues have also been chemically synthesized for different purposes such as full stereostructure validation (Martín et al., 2014; Reimann et al., 2017), concise and efficient production of pure CDP originally isolated from a biological extract (Maharani et al., 2014; Ohsawa et al., 2022; Ojima et al., 2016; Qi et al., 2016), or in the hope of improving the biological properties of the original lead compound (Terracciano et al., 2005).

Considering the importance of this class of natural bioactive macrocycles in medicinal chemistry and drug design, fine structural characterization is essential as it allows the linking of the substance structure to its potential biological activity. In this context, and as a complement to high‐resolution mass spectrometry (HRMS), NMR remains the technique of choice for finely characterizing the structure of CDPs (especially their stereochemistry), whether isolated from natural environments or chemically synthesized.

### 3.1 NMR: From the discovery of rare structural residues to the complete configurational assignment of CDPs

Various NMR experiments may be carried out to give access to the organization of atoms in relation to each other in a chemical structure, including in some cases their 3D arrangement for asymmetrical centers. The one‐dimensional experiment (1D NMR) is first used: the ^1^H NMR because of its sensitivity and the ^13^C NMR because it offers a higher chemical shift dispersion facilitating signal assignments in complex structure elucidation. Multidimensional experiments such as Correlation Spectroscopy (COSY), Total Correlation Spectroscopy (TOCSY), and Heteronuclear Multiple Bond Correlation (HMBC), the most common 2D‐NMR experiments, are then usually performed to refine peak assignment and resolve spectral overlapping. The ^1^H‐^1^H Nuclear Overhauser Effect Spectroscopy (NOESY) and Rotating‐frame Overhauser enhancement spectroscopy (ROESY) experiments are particularly useful for accessing conformational information, as they allow the spatial proximity of protons in the molecule to be visualized. These experiments are particularly well suited to the study of conformationally constrained macrocycles such as CDPs.

In addition to their association with MS, NMR experiments are even more effective for the complete elucidation of CDP structures when combined with high‐performance liquid chromatography (HPLC) analysis of derivatized hydrolysates according to the Marfey's method (Fujii et al., 1997). The later approach consists in performing the following steps: (i) acid hydrolysis of the CDP; (ii) derivatization of the cleaved amino acids with 1‐fluoro‐2,4‐dinitrophenyl‐5‐l‐alanine amide (FDAA); and (iii) HPLC analysis and retention time comparison with the adequate l‐ or d‐amino acid standards (e.g., ^(L)^Val or ^(D)^Val) previously subjected to the same derivatization process. This method is widely used as a complement to NMR notably for the stereo‐analysis of the amino acid residues constituting the macrocycle. This is the method of choice for finely characterizing each constituent residue of cyclic peptides, since Edman's protocol (Edman, 1949; Gomes, 2022) is not applicable to CDPs, which lack a free N‐terminus, as mentioned in the introduction.

Table 2 shows a series of nonexhaustive examples of CDPs mainly characterized by advanced NMR analysis. Some of the structures have been validated based on comparison with NMR spectra of previously identified compounds (e.g., destruxin E2 chlorohydrin (Yeh et al., 1996), largamide D oxazolidine (Matthew, Ratnayake, et al., 2010), grassypeptolides A‐C (Kwan et al., 2010), and cladoamide A and B (Liao et al., 2022)), while others have been subjected to entirely new NMR signal assignments (e.g., homophymine A (Zampella et al., 2008), tiglicamides A–C (Matthew et al., 2009), stellatolides A‐G (Martín et al., 2014)).

**Table 2:** Nonexhaustive list of CDPs whose structures have been elucidated mainly by a series of NMR experiments. AA is used for amino acid.

CDP name(s)	Mode of production (biological or chemical)	Type of macrocycle	NMR analysis in complement to 1D ^1^H and ^13^C NMR	Additional experiments (MSn, IR, UV, etc.)	Molecular Formula	Main structural results and particularities (e.g. planar structure, epimer, conformational study, etc)	Activity/testing	Reference
Destruxin E2 chlorohydrin	Extraction from the culture medium of *Metarrhizium anisopliae*	19‐membered cyclohexadepsipeptide	^1^H‐^1^H COSY and ^1^H‐^13^C COSY, ^1^H‐^1^H TOCSY	MS (1 Cl identified) Molecular modeling	C_28_H_46_CIN_5_O_8_	− Identification by comparison with ^1^H‐NMR spectral data of previously reported destruxins and in particular the close analog destruxin E − Chlorinated derivative − Hydrophobicity in the convex surface of destruxins	Suppressive activity on the production of HBV surface antigen in human hepatoma Hep3B cells	Yeh et al. (1996)
Jaspamide analogues (2–7)	Chemical synthesis of 6 new jaspamide analogues	16‐ to 19‐membered cyclotetra‐ and penta‐depsipeptide	HMBC, HSQC, TOCSY, COSY, and ROESY	ESI‐MS for molecular formula validation	2: C_30_H_36_N_6_O_5_ 3: C_28_H_33_N_5_O_4_ 4: C_30_H_37_N_5_O_4_ 5: C_31_H_38_N_6_O_6_ 6: C_33_H_34_N_4_O_7_ 7: C_29_H_34_N_4_O_6_	− Partial to poor superimposition of the new analogues with the X‐ray structure of jaspamide according to conformationnal study − Lack of antitumor activity compared to the parent molecule jaspamide	Poor antitumor activity profiles compared to jaspamide	Terracciano et al. (2005)
Homophymine A	Isolatation from a New Caledonian collection of the marine sponge *Homophymia* sp.	25‐membered cyclooctadepsipeptide	^15^N‐^1^H HSQC, COSY, TOCSY, HSQC, HMBC, and ROESY	LC‐ESI‐MS, LC‐MS/MS and chiral HPLC analysis	C_73_H_127_N_15_O_24_	− Elucidation of four uncommon AAs: (2 S,3 S,4 R)−3,4‐diMeGln, NMeGln, pipecolic acid and l‐ThrOMe; and an amide‐linked HTMOA − Absolute stereochemistry determination with: complete acid hydrolysis and Marfey's analysis/chiral HPLC analysis of the hydrolysate mixture/J‐based NMR configurational analysis and chemical derivatization methods	Cytoprotective activity against HIV‐1 infection	Zampella et al. (2008)
Jaspamides M–P	Isolation from the marine sponge *Jaspis splendans* of four new jaspamide derivatives	19‐membered cyclotetradepsipeptide	COSY, TOCSY, HSQC and HMBC	HR‐ESI‐MS, [α]^25^_D_	M: C_35_H_43_BrN_4_O_6_ N: C_36_H_45_BrN_4_O_7_ O: C_35_H_46_N_4_O_7_ P: C_37_H_48_N_4_O_9_	− Identification of four tryptophan modified jaspamide derivatives including two brominated compounds − AA absolute configurations determined by LC/MS analysis of the acid hydrolysate derivatized with Marfey's reagent	Antimicrofilament activity	Gala et al. (2009)
Tiglicamides A–C	Isolation from the Floridian marine cyanobacterium *Lyngbya confervoides*	16‐membered cyclopentadepsipeptide	COSY, TOCSY, ROESY, HSQC, and HMBC	HR‐ESI/APCI‐MS and chiral HPLC	A: C_45_H_59_N_7_O_13_ B: C_44_H_57_N_7_O_12_ C: C_40_H_57_N_7_O_13_S	− Planar structures determined by 1D and 2D NMR combined with MS − Absolute configurations: chiral HPLC and Marfey's analysis − Presence of one or two modified AA residues such as 2‐amino‐2‐butenoic acid (Abu), and homotyrosine (Htyr) for A and methionine sulfoxide (Met(O)) for C; and an unusual tiglic acid (Tig) moiety.	Inhibit porcine pancreatic elastase	Matthew et al. (2009)
Largamide D oxazolidine	Extraction from Floridian *Lyngbya* cf. *confervoides* samples	19‐membered cyclohexadepsipeptide	COSY, TOCSY, ROESY, HSQC, and HMBC	LC‐ESI‐HRMS	C_56_H_80_BrN_9_O_16_	− The new structure results from Largamide D intramolecular condensation − Same configuration of the new oxazolidine as reported for largamide D for all unchanged residues − Conformation study evidenced by ROESY correlations	Antifeedant activity (Serine protease inhibitory)	Matthew, Ratnayake, et al. (2010)
Lagunamides A and B	Isolation from the marine cyanobacterium *Lyngbya majuscula*	26‐membered cycloheptadepsipeptide	COSY, HMBC, TOCSY, NOESY, ROESY	ESI‐HRMS, [α]^25^_D_, UV and IR spectroscopy	A: C_45_H_71_N_5_O_10_ B: C_45_H_69_N_5_O_10_	− Configuration: advanced Marfey's analysis of AAs − Importance of the choice of deuterated solvent for ^1^H and ^13^C NMR analysis: coexistence of conformers vs. one major conformer	Tested for antimalarial, antiswarming and cytotoxic properties	Tripathi et al. (2010)
Grassypeptolides A‐C	Isolation from the Floridian marine cyanobacterium *Lyngbya confervoides*	31‐membered cyclooctadepsipeptide	COSY, ROESY, HSQC, and HMBC	HR‐ESI/APCI‐MS Chiral HPLC X‐ray crystallography	A: C_56_H_79_N_9_O_10_S_2_ B: C_55_H_77_N_9_O_10_S_2_ C: C_56_H_79_N_9_O_10_S_2_ (28‐epi‐A)	− Identification of two new compounds (among which one epimer of the previously isolated and characterized grassypeptolide A) − Structures include a rare β‐AA (2‐methyl‐3‐aminobutyric acid, Maba), a large number of d‐AAs, and extensive N‐methylation − Evidence of metal binding properties: binding to Cu^2+^ and Zn^2+^	Antiproliferative activity	Kwan et al. (2010)
Xeraguamides A‐C and H‐L	Isolation from the marine cyanobacterium cf. *Oscillatoria margaritifera*	19‐membered cyclohexadepsipeptide	COSY, TOCSY, ROESY, HSQC and HMBC	HR‐ESI‐MS, IR spectroscopy GC‐MS MS2 and MS3	A: C_37_H_59_N_4_O_8_Br B: C_36_H_57_N_4_O_8_Br C: C_37_H_60_N_4_O_8_ H: C_36_H_58_N_4_O_8_ I: C_37_H_64_N_4_O_8_ J: C_36_H_62_N_4_O_8_ K: C_38_H_65_N_4_O_9_Br L: C_38_H_63_N_4_O_9_Br	− AA absolute configurations determined by LC‐MS analysis of the acid hydrolysate derivatised with Marfey's reagent or by GC‐MS or by hydrogenation techniques depending on the aa residue − Two of the newly identified veraguamides (C and H) contain the same terminal alkyne moiety as the mollusk‐derived kulomo'opunalide natural products − Four novel veraguamides (A, B, K and L) feature an unusual alkynyl bromide functionality that has only been previously observed in jamaicamide A	Cytotoxic activity	Mevers et al. (2011)
Neosansalvamide	Isolation from *Fusarium*‐contaminated potato in Korea	15‐membered cyclopentadepsipeptide	DEPT, COSY, HMQC, HMBC and NOESY	IR and UV spectroscopy ESI − LC − MS and MS/MS	C_32_H_50_N_4_O_6_	AA absolute configuration: RP‐HPLC analysis of the Marfey's method degradation products	Cytotoxic activity tested on human cancer cell lines	Lee and Lee (2012)
Viequeamide A	Isolated from *Rivularia* sp. marine cyanobacteria	22‐membered cycloheptadepsipeptide	TOCSY, COSY, HMBC, HSQC, ROESY	HRMS GC‐MS (Hmpa config.)	C_42_H_69_N_5_O_10_	l‐AA absolute configuration determined by hydrolysis, derivatization, and chromatography in comparison with standards (Marfey's analysis)	High cytotoxic activity on H460 human lung cancer cells (IC50 = 60 ± 10 nM)	Boudreau et al. (2012)
Symplostatin 5‐10	Extraction from the red *Symploca* sp. marine cyanobacteria	19‐membered cyclohexadepsipeptide	COSY, TOCSY, HSQC and HMBC	ESI‐MS, enantioselective HPLC‐MS, UV spectroscopy, [α]^20^_D_	5: C_47_H_65_N_7_O_15_S 6: C_46_H_63_N_7_O_15_S 7: C_48_H_67_N_7_O_15_S 8: C_47_H_65_N_7_O_16_S 9: C_46_H_63_N_7_O_16_S 10: C_48_H_67_N_7_O_16_S	Stereocenter absolute configurations determined by enantioselective HPLC‐MS analysis of acid hydrolysates with and without prior oxidation of the Ahp unit	Inhibit the proteolytic activity of elastase	Salvador et al. (2013)
Calcaripeptides A − C	Isolation from *Calcarisporium* sp. (German Wadden Sea)	14‐ and 16‐membered cyclodidepsipeptide	DEPT, COSY, HSQC, HMBC and NOESY	HR‐ESI‐MS, single‐crystal X‐ray diffraction analysis	A: C_27_H_37_N_2_O_5_ B: C_26_H_35_N_2_O_5_ C: C_24_H_33_N_2_O_5_	− Macrocyclic structures consisting of Pro, Phe and a nonpeptidic substructure − Absolute configuration at stereogenic centers determined by X‐ray analysis − ^13^C‐labeled feeding experiments to study the biosynthetic origin of the structures	No activity testing	Silber et al. (2013)
Aureobasidin L	Total chemical synthesis	27‐membered cyclopentadepsipeptide	DEPT, COSY, HSQC, HMBC and NOESY	HR‐ESI‐MS, MS2	C_59_H_89_N_8_O_10_	− Combination of solution‐phase and solid‐phase peptide synthesis − Absolute configuration of stereocenters controled during the synthesis	Antifungal properties	Maharani et al. (2014)
Apratoxin S4, S7‐9	Isolation from marine cyanobacteria	25‐membered cyclononadepsipeptide	No additional NMR experiments	HR‐ESI‐MS	S4: C_44_H_69_N_5_O_8_S S7: C_43_H_67_N_5_O_8_S S8: C_45_H_71_N_5_O_8_S S9: C_44_H_69_N_5_O_8_S	− Synthesis with improved protocoles − Absolute configuration of stereocenters controled during the synthesis	Anticancer agent	Chen et al. (2014)
Stellatolides A‐G	Isolation from the Sponge *Ecionemia acervus* + Solid‐phase synthesis of stellatolide A	22‐membered cycloheptadepsipeptide	COSY, TOCSY, HSQC, HMBC and ROESY	HR‐ESI‐MS	A: C_66_H_107_N_15_O_22_ B: C_65_H_105_N_15_O_22_ C: C_66_H_109_N_15_O_23_ D: C_66_H_107_N_15_O_21_ E: C_65_H_105_N_15_O_22_ F: C_66_H_104_N_14_O_21_ G: C_66_H_104_N_14_O_22_	− First multigram scale total synthesis of stellatolide A by solid‐phase method − Full stereochemical assignment: total synthesis of Stellatolide A + HPLC and Marfey's analysis	In vitro antiproliferative activity on human tumor cell lines	Martín et al. (2014)
Miuraenamides A and D	Total synthesis (previously isolated from *Paraliomixa miuraensis*)	19‐membered cyclotetradepsipeptide	No additional NMR experiments	HR‐ESI‐MS, IR spectroscopy, [α]^23^_D_	A: C_34_H_42_BrN_3_O_7_ D: C_34_H_42_BrN_3_O_7_	− Linear 20‐steps synthesis (3.2% overall yield) Absolute configuration of stereocenters controlled during the synthesis	Antimicrobial and antitumor activity	Ojima et al. (2016)
Aetheramides A and B	Total synthesis (originally isolated from myxobacteria *Aetherobacte*r)	22/21‐membered cyclotridepsipeptide	No additional NMR experiments	HR‐ESI‐MS, [α]^27^_D_	A: C_41_H_54_N_2_O_9_ B: C_41_H_54_N_2_O_9_	− Linear 15‐steps synthesis (4.7% overall yield) − Absolute configuration of stereocenters controlled during the synthesis	Anti‐HIV agents	Qi et al. (2016)
Fusaricidin E and F	Isolation from fermentation of *Paenibacillus* strains + Total synthesis	19‐membered cyclohexadepsipeptide	COSY, NOESY, HMBC and HSQC	ESI‐HRMS, APCI–MS–MS, [α]^24^_D_	E: C_47_H_78_N_10_O_12_ F: C_46_H_76_N_10_O_12_	Full stereostructure of fusaricidin E isolated from strain confirmed by total synthesis	No activity testing	Reimann et al. (2017)
Alveolarides A‐C	Isolation from *Microascus alveolaris*	17‐membered cyclopentadepsipeptide	COSY, HSQC, HMBC, H2BC, HSQC‐TOCSY, 1,1‐ADEQ and NOESY	HRMS, IR spectroscopy	A: C_43_H_70_N_6_O_12_ B: C_43_H_68_O_6_N_12_ C: C_43_H_60_N_6_O_9_	− AA absolute configuration: acid hydrolysis and Marfey's Analysis − Partial absolute configuration established for alveolaride A − Three novel CDP with a rare 2,3‐dihydroxy‐4‐methyltetradecanoic acid unit and a β‐phenylalanine AA residue	In vitro antifungal activity against plant pathogens	Fotso et al. (2018)
Stellatolide H	Isolation from a deep‐sea sponge *Discodermia* sp.	22‐membered cycloheptadepsipeptide	ROESY, HMBC, COSY	HR‐ESI‐MS	C_66_H_106_N_14_O_21_	− Identification of a peptide lactone of the callipeltin class characterized by the presence of the unusual Me_2_Gln residue. − N‐terminus blocked by 3‐hydroxy‐6,8‐dimethyldeca‐(4Z,6E)‐dienoic acid (Hdda) − AA absolute configuration determined by the Marfey's method in combination with LC‐MS detection	Cytotoxic activity against HeLa cells	Nakamukai et al. (2018)
Tiahuramides A − C	Isolation from the marine cyanobacterium *Lyngbya majuscula*	19‐membered cyclohexadepsipeptide	COSY, HOHAHA, HSQC, HMBC and ROESY	HRMS, UV and IR spectroscopy, HPLC, [α]^25^_D_	A: C_41_H_60_N_4_O_8_ B: C_41_H_62_N_4_O_8_ C: C_41_H_64_N_4_O_8_	− AA absolute configuration determined by a combination of acid hydrolysis, derivitization with Marfey's reagent and HPLC − Absolute configuration of hydroxy acids confirmed by Mosher's method	Antibacterial activity + Cytotoxic activity tested in SH‐SY5Y human neuroblastoma cells	Levert et al. (2018)
Tutuilamides A–C	Isolation from cyanobacterium *Schizothrix* sp. (A and B) and *Coleofasciculus* sp. (C)	19‐membered cyclohexadepsipeptide	HSQC, HMBC, TOCSY, ROESY, DQF‐COSY, 1,1‐ADEQUATE and NOE	HR‐ESI‐MS, HPLC and chiral derivatization	A: C_51_H_69_ClN_8_O_12_ B: C_50_H_67_ClN_8_O_12_ C: C_48_H_64_ClN_7_O_11_	− Presence of unusual residues such as 3‐amino‐6‐hydroxy‐2‐piperidone and 2‐amino‐2‐butenoic acid and a novel vinyl chloride‐containing residue − AA absolute configuration determined by acid hydrolysis, derivatization with d‐FDAA and Marfey's Analysis − Strategy for the discovery of novel structures includes “molecular networking” (MS2‐based metabolomics)	Elastase inhibitory properties	Keller et al. (2020)
Pagoamide A	Isolation from marine Chlorophyte, *Derbesia* sp.	19‐membered cyclohexadepsipeptide	HMBC, COSY, ROESY	HR‐ESI‐MS, MS/MS, UV spectroscopy, [α]^25^_D_	C_51_H_67_N_11_O_12_S_2_	− Absolute configuration determined by Marfey's analysis following chemical hydrolysis or hydrazinolysis + DFT calculations in combination with ROESY results − Isolation of the CDP guided by MS/MS‐based molecular networking	Inactive in the calcium oscillation and H‐460 human lung cancer cell cytotoxicity assays	Li, Yu, et al. (2020)
Isocereulide A	Isolation from anaerobe bacterium *Bacillus cereus*	36‐membered cyclododecadepsipeptide	COSY, HSQC, HMBC, DEPT	UPLC‐ESI‐MS, MSn sequencing	C_58_H_102_N_7_O_18_	− Revision of the previously described structure of isocereulide A with a ^(L)O^Ile unit instead of a ^(L)O^Leu residue − Absolute stereo configuration determined by Marfey's analysis	Highly cytotoxic	Walser et al. (2021)
Epoxinnamide	Isolation from intertidal mudflat‐derived *Streptomyces* sp.	31‐membered cyclodecadepsipeptide	HSQC, COSY, TOCSY, HMBC, ROESY	UV and IR spectroscopy, HR‐ESI‐MS,	C_62_H_79_N_11_O_20_	− Identification of a structure consisting in six proteinogenic AA, four unusual AA, and an acyl chain of o‐1,2‐epoxypropyl cinnamic acid + Bicyclic diaryl ether linkage − Absolute configuration was determined by the combination of Marfey's method, 3JHH and ROESY analysis, DP4 calculation, and genomic analysis	QR activity in murine Hepa‐1c1c7 cells and antiangiogenesis activity in human umbilical vein endothelial cells	Kang et al. (2022)
Decatransin	Total synthesis (originally isolated from the saprophyte fungus *Chaetosphaeria tulasneorum*)	30‐membered cyclodecadepsipeptide	No additional NMR experiments	HR‐ESI‐MS, UPLC, IR spectroscopy, [α]^22^_D_	C_63_H_109_N_9_O_12_	− One of the key synthetic step is a macrocyclization under Mitsunobu conditions − CDP consisting of eight nonproteinogenic AAs and 2‐hydroxy‐5methylhexanoic acid (HA) − Comparisons of NMR spectroscopic data (DMSO‐d_6_) between isolated decatransin and the synthetic compound	Cytotoxicity against HCT‐116 cells	Ohsawa et al. (2022)
Destruxin B, pseudodestruxin B, isaridin A, cladoamide A and cladoamide B	Total synthesis (originally isolated from fungi)	19‐membered cyclohexadepsipeptide	NOESY	HR‐ESI‐MS, IR spectroscopy, [α]^28^_D_	C_36_H_55_N_5_O_7_ C_36_H_55_N_5_O_7_ C_39_H_53_N_5_O_7_ C_40_H_55_N_5_O_7_ C_39_H_53_N_5_O_7_	− First total synthesis of four CDPs analogs of destruxin B − Investigation of a novel flow macrocyclization approach at three different cyclization points − Comparison of ^1^H and ^13^C NMR data with those of the natural compounds previously reported in the literature	No activity testing	Liao et al. (2022)

**Figure 3:**
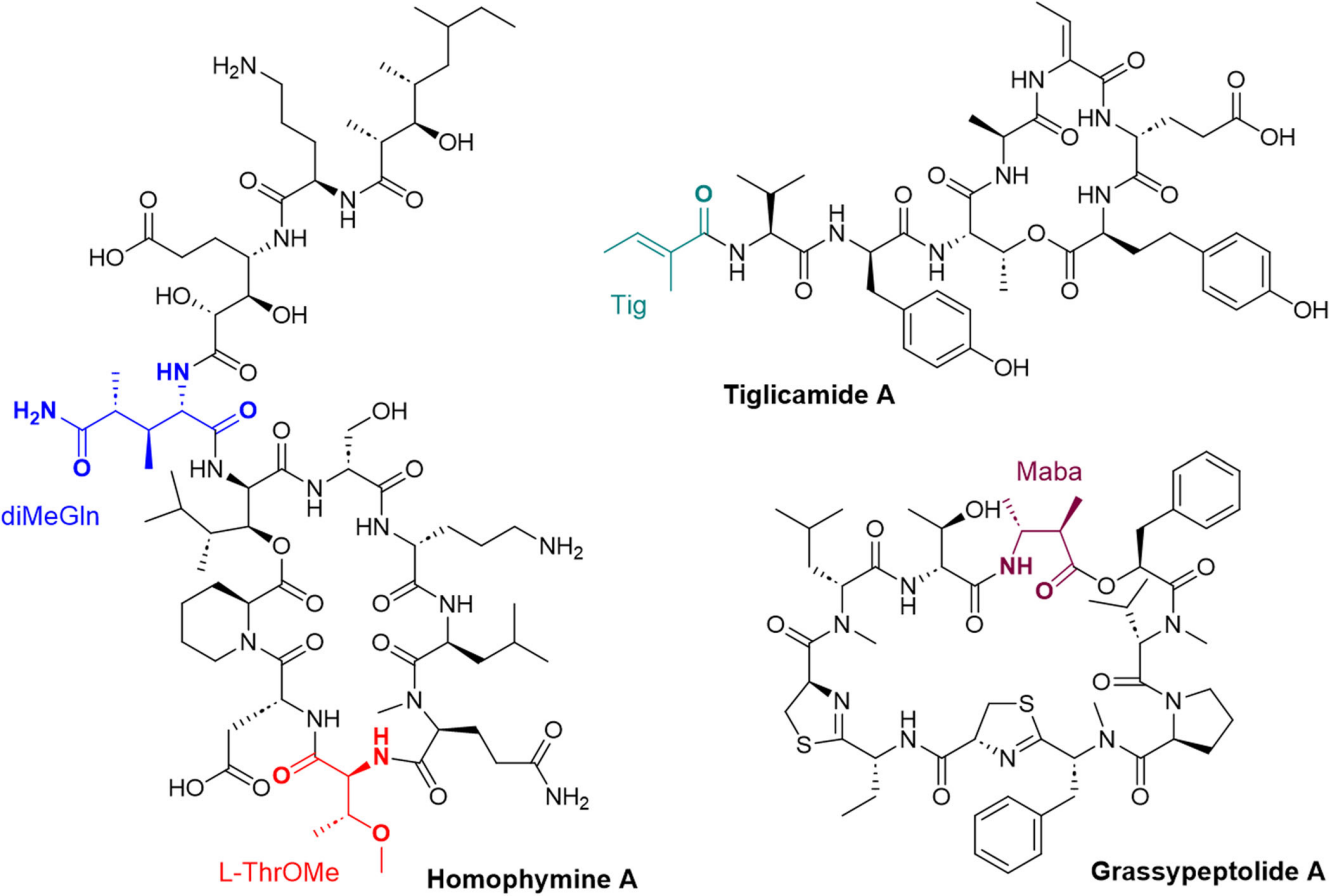
Structures of some CDPs (tiglicamide A, homophymine A and grassypeptolide A) featuring unusual residues.

The various NMR experiments that were carried out, revealed the presence of rare amino acids or previously unknown residues in some CDP structures: homophymine A (Figure 3), a 25‐membered cyclooctadepsipeptide isolated from the marine sponge *Homophymia* sp. was characterized by Zampella et al. and features four uncommon amino acids, including (2 S,3 S,4 R)−3,4‐dimethylglutamine (diMeGln) and ^(L)^ThrOMe (Zampella et al., 2008). The unusual dimethylglutamine residue (diMeGln), characteristic of the callipeltin class, has also been identified in the 22‐membered macrocycles of the stellatolide family, as illustrated by stellatolide A‐G reported in 2014 by Martin et al. (2014), or by stellatolide H reported in 2018 by Nakamukai et al. (2018). The 16‐membered cyclopentadepsipeptides tiglicamides A (Figure 3) and B‐C, isolated from the Floridian marine cyanobacterium *Lyngbya confervoides*, feature an unusual tiglic acid (*Tig*) moiety (Matthew et al., 2009). Also, the three 31‐membered cyclooctadepsipeptides grassypeptolides A (Figure 3) and B‐C, isolated from the same cyanobacterium, contain several d‐amino acids and one rare β‐amino acid, namely 2‐methyl‐3‐aminobutyric acid (Maba) (Kwan et al., 2010). In 2018, three new CDPs isolated from *Microascus alveolaris*, namely alveolarides A‐C, were shown to possess a rare 2,3‐dihydroxy‐4‐methyltetradecanoic acid unit and a β‐phenylalanine amino acid residue (Fotso et al., 2018). More recently, NMR experiments carried out on compounds isolated from cyanobacterium *Schizothrix* sp. and *Coleofasciculus* sp elucidated three new 19‐membered cyclohexadepsipeptides, namely tutuilamides A–C, featuring the unusual 3‐amino‐6‐hydroxy‐2‐piperidone and 2‐amino‐2‐butenoic acid residues (Keller et al., 2020). In 2022, Kang et al. reported the structure of epoxinnamide, a 31‐membered cyclodecadepsipeptide featuring four unusual amino acids (Kang et al., 2022), while Ohsawa et al. reported the total synthesis of decatransin, a 30‐membered macrocycle of 10 subunits featuring 2‐hydroxy‐5‐methylhexanoic acid (*HA*) and eight nonproteinogenic amino acids (Ohsawa et al., 2022).

**Figure 4:**
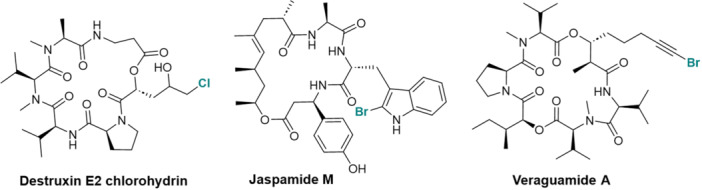
Structures of some halogenated CDPs: destruxin E2 chlorohydrin, jaspamide M and veraguamide A.

Other structural particularities have been demonstrated using NMR analysis. In particular, a number of halogenated CDPs have been isolated and identified: the chlorinated derivative destruxin E2 chlorohydrin (Figure 4) (Yeh et al., 1996), two brominated tryptophan modified jaspamide derivatives, Jaspamides M (Figure 4) and N (Gala et al., 2009), the alkynyl bromine functionalized veraguamides A (Figure 4), B, K, and L (Mevers et al., 2011), and the novel vinyl chloride‐containing CDP tutuilamides A–C elucidated in 2020 using a molecular networking strategy for isolation from cyanobacterium *Schizothrix* sp. (A and B) and *Coleofasciculus* sp. (C) (Keller et al., 2020). The HRMS data for these compounds, and the isotopic patterns characteristic of the aforementioned halogen atoms, were an important asset in validating the structures.

Additional special features were highlighted with NMR experiments. For instance, the 19‐membered cyclohexadepsipeptide pagoamide A displays two thiazole ring moieties (Li, Yu, et al., 2020), while the more complex macrocyle epoxinnamide contains an acyl chain of o‐1,2‐epoxypropyl cinnamic acid as well as an unusual bicyclic diaryl ether linkage (Kang et al., 2022). The particular structure of calcaripeptides A−C, 14‐ and 16‐membered cyclodidepsipeptides consisting of two amino acids (Pro and Phe) and a nonpeptidic substructure, was elucidated by 1D‐ and 2D‐NMR spectroscopy complemented by HRMS and X‐ray crystallography for chiral center configuration assignment (Silber et al., 2013). Feeding experiments with ^13^C‐labeled nutrients were also performed to investigate the biosynthetic origin of the CDP isolated from *Calcarisporium* sp. (Silber et al., 2013).

Advanced NMR analysis can also be useful in the revision of previously reported structure of CDPs. This is the case of isocereulide A, a complex 36‐membered cyclododecadepsipeptide and isoform of the food toxin cereulide isolated from the aerobic or facultative anaerobic bacterium *Bacillus cereus*, whose depsipeptide sequence has been updated by replacing the ^(L)^ ^O^Leu unit with an ^(L)^ ^O^Ile unit (Walser et al., 2021).

Finally, the importance of the choice of deuterated solvent for ^1^H and ^13^C NMR analysis was pointed out by Tripathi et al. in their identification study of the 26‐membered cycloheptadepsipeptides lagunamides A and B (Tripathi et al., 2010). Indeed, they demonstrated that some solvents may favor the coexistence of several conformers leading to complex NMR spectra with overlapping signals, while other solvents can favor one major conformer making spectra easier to interpret.

### 3.2 MS contribution to pure and sample mixtures

NMR is a technique that has proved to be essential for fully elucidating molecular structures. However, it requires micrograms of pure sample, which is a limitation, whereas the structure of compounds present in mixtures can in some cases be resolved by MS from few nanograms. However, this approach still represents a real challenge for unknown structure with some types of side chain (either peptidic or fatty acid). The formation of ionized molecular species and the production of a large and diverse population of fragment ions are necessary to reconstitute the original structure of the molecule and can be a “tour‐de‐force.” Ion fragmentation is greatly dependent on gas‐phase chemical reactivity, which remains difficult to predict and explain. Moreover, the ions in the gas phase are free of solvent, which considerably modifies their fragmentation. Thus, in the early stages, the interpretation of ion dissociations was mainly based on organic chemistry reactional mechanisms through a semi‐empirical approach unsubstantiated by quantum calculations. This is no longer the case, although the substrates must remain relatively simple. CDPs remain fairly complex and their specific conformations in the gas phase may have consequences on dissociation process orientation, which are difficult to model. The chosen ion polarity was mainly positive mode since the side chain of amino acids is predominantly alkyl groups rather than a deprotonatable functionalized side chain (i.e., Asp, Glu, His, Arg, Ser, and Thr^2^).

#### 3.2.1 Ionization and desorption methods

##### Gas phase ionization in vacuum and in AP conditions

The gas phase ionization modes (i.e., EI and CI) require an optimum volatility of weakly polar CDPs. This allows ionization from solvent‐free molecules either in vacuum (which requires heating the probe as soon as the sample is introduced) or at AP. On the other hand, to reach such volatility in polar molecules, it is necessary to derivatize polar functional groups at the side chain (basic or acidic residues such as arginine or aspartic and glutamic acid residues) of the intact molecule. The free amino acids provided by CDP hydrolysis are polar (even for the simplest amino acids) and require derivatization before analysis by gas chromatography coupled to MS (GC‐MS). Under AP conditions, gas phase ionization (e.g., atmospheric pressure chemical ionization, APCI) is possible either by direct introduction of the sample or after LC coupling to analyze the intact molecules present in a mixture. This ionization mode yields singly charged small‐size aggregates provided by ion/molecule reactions and AP solvation. In reduced pressure zone, aggregate desolvation yields singly charged molecular species generally as EE ions. In the case of very polar molecules, direct analysis without derivatization yields charged molecular species under desorption/ionization conditions, through production of singly or multiple charged aggregates. The desorption/ionization process can occur in vacuum (e.g., for CDPs, by FAB, not used anymore, and by MALDI) with natural and spontaneous aggregate desolvation in vacuum. Alternatively, at AP (i.e., ESI) where the desorbed single or multiplied charged large aggregates are desolvated after their acceleration in the reduced pressure zone (Kebarle & Verkerk, 2009). It results in a distribution of singly and multiply charged molecular species depending on (i) the use solvent (and additives), (ii) the desolvation conditions, and (iii) the size of the molecule and/or its composition in amino acid residues. In the case of CDPs, gas phase ionization and desorption/ionization under AP conditions will be particularly useful not only for determining molecular masses but also for obtaining structural information on CDPs' sequences by MS/MS.

###### “In vacuum” ion source

####### EI and the prompt cleavages of OE molecular ions of CDPs

During the 60's, ionization in the gas phase was exclusively based on EI wherein probe heating was used to make compounds sufficiently volatile. In this mode, a large internal energy (i.e., vibrational energy distributed over 15 electron volts, eV), is transferred, through the ionization step, into molecular ions (M^+•^) which promptly dissociate in the ion source due to the vibrational energy distributed at both the excited electronic levels and the ground level (Levsen, 1978). As molecular ions are OE systems, they dissociate by loss of either a neutral radical or molecule. To obtain the structural information needed from an EI mass spectrum, a special effort was made at that time to identify systematic mechanisms that could interpret the fragmentations of the M^+•^ ions (as OE species) (Budzikiewicz et al., 1964). They involve both homolytic and heterolytic cleavages through direct cleavages or rearrangements induced either by the site which carries the (+•) charge or by the radical site (•) after the M^+•^ isomerization into a distonic ion, the charged site being spectator of the resulting fragmentations. Such molecular ions are generated from synthetic or natural molecules of known structures or elucidated by other structural methods such as infrared (IR) or ultraviolet (UV) spectroscopy, circular dichroism (CD), but more routinely by NMR either from intact CDP molecules or after their hydrolysis. The interpretation of ion dissociations is mainly based on reactional mechanisms involving electronic effects, as used in organic chemistry. Note that the interpretation of prompt fragmentations was not detailed. However, to allow fragmentation comparison according to the ionization mode, we suggest fragment ion interpretation using the Roepstorff‐Fohlmann‐Biemann nomenclature (Biemann, 1988; Roepstorff & Fohlman, 1984).

**Figure 5:**
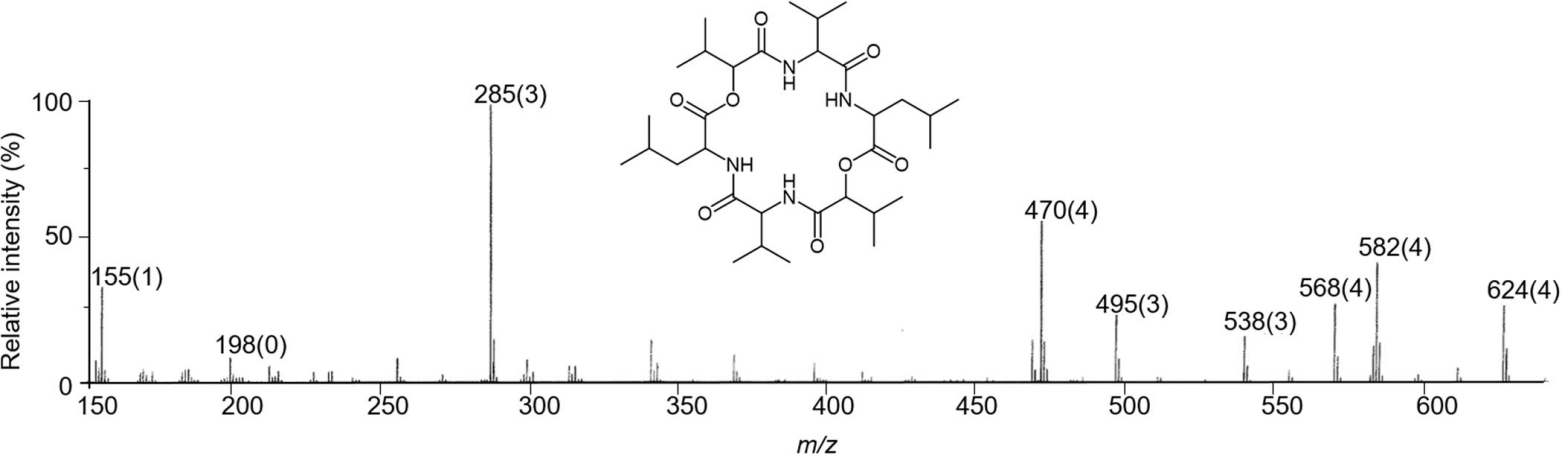
Electron ionization mass spectrum of sporidesmolide III, cyclo[^O^Val‐Val‐Leu]_2_ (Mw = 624 Da) using B sector instrument. In parentheses are noted the peak shifts related to the number of mobile protons in molecular and fragments ions from the EIMS of the d‐labeled sample obtained by H/D exchanges in solution with D_2_O. Figure adapted from Russell et al. (1964).

Wulfson et al. investigated early on the direct introduction EI mass spectra of 12‐ and 24‐membered CDPs (Wulfson et al., 1964). These EI mass spectra display the molecular ion and numerous product ions whose formation mechanisms are based on peptide bond cleavages, and/or hydrogen atom transfers and/or proton transfers from the M^+•^ ion. These H^+^/H^•^ transfers were interpreted using quasi‐concerted homolytic and heterolytic processes through six‐membered transition states in the case of sporidesmolide I (two ester linkages and one N‐methyl amide linkage with five Val residues and one Leu residue) and II (with one Val changed into Leu residue) from fungus *Pithowces chartarum* (Macdonald, Shannon & Taylor, 1964). Such mechanisms may be considered similar to the McLafferty rearrangement used for alkene losses from ionized ketones. These dissociation processes were used to rationalize the 42 and 56 Da losses from the Val et Leu residue side chains (Figure 5) of sporidesmolide III molecular ion (Russell et al., 1964) with a discussion of the presence of the residues with the D configuration.

An additional MS study has shown that sporidesmolide II structure is more complicated than it was first thought (Bertaud et al., 1965). The EI of various CDPs yields prompt “in‐source dissociations” from the molecular ion, for which improved interpretations based on homolytic bond cleavages have been reported (Wulfson et al., 1965). The influence of the peptide linkage (i.e., ester *vs*. amide bond) in the orientation of cleavage was not clarified in these studies. However, this effect is clearly described in the dissociation exploration of the molecular ion of cyclohexadepsipeptide antibiotics such as staphylomycin S (Kiryushkin et al., 1967). This compound consists of proline, threonine, N‐methylphenylalanine, and four nonribosomal amino acids: α‐aminobutyric acid, phenylglycine, 4‐oxopipecolic acid and picolinic acid. The latter is linked by an exocyclic amide bond to the amino group of threonine where its CO group is bonded by an amide linkage to the α‐aminobutyric acid residue. This ionized antibiotic decomposes through a first cleavage resulting in the CDP opening which takes place preferentially at the ester linkage (between the CO group of phenylglycine and side chain OH group of threonine). This can lead to the consecutive loss of residues by consecutive peptide bond cleavages. However, no evidence has yet been given that these processes are consecutive rather than competitive. Based on this work, Compernolle et al. (1972) considered the initial (CO_2_ + H^•^) formal loss during ring‐opening as the first step, despite low abundance. This release is commonly detected for the same family of CDP (i.e., staphylomycin S, S_2_, S_3_, dihydrostaphylomycin and its allo form). The CO_2_ neutral loss was previously observed by Macdonald and Shannon (1964) from the direct M^+•^ dissociation of angolide, with the cyclo[^(L)^ ^O^Val‐^(L)^Ile‐^(L)^ ^O^Val‐^(D)^allo‐Ile] sequence containing one ^(D)^allo‐Ile residue for (2 R,3 S)−2‐amino‐3‐methylpentanoic acid. In Compernolle's study (Compernolle et al., 1972) the detection of metastable ions (fragment ions spontaneously generated in the field‐free region (FFR) before entering the magnetic field of a sector mass spectrometer) demonstrated these consecutive residue losses (Table 3). The detection of this metastable peak linking a precursor ion to a fragment ion, was the premise of MS/MS for the structural analysis of CDPs (*vide infra*). The amino acid composition of the CDP isolated from *Fusaria* fungi (Carr et al., 1985) was reached after O‐trimethylsilane (OTMSi) derivatization, by GC‐EIMS (at 70 eV) and analyzed after separation with a chiral column to determinate the D/L configuration of each amino acid. The molecular mass of the intact CDP was determined through a combination of different gas phase ionization modes (i.e., EI, and/or CI with ammonia, CINH_3_), and confirmed by FABMS and NMR. The data from prompt fragmentations in EI mode were very useful for building the sequence containing [^(D)^allo‐Thr‐^(L)^Ala‐^‐(D)^Ala‐^(L)^Gln‐^(D)^Tyr‐^(L)^Leu], with a ^(D)^allo‐Thr for (2 R,3 R)−2‐amino‐3‐hydroxybutanoic acid. The presence of exocyclic side chain and endocyclic polar head of 3‐hydroxy‐4‐methyl‐tetradecanoic acid linked to N‐terminus of ^(D)^allo‐Thr and C‐terminus of ^(L)^Leu was demonstrated. More recently, beauverolides L and La were analyzed by HPLC (UV detection), NMR and GC‐EIMS (Jegorov et al., 1994). The presence of a hydroxy acid residue responsible for ester linkage was shown by GC‐EIMS posthydrolysis. The EI mass spectra of geodiamolides isolated from various tropical marine sponges (in particular, from *Cymbastela*) were interpreted based on the structure provided by NMR (Coleman et al., 1999).

**Table 3:** Main hgcyclodepsipeptides analyzed in electron ionization: (a) prompt M^+•^ dissociations and (b) neutral losses from metastable fragmentations.

Molecules	Origin	Function	Prompt fragment ions (diagnostic neutral loss)	References
Angolide	*Pithowces*	Antibiotic	(‐CO_2_)	Macdonald & Shannon (1964)
Beauverolides L and La	*Beauveria tenella* and *Paecilomyces fimosoroseus*	Insecticide	(‐CH_**3**_^•^, ‐CO_2_) **b, a** and various **a**_**1**_ immoniums	Jegorov et al. (1994)
di‐, tetra‐, hexa‐, octa‐, and dodecadepsipeptide	Not specified	Not specified	(‐CO_2_)	Wulfson et al. (1964)
Geodiamolides	*Cymbastela* sp.	Cytotoxic drug	no information	Coleman et al. (1999)
Sporidesmolides I and II	*Pithowces chartarwn*	Antibiotic	**b**	Macdonald, Shannon, and Taylor (1964)
Sporidesmolides III	*P. chartarwn*	Antibiotic	**b‐**(C_3_H_6_ **)** ^(*)^	Russell et al. (1964)
Staphylomycin S	*Streptomyces*	Antibiotic	**b**‐(CO_2_‐H)	Compernolle et al. (1972); Kiryushkin et al. (1967)

*Note*: (*) CO losses from metastable dissociations.

EIMS from a sufficiently volatile sample (or after a derivatization) was useful for resolving the CDP sequence from a possible ester bond cleavage to induce ring‐opening. However, amide bond cleavages may occur competitively and promptly in ion source to yield many fragment ions due to gas phase radical/cation properties. Ring‐opening results in the formation of distonic ions where the charge and radical are separated, being located at each end group. In addition, due to the molecular ion's high internal energy, it sometimes appears absent from the EI mass spectrum, thus complicating the structural interpretation.

####### CI and soft ionization of volatiles

In vacuum, CI based on proton exchange takes place between the gas phase sample and a protonated (or deprotonated) reagent through ion‐molecule reactions (Field, 1968). This promotes the exothermic formation of relatively stable protonated molecules [M + H]^+^ (or deprotonated molecules [M ‐ H]^‐^), as EE species, with low internal energy. This could be an alternative to EI which yields very reactive OE molecular ions (Table 3). Nevertheless, CI mode was frequently not used to analyze CDPs since the resulting EE molecular species have only been described by Crews et al. (1986). Using the marine sponge *Jaspin* Sp., they showed that the isolated jasplakinolide analyzed in CIMS using CH_4_ as a reagent gas yields both [M + H]^+^ and [M + C_2_H_5_]^+^ ions. In fact, the CI mode is used essentially for molecular weight determination in this case.

###### Atmospheric pressure ionization (API): APCI

Under APCI conditions, protonated (or deprotonated) molecules and adduct ions with for example ammonium (formate, acetate, and inorganic anions) are generated by ion‐molecule reactions. Their respective structures generally are hydrogen‐bonded systems. However, these reactions, which occur by electric discharge under AP conditions, generate together positive and negative singly charged aggregates (made up of solvents, organic salts, and analyte) that form the resulting plasma in the API source. From these aggregates, solvent‐free EE molecular species are produced by using gas phase desolvation based on weak energy collisions in the reduced pressure zone (or in funnels) in front of the mass analyzer. Although this mode is much less popular than electrospray (ESI, *vide infra*), it has been used for rapid LC‐MS screening of about 20 destruxins (Jegorov et al., 1998). In positive ion APCI, the “in‐source collision‐induced dissociation” (or “in‐source CID,” occurring in the reduced‐pressure zone by an increased desolvation voltage) could be used to almost fully sequence their respective structures. Unexpectedly, such sequencing was only partially achieved in MS/MS experiments under high collision energy (keV energy range) conditions using double sector instruments (Jegorov et al., 1998). Three CDP inhibitors of cancer cell growth were isolated. Their structures and stereochemistry were elucidated by NMR from compounds (kitastatine, respirantin with its valeryl derivate) provided from fermentation of *Kitasatospora* sp. Their respective molecular masses and elemental compositions were confirmed from [M + H]^+^ generated by APCI using HRMS (Pettit et al., 2009).

##### Desorption/ionization in vacuum and under AP conditions

###### Desorption/ionization in vacuum

In desorption methods, molecular species are mainly detected as protonated (or deprotonated) molecules and adduct ions with ammonium (or organic carboxylate or inorganic acid). In addition, alkali‐cationized molecules are mostly observed in desorption processes and are more abundant in positive mode than in negative ion detection mode. Only weak traces of the alkali metal cations involved are responsible for these cationized molecules (Table 4). This behavior of analytes submitted to desorption processes reflects the poor efficiency of gas phase ion production. The “in vacuum” desorption processes of polar compounds (thermally labile) were historically generated by FD (Beckey, 1977), a technique similar to field ionization (FI) mode (based on gaz phase ionization process of volatiles), the experimental difference being the use of (i) a filament with a sheath of carbon dendrites on which the sample is deposited and (ii) a heating filament under high field (resulting in the sample a field of few eV per Angström) which induces compound desorption (Röllgen, 1983). However, the desorption under chemical ionization (DCI), as thermal desorption, is preferred to FD mode since DCI can be installed on benchtop instruments such as quadrupole analyzers, using low ion acceleration voltages. The DCI mode was used to analyze geodiamolides from the *Cymbastela* sponge (Coleman et al., 1999) and the CDP antibiotic from *Fusaria* fungi (Carr et al., 1985) for confirming their molecular masses. In fact, the most popular desorption modes used under vacuum conditions are based on the particles impact on surfaces (heavy alkali metal ions or neutral gas) or by laser pulse (IR and UV) as described below.

**Table 4:** Main cyclodepsipeptides (with properties, characteristics and origin), and the desorbed molecular species by (a) SIMS, (b) FAB, and (c) MALDI with main product ions displayed in MIKE‐CID spectra (or in the B/E linked scan spectra) using multisector tandems.

Molecules	Origin	Function	source	Desorbed species	Prompt fragment ions	References
Serrawetin W2	*Serratia marcescens*	Surfactant	SIMS	[M − H]^−^, [M + H]^+^	‐	Matsuyama et al. (1992)
CDP complex	*Fusarium tricinctum*	Antifungal	FAB	[M + H]^+^	No information	Burmeister et al. (1985)
CDP‐glutathionyl conjugates	*Metarhizium anisopliae*	Metabolite of insecticide	FAB	[M − H]^−^	y	Loutelier et al. (1994)
Cereulide	*Bacillus cereus*	Toxin	FAB	[M + H]^+^, [M + H_3_O]^+^, [M + K]^+^ adding salt: [M + Rb]^+^, [M + Na]^+^, [M + Li]^+^, [M + Cs]^+^	No information	Suwan et al. (1995)
Destruxin analogs	Synthesis	Insecticide	FAB	[M + H]^+^	No information	Cavelier et al. (1997)
Destruxins	*Metarrhizium anisopliae*	Insecticide, antiviral, cytotoxic	FAB	[M − H]^−^	**y, c**	Lange et al. (1991)
GE3	Synthesis	Antitumor	FAB	[M + H]^+^, [M + Na]^+^, [M + K]^+^	No information ("in source fragmentations")	Hale and Lazarides (2002)
CDP	*Fusarium roseum*	Antibiotic	FAB	[M − H]^−^	**y**	Carr et al. (1985)
Serrawetin W2	*Serratia marcescens*	Surfactant	FAB	[M + H]^+^	**b, y**	Matsuyama et al. (1992)
Cereulide	*Bacillus cereus*	Toxin	LD	[M + K]^+^, [M + Na]^+^	**b**	Ducrest et al. (2019)
Cereulide	*Bacillus cereus*	Toxin	MALDI	[M + K]^+^, [M + Na]^+^, [M + NH_4_]^+^	‐	Mikkola et al. (1999)
Destruxins	*Metarhizium anisopliae*	Insecticide	MALDI	[M + H]^+^, [M + Na]^+^, [M + K]^+^	‐	Butt et al. (2009)
IB‐01212 analogs	*Clonostachys* sp.	Antitumor	MALDI	[M + H]^+^, [M + Na]^+^, [M + K]^+^	‐	Cruz et al. (2007)
Surfactins (C12, C13, C14, C15)	*Bacillus subtilis*	Antibiotic	MALDI	[M + H]^+^, [M + Na]^+^, [M + K]^+^	‐	Clark et al. (2018)

####### Secondary ion mass spectrometry (SIMS)

SIMS was developed by Benninghoven et al. (1976). EE molecular species are desorbed and accelerated for analysis by magnetic field scanning. This mode was used to study serrawettin W2. This CDP, partially made up of a serine, a threonine, and hydroxy fatty acid, was desorbed (*vide supra*) by SIMS. In positive and negative ionization modes, serrawettin W2 respectively yields an abundant protonated [M + H]^+^ and deprotonated [M − H]^–^ molecules which shifts by +18 *m/z* after alkaline hydrolysis. If it is followed by methylation of the generated carboxylic acid, then [M − H]^–^ shifts by +32 *m/z* (Matsuyama et al., 1992).

####### FAB

**Figure 6:**
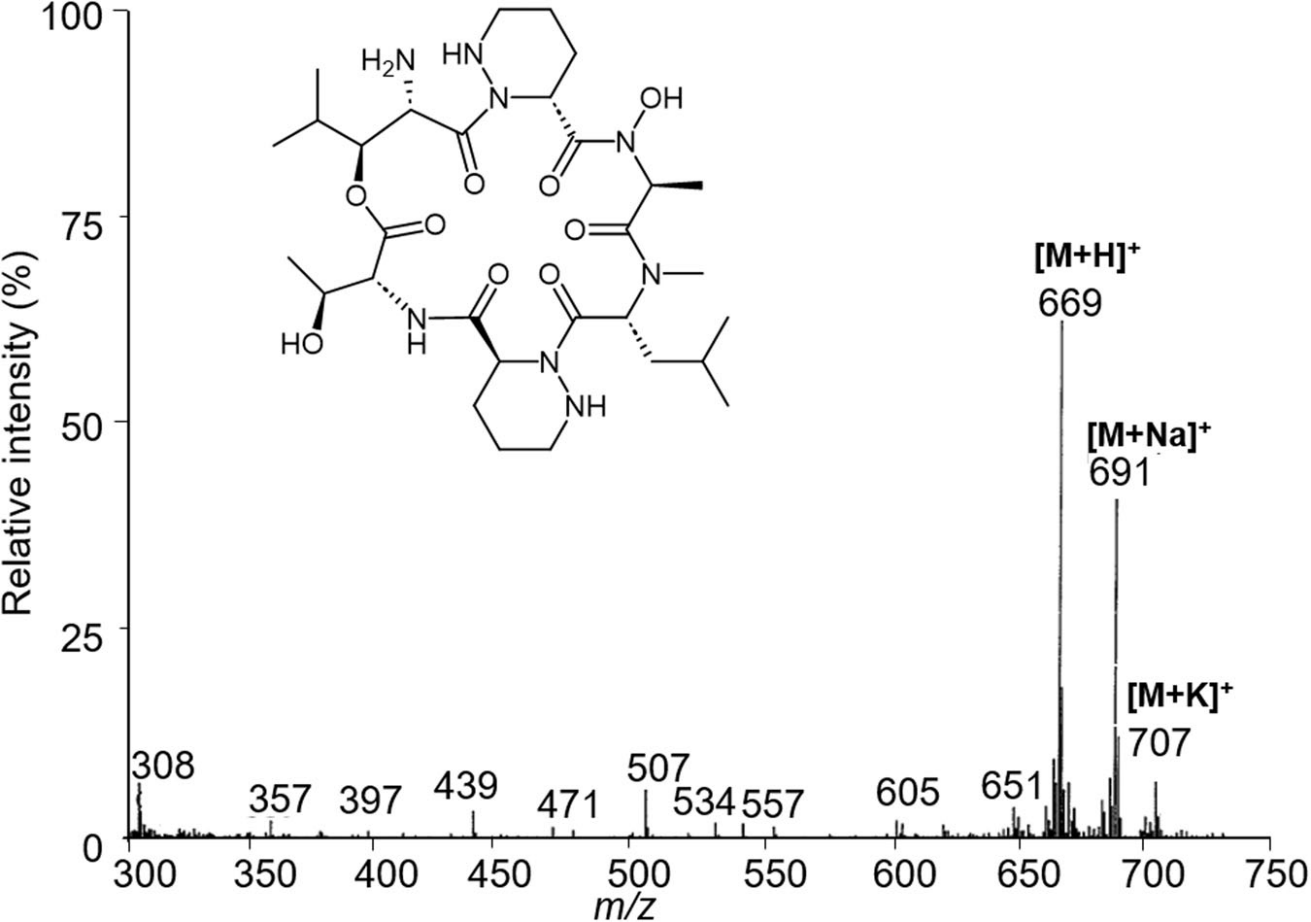
Positive ion fast atom bombardment mass spectrum of the synthetic GE3 cyclodepsipeptide using reversed geometry B‐E sector instrument. Ion abundances are relative to the base peak *m/z* 179 (nonreported). Figure adapted from Hale and Lazarides (2002).

This “in vacuum” desorption mode was introduced by Barber et al. as an alternative to the SIMS mode, using a liquid matrix (Morris et al., 1981). Under FAB desorption conditions, a high kinetic energy (KE) neutral beam (e.g., Xe, Ar, or Cs) impacts a target surface as in SIMS. However, in FAB mode, the sample is dispersed as a suspension in the liquid matrix such as mainly glycerol, thio‐glycerol, or 4‐nitrobenzyl alcohol. The impact of the high KE primary neutral beam leads to desorption of weakly charged aggregates that are quickly desolvated in these vacuum conditions. In Carr's study of an antibiotic from *Fusaria* fungi, MS/MS was developed using FAB to desorb abundant [M − H]^–^ main beam to be activated in the collision cell (Carr et al., 1985). It was shown that this antibiotic results from the cyclization of the linear peptide into a macro‐lactone ring generated by the interaction between the OH group of, for instance, a hydroxy fatty acid and a carboxylic acid at the C‐terminus of the peptide *(vide infra)*. Burmeister and co‐authors studied a CDP mixture produced from white corn grits fermented with *Fusarium tricinctum* NRRL 3510 (Burmeister et al., 1985). The resulting species were characterized by their FAB mass spectra, combined with determination of the amino acid composition, indicating the formation of three CDPs, one of which was predominantly produced. From FAB experiments on destruxins (Lange et al., 1991), prompt “in‐source dissociations” (or prompt cleavages) provided information about the structure. The sequence of CDP‐glutathionyl conjugates was achieved using a linked B/E scan in a double sector instrument with a FAB negative ion beam (Loutelier et al., 1994). To verify the stepwise synthesis of three destruxins, the FAB source was very useful for measuring the molecular mass of synthetized products since the [M + H]^+^ ion was efficiently desorbed (Cavelier et al., 1997). The 12 intermediates of the total stepwise synthesis of the antitumor agent GE3 (Hale & Lazarides, 2002), a 19‐membered cyclohexadepsipeptide, were characterized by IR spectroscopy, NMR and FAB MS (HRMS measurement from [M + H]^+^, *m/z* 669.3936, i.e., elemental composition C_30_H_53_N_8_O_9_). The respective molecular masses of the different synthetic intermediates were determined from the [M + H]^+^ or [M + Na]^+^ ions displayed in the FAB mass spectra. Low abundance [M + K]^+^ ions are also displayed in FAB mass spectra (Figure 6).

However, it is not possible to predict which molecular species is the most abundant when in solution. In the case of cereulide, an ionophore of K^+^, the FAB mass spectrum indiscriminately displays the alkali‐cationized molecule (Suwan et al., 1995). This alkali‐cationized molecule distribution depends on the experimental source conditions and the sample in a matrix. This behavior towards alkali metal cations, which limits any consideration on cation selectivity of CDPs, is the same with other desorption/ionization processes *(vide infra).*

####### Matrix‐assisted laser desorption ionization (MALDI)

Early after the development of infrared laser desorption (IRLD) (Tabet & Cotter, 1984), MALDI was introduced in the 80 s by Karas et al. (1985) and is based on the use of UV laser (N_2_, 337 nm, pulse duration of 4 ns). The laser radiation impacts a target surface on which the sample is dispersed in a matrix (m) and left to dry (Vorm et al., 1994). Different acid matrices are available, the most popular being ferulic, sinapinic, α‐cyano‐4‐hydroxycinnamic and 2,5‐dihydroxybenzoic acids. The choice of matrix depends on the compounds to be analyzed. It is noteworthy that the internal energy of desorbed ions depends on matrice. For instance, small proteins with α‐cyano‐4‐hydroxycinnamic acid are characterized by broader signals (due to a significant contribution of metastable dissociations in flight) than those provided with sinapinic acid yieding narrower signal due to larger contribution of prompt ion formation in source.

**Figure 7:**
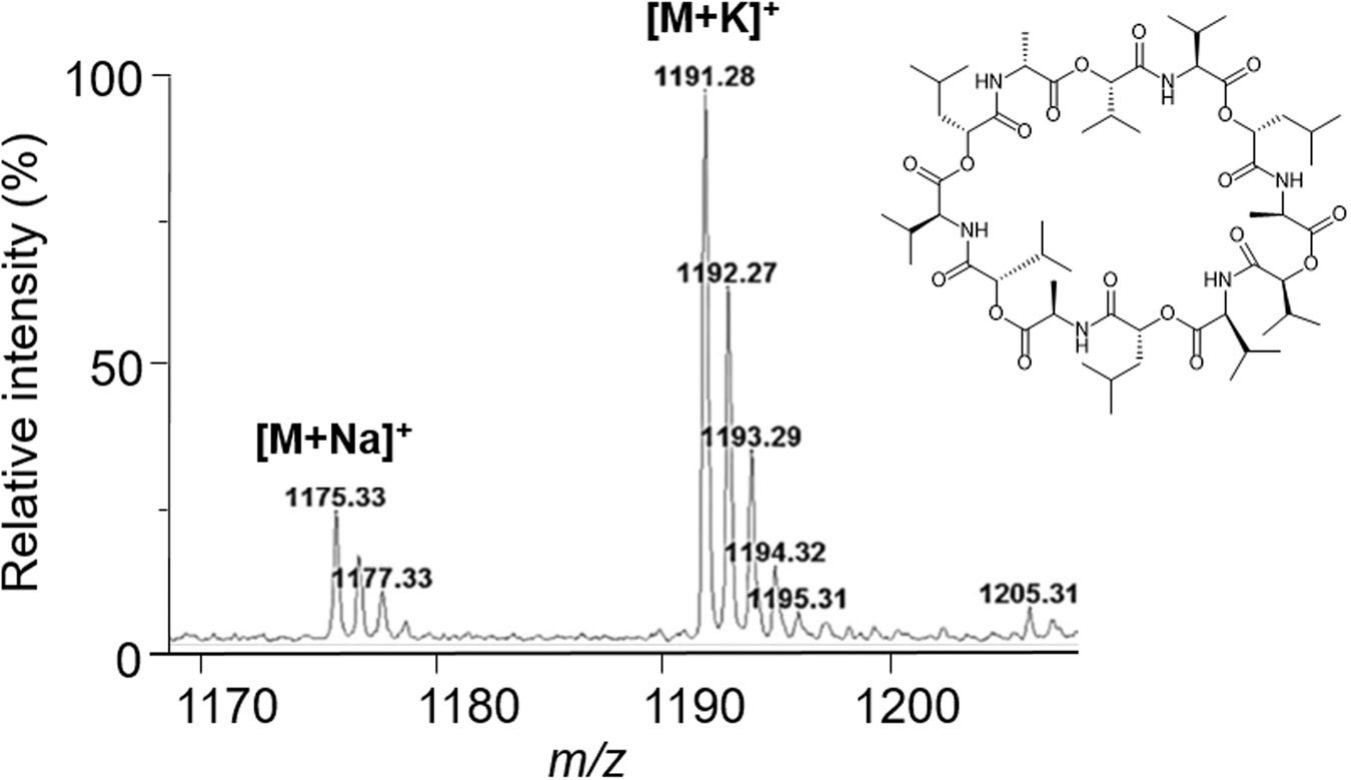
Zoom on the desorbed molecular species of LDUV‐TOF mass spectrum of cereulide extract from *Bacillus cereus* strain MB8. Figure adapted from Ducrest et al. (2019).

The matrix absorbs the laser's radiation energy and acts as a protective envelope for thermolabile samples. From aggregates consisting of many matrix molecules (m) in excited electronic states (m*), molecular ions (m^+•^) are first formed from this matrix by cooperativity. Next comes the formation of mH^+^ by hydrogen (or proton) transfer within the aggregate. At the same time, as the aggregates decrease in size by natural desolvation in a vacuum, the small aggregates that result produce [M + H]^+^ due to proton transfer (M + mH^+^ ‐> m + [M + H]^+^) (Fournier et al., 2002; 2005; Karas et al., 2000). This description is based on the “lucky survivor ions” model (Karas et al., 2000). Although this desorption mode has not been extensively used for CDP analysis, it is useful for rapid molecular mass determination. Cereulide from *Bacillus cereus* is found in soil and food. This poses serious concerns from the point of view of health and food safety (Dierick et al., 2005; Naranjo et al., 2011; Takabe & Oya, 1976; Thery et al., 2022; Yang et al., 2023). The MALDI of cereulide yields mainly potassium‐cationized molecules significantly larger than the sodiated molecules and ammonium adduct of cereulide (Mikkola et al., 1999). However, other experiments have shown that the sodiated molecule was the most abundant cationized molecule. Due to its toxicity, it is necessary to detect it quickly and simply without any special processing of the sample. Thus, a faster procedure using laser desorption (UVLD, with 200 Hz Nd:YAG and N_2_ lasers), requiring no matrix addition thanks to the ionophoretic properties of cereulide was also implemented with MALDI. Desorption of this toxin gives rise to the formation of alkali‐cationized molecules (Figure 7). The detection limit was 30 ng/mL in MALDI and 30 pg/mL in LD. Thus, this approach was successfully tested to detect this toxin in samples of milk, rice, ravioli and mashed potato (Ducrest et al., 2019). The [M + K]^+^ ion is the main species detected here and allows cereulide identification. This could be attributed to the ionophorous character of cereulide, which is specific to potassium in solution. However, the distribution of adduct ions with alkali metal cations depends on experimental conditions. Thus, it would be inappropriate to consider that in the gas phase, the behavior of cereulide towards potassium cation could prove that this toxin is an ionophore of K^+^ as it is in solution. However, a nonlinear ion abundance evolution is observed depending on concentration.

**Figure 8:**
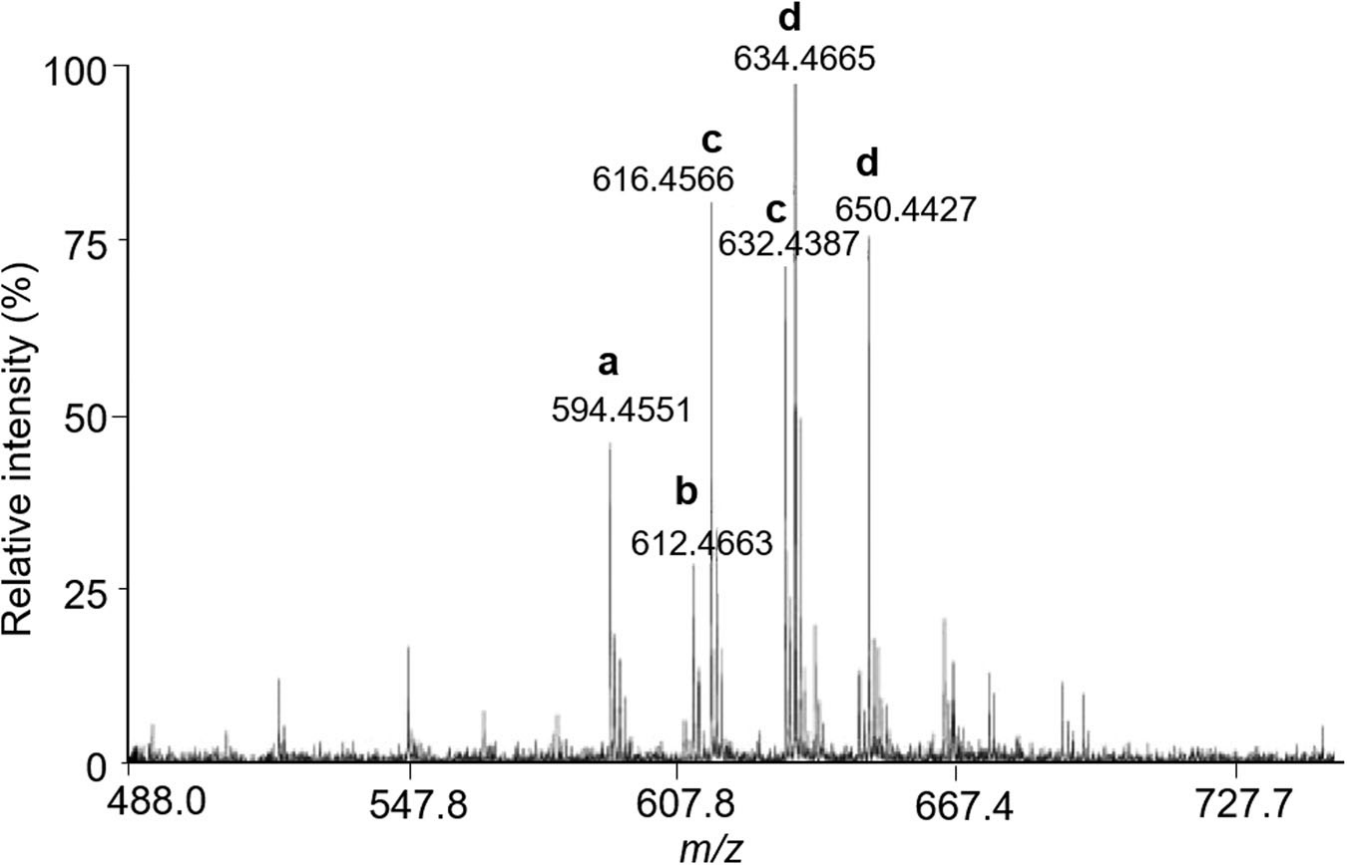
Zoom on the desorbed molecular species of MALDI‐TOF mass spectrum of the destruxin (dtx) mixture: (a) the protonated isobaric E and/or B dtxs at *m/z* 594, (b) the dtx E/B‐diol (*m/z* 612), (c) the alkali‐cationized E/B dtxs species (*m/z* 616 and *m/z* 632), and (d) the alkali‐cationized E/B‐diol dtxs species (*m/z* 634 and *m/z* 650), from a culture extracted from *Metarhizium anisopliae*. Figure adapted from Butt et al. (2009).

The structures of the cytotoxic Marine IB‐01212 (Cruz et al., 2006) have been analyzed by LC‐ESIMS (*vide infra*) and their synthesized analogs were analyzed by MALDI (Cruz et al., 2007). MALDI‐TOF‐MS was used to analyze the destruxins after purification from a culture of *Metarhizium anisopliae* (Figure 8). The isoform E or B species or their diol forms were only detected as [M + H]^+^ species with their alkali‐cationized molecules (Butt et al., 2009).

###### Desorption/ionization under AP conditions: Electrospray (ESI)

The electrospray desorption/ionization (ESI) mode is based on the formation of sprayed charged droplets from a solution subjected to a high electric field (i.e., from 2 to 5 kV depending on many different source and solution parameters) under AP conditions (Yamashita & Fenn, 1984). The evaporation rate of these droplets depends on the source temperature. This results in rapid, consecutive Coulomb explosions that produce charged droplets of decreasing size (Kebarle & Verkerk, 2009). Depending on the size and charge number of these small droplets, desorbed aggregates carrying many charges are desorbed, desolvated and focalized in a reduced‐pressure zone (such as a funnel, S‐lens or skimmer device). The solvent‐free molecular species may be charged by one charge or by charge distribution depending on the source conditions (temperature, solvent, presence of salts, etc.), desolvation conditions (the number of charges is reduced by the increase in desolvation potential according to the size of the aggregates introduced), capillary temperature, analyte size and composition, gas phase properties of the analytes (e.g., unfolded or folded conformations, acidity and basicity of functional groups) (Kebarle & Verkerk, 2009). Following desorption processes, the presence of alkali‐cationized molecules with one cation is frequently observed in the ESI mass spectra in positive mode, which is not the case in APCI since only gas phase ionization takes place. Their observation (without any cation being introduced) can be enhanced, for example, by (i) a higher desolvation voltage, and/or (ii) increasing the analyte's concentration (Darii et al., 2017). One or more alkali metal ions can cationize the molecule when two or more acidic sites are contained in the analyte, and in this case, alkali‐cationized deprotonated molecules (i.e., *deprotonated salt*) can be displayed in ESI mass spectra in negative mode. On the other hand, to limit cationized molecules, ammonium carbonate (or carboxylate) can be introduced and consequently, the contribution of protonated (or deprotonated) molecules is increased (Colsch et al., 2017). ESI mode is particularly suitable for LC coupling (Arpino, 1992), which quickly became popular and is mainly used for mixture analysis.

**Figure 9:**
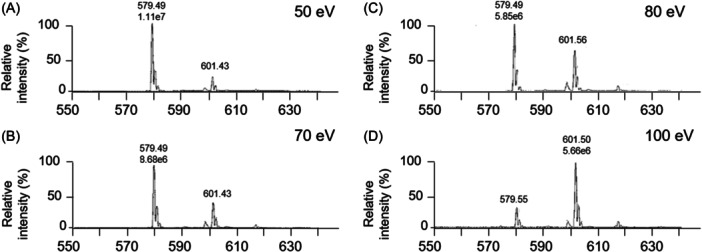
Zoom on the desorbed molecular species (*m/z* 579,49, *m/z* 601.43, and *m/z* 617.46 corresponding to [M + H]^+^, [M + Na]^+^, and [M + K]^+^, respectively) of ESI‐TQ mass spectra of cyclopeptide cyclo[Pro‐Ile‐NMeVal‐NMe‐Ala‐βAla‐^O^Ala] depending on the desolvation voltage applied: (A) 50 eV, (B) 70 eV, (C) 80 eV, and (D) 100 eV. Figure adapted from Cavelier et al. (1999).

In a comparative study between cyclopeptides (e.g., cyclo[Pro‐Ile‐NMeVal‐NMe‐Ala‐ßAla‐NorVal], NMe as N‐methyl and ßAla as 3‐aminopropanoic acid and NorVal as 2‐aminopentanoic acid) and CDPs such as destruxins (e.g., cyclo[Pro‐Ile‐NMeVal‐NMeAla‐ßAla‐^O^Ala]), Cavelier et al. showed the effects of an increase in desolvation voltage on the distribution of desorbed molecular species (Figure 9), where the resulting alkali‐cationized forms became more abundant (Cavelier et al., 1999).

It is important to optimize ESI source conditions, especially in LC‐MS coupling, to achieve better sensitivity. This is particularly true for the mass measurements of such compounds (Table 5). Detection and identification of destruxins A, B, B2, D, and E (plus their E diol, dihydro A and N‐di‐methyl B homologues) in a crude extract of the fungus *Metarrhizium anisopliae* were achieved in LC‐ESI‐HRMS from the accurate [M + H]^+^ and [M + Na]^+^ mass measurements (Potterat et al., 2000). A systematic study based on the HPLC–DAD (diode array dectector) system coupling to an ion trap mass spectrometer using sequential MS^n^ experiments and retention times allowed identification of most destruxins (Seger et al., 2004). Similarly, new compounds such as HA23 were detected in a fungal isolate of *Fusarium* sp. with an exocyclic fatty acid chain (Feng et al., 2002). Complex glycopeptides derived from the antibiotic vancomycin and consisting of sugar amino acids (or sugar amino alcohols, SAA) were synthesized (Bughin et al., 2007) and their molecular masses confirmed from both the [M + H]^+^ and [M + Na]^+^ ions by ESI under high‐resolution conditions (HRMS). The same approach was used to determine the molecular mass of calcaripeptides A−C produced from a *Calcarisporium* strain (Silber et al., 2013). The structures of apratoxin S4 and its epimer, in addition to two analogs, were investigated and their synthesis optimized. The molecular weights of synthetic intermediates and of apratoxin were confirmed from their sodiated molecular ions (Chen et al., 2014). Antifungal lipo‐CDPs such as alveolarides A, B, and C consisting of endocyclic 2,3‐dihydroxy‐4‐methyltetradecanoic acid and a β‐phenylalanine residue, extracted from *Microascus alveolaris* strain PF1466 were mainly identified by NMR and their molecular mass determined by ESI‐HRMS using their sodiated molecules (Fotso et al., 2018). HRMS was mainly performed using a Qq/TOF hybrid instrument. However, Fourier transform (FT) instruments were also used. The molecular masses of different veraguamides (Mevers et al., 2011), produced by a cyanobacterium from shallow water off the Pacific coast of Panama, were determined by low resolution instruments with an ion trap (IT) and HRMS with an FT analyzer (i.e., Orbitrap analyzer). The molecular masses of the CDPs skyllamycin A and B, as well as other forms (extracted from *Streptomyces* sp.), were determined by ESI‐HRMS (Pohle et al., 2011). Abundant beauverolides produced by fungi and used as biopesticides are biologically active substrates and were analyzed by LC‐MS instruments using ion trap and Orbitrap analyzers. The accurate molecular mass was measured from the [M + H]^+^ ions mainly desorbed under ESI conditions (Šimčíková et al., 2020).

**Table 5:** Main cyclodepsipeptides desorbed by ESI or ionized by APCI, studied in low energy collision: (a) properties, characteristic and origin, (b) desorbed or ionized species, (c) main product ions displayed in product ion spectra recorded under low energy collision conditions using (i) resonant with IT (low resolution) and for very high‐resolution tandem with FT/ICR or Orbitrap and (ii) nonresonant excitation modes with tandem QqX with X = Q, IT mass filter (low resolution), X = TOF (high resolution), and X = FT analyzer (ion‐cyclotron resonance, ICR and orbitrap allowing very high resolution).

Molecules	Origin	Function	Source	Ionized species	Prompt fragment ions	References
Alveolarides A,B,C	*Microascus alveolaris*	Antifungal	ESI	[M + H]^+^, [M + Na]^+^, [2 M + Na]^+^	‐	Fotso et al. (2018)
Apratoxin S4	Synthesis	Antitumor	ESI	[M + Na]^+^	‐	Chen et al. (2014)
Beauverolides	*Isaria* *fumosorosea*	Insecticide	ESI	[M + H]^+^	‐	Šimčíková et al., 2020
Calcaripeptides A‐C	*Calcarisporium* sp.	Antifungal	ESI	[M + H]^+^	‐	Silber et al. (2013)
Destruxins	Synthesis	Insecticide	ESI	[M + Na]^+^, [M + K]^+^, [M + K + H]^2+^	**b, a**	Cavelier et al. (1999)
Destruxins	*Metarhizium anisopliae*	Insecticide	ESI	[M + H]^+^, [M + Na]^+^	‐	Potterat et al. (2000)
Dextruxins	*Metarhizium anisopliae* and *Trichothecium roseum*	Insecticide, antiviral, cytotoxic	APCI	[M + H]^+^	**b, a, (a‐H**_**2**_ **O), and a**_**1**_ **immoniums**	Jegorov et al. (1998)
GE3	Synthesis	Antitumor	ESI	[M + H]^+^, [M + Na]^+^	‐	Hale and Lazarides (2002)
HA23	*Fusarium* sp.	Antifungal	ESI	[M + H]^+^, [M + Na]^+^	‐	Feng et al. (2002)
Kitastatin 1, respirantin and valeryl respirantin homologue	*Kitasatospora* sp	Antitumor	APCI	[M + H]^+^	**‐**	Pettit et al. (2007)
Sugar AA‐CDP	Synthesis	Antifungal	ESI	[M + H]^+^, [M + Na]^+^	‐	Bughin et al. (2007)
Skyllamycin A and B	*Streptomyces* sp.	Antitumor	ESI	[M + H]^+^	**b, y**	Pohle et al. (2011)
Veraguamide	*Oscillatoria margaritifera*	Antitumor	ESI	[M + H]^+^, [M + Na]^+^, [M + K]^+^	‐	Mevers et al. (2011)

#### 3.2.2 MS/MS: Collisional ion activation in resonant (MS^n^) and nonresonant (MS/MS) conditions

The tandem instrumentation used for the CDP analysis is reported below and this description is not exhaustive (e.g., tandem instruments involving ion‐mobility are not yet used for such a structural purpose). There are several commercial systems for activating ion dissociation. Activation of simply charged ions can be achieved using a variety of techniques, which can be commercially installed on tandem spectrometers after the selection zone for the ions to be excited. These involve either laser radiation (Infrared Multi‐Photon Dissociation, IRMPD; Little et al., 1994; Macias et al., 2020) or collisional processes with (i) electrons (electron‐induced dissociation, EID; Cody & Freiser, 1979; Gord et al., 1993), (ii) metastable atoms (Metastable atom induced dissociation, MAD; Berkout, 2006; 2009; Cook et al., 2009), (iii) a surface (surface‐induced dissociation, SID; Snyder et al., 2022), and finally (iv) gaseous targets (collision‐induced dissociation, CID, *vide infra*). In ionized CDPs, the IRMPD and EID modes have been used solely to explore their analytical potential, which is not without interest. In contrast, the most common activation studies for ionized CDPs have been carried out using CID.

##### Collisional activation of selected precursor ion

For singly charged molecules (case of CDPs) selected in the first analyzer, collisional ion activation (i.e., CID) is thus the most popular since it can be applied independently of the mass spectrometer used. The collisional processes with gaseous target are mainly applied to activate dissociations of the selected singly (multiply) charged molecules. Two collisional regimes are possible: (i) several keV collision energy regimes were first developed using multisector instruments where the target is helium (He) but today, such tandems are not used except in TOF/TOF instruments (*vide infra*). Under these KE conditions, the internal energy reached by selected precursor ion is mainly based on the vibrational energy distributed in the ground level and excited electronic levels which results in instrument‐independent collisional spectra^3^ although the abundance of survivor precursor ions is 10^2^ to 10^3^ greater than the most abundant product ions and (ii) eV collision energy range, in radio frequency (RF) only multipole cell where the target is argon (Ar) or nitrogen (N_2_), results in internal energy based on vibrational energy essentially distributed in the ground level of precursor ion. Note that beyond 150–200 eV for small size ions (domain of molecules lower than 500 Da), collisional efficiency dramatically decreases. In ion trap device, numerous low energy collisions take place with He (or nitrogen) as target/buffer gas and the internal energy of activated ions depends on excitation RF voltage amplitude and its duration. Consequently, the recorded product ion spectra are instrument‐dependent as in ion beam experiments under the eV KE range conditions. Collision processes can also occur on metallic target (i.e., SID) (Cole et al., 1992; Mabud et al., 1985; Snyder et al., 2022) but were not yet used to analyze CDPs. Two mainly low energy collisional ion excitation modes for precursor ion activation can be used with gaseous target:

(I) **Nonresonant mode** based essentially on *ion beam* experiments where the collision process takes place in multipolar RF only cell (quadrupolar or hexapolar device) in which both the precursor and resulting product ion are transferred to the second analyzer (quadrupole, TOF, and both the orbitrap and ion cyclotron resonance [ICR] ion trap cells). For *ion storage experiments,* this excitation mode is possible by using the potential applied at the end caps (or gates) on the axis of the linear ion trap (LIT) where all ion manipulation steps can be sequential in a common zone according to the device system. First, “in axis collision,” like ion beam collision (higher‐collision energy dissociation, HCD) (Olsen et al., 2007), is a nonresonant process and secondly, the product ion analysis step may occur in the same zone by ion ejection (*vide infra*). In nonresonant mode, the collisional step is much faster than that of the dissociation rate constant. The collisional activation process is generally considered to be *ergodic*: there is a spontaneous randomization of the internal energy on all bonds (ergodicity principle) before the cleavage of the weakest bond (independently on the impacted site of ions by the gaseous target). In both *ion beam* and *ion storage* experiments, the main properties characterizing the nonresonant mode are as follows: the precursor ion promptly activated by one or a few collisions (until 10 max) in a relatively short duration (from few µs to about a hundred µs). This process yields first generation of product ions and their consecutive dissociations, due to collision activation of product ions in the collision cell; and
(II) **Resonant mode** based on ion storage experiments in ion trap (IT), where a very large number of thermal‐like collisions occur (in contrast to the nonresonant process) for the ion cloud KE relaxation with the buffer gas or to activate selected precursor ion. This device allows ion storage for several tens or hundreds of msec to manipulate ions through several steps required for ion injection, ion relaxation, ion selection, ion excitation, and ion ejection for detection. As soon as the precursor ion is selected and relaxed, it is resonantly excited by applying its motion frequency. The amplitude of this frequency depends on excitation energy to be transferred, taking care that it is not too high since the ion cloud risks being ejected from the IT. The product ions emerging after a very large collision number during about 10 ms, are relaxed and not excited since their respective motion frequency varies to that of their precursor. Consequently, consecutive decompositions do not occur unless the residual internal energy is enough for subsequent fragmentations, as well as for exothermic consecutive processes (Tabet et al., 2021), and the various product ions are due to competitive dissociations of selected precursor. Both the survivor precursor and product ion detection can occur either by “in axis ion ejection” (nonresonant mode) or by radial ion ejection (resonant mode) from the IT cell. However, the IT advantage concerns its possibility to perform sequential MS^*n*^ experiments allowing selection of a product ion of the first generation to reach those of the second generation and so on.

Among the few instrumental limitations encountered in MS/MS, one concerns the recording of product ion spectra with a full *m/z* scale down to the low *m/z* values. Indeed, various reasons make discrimination take place for the detection of low *m/z* product ions:

(I) **under nonresonant conditions**, poor transmission of low *m/z* ions formed under the highest collision energy conditions due to scattering effect occurring in ion beam experiments and, to a lesser extent in LIT due to the kinetic energy relaxation of product ions after their formation prior to be analyzed by “in axis” ion ejection and
(II) **under resonant excitation conditions** of precursor ion, the *m/z* values of the product ion cannot be less than a fraction of the *m/z* values of the precursor ion, depending on the multipolar field used (hexapolar at *q_z_* = 0.78 or quadrupolar at *q_z_* = 0.908) for the selective ion ejection and detection. So, for a default value of *q_z_* = 0.25 to resonantly excite the precursor ions, the lowest *m/z* value of the stored and detected product ions is approximately either a 1/3 or a 1/4 of the precursor ion *m/z* occurring at a resonance either hexapolar or quadrupolar for the selective ion ejection scan, respectively. This product ion storage and detection limit is the low‐mass‐cut‐off (LMCO). This limitation can be counterbalanced by suitable sequential MS^*n*^ experiments (*vide supra*). In addition, independently of the activation mode in FTMS analyzer (Orbitrap or ICR cell), the use of FT signal treatment does not allow detection of product ions of *m/z* ratio lower than 50 *m/z* due to the very high motion frequencies which need a lot of computer memory.

Collisional excitation processes in resonant mode essentially result in competitive processes as precursor ions are exclusively activated since product ions have higher frequencies than the precursor (Darii et al., 2021). Whereas only the nonresonant excitation leads to consecutive processes as both the precursors and resulting product ions are submitted to collisions. This nonresonant mode yields the greatest sequence overlap. In resonant mode, the same information may be obtained using sequential MS^n^ experiments. However, possible risk of sequence isomerization of the product ions during the ion manipulation (e.g., the isolation step, ion cloud relaxation and excitation) is possible and hence, ambiguity in the sequence order can result (Hart et al., 1992).

##### Ion beam tandem and ion storage instruments

###### Multisector devices and keV collision energy

Since the 1960s, organic and natural product molecules have been ionized in vacuum by EI to generate molecular ions with a broad internal energy distribution (through ~15 eV). The most excited molecular ions lead to prompt fragmentations in the source (Table 3), and those with lower energies can dissociate after acceleration, along their trajectory according to their internal energy to be analyzed by a single (or double) sector instrument and then reach the detector. The prompt fragment ions appear as narrow‐resolved signals at *m*/*z* related to the ion masses while the latter appear as a diffused broad signal having the shape of a “molehill”. These fragment ions are called “metastable” for a transition M^+•^→ m^+^ + n^•^ where m^+^ is noted m*. The fragment ions detected are those formed just after the source, in the 1st free field region (FFR1) of a single sector (magnetic field with the B scanning) or double sector instrument (electric field E and magnetic field B, E/B configuration, with E = E_o_ and B scan for mass spectrum recording). This m* product ion was detected in the mass spectrum as a unresolved signal with the value m* = m^2^/M not always easily detected with precision (Table 3). This detection approach on commercial instrumentation was the premise of MS/MS in the 70's.

From the late 70's to the late 90's, the double sector instrument with reversed geometry (B–E) were used for MS/MS experiments to perform precursor ion mass (M) selection (called “mass analyzed”) with the fixed B_M_ field value to be submitted to high energy collision regime (CID) beyond several keV in the 2nd FFR in front of the E sector. The E field is scanned from (E_o_ to 0, E_o_ for filtering only ions directly formed in source) to separate all resulting product ions through their respective KE related to the mE_o_/M ratio, E_o_ for precursor ion detection (ion kinetic energy or IKE separation step). The recorded spectra are called MIKE (mass‐analyzed‐ion kinetic energy) and MIKE‐CID with collision activation of ions.

**Figure 10:**
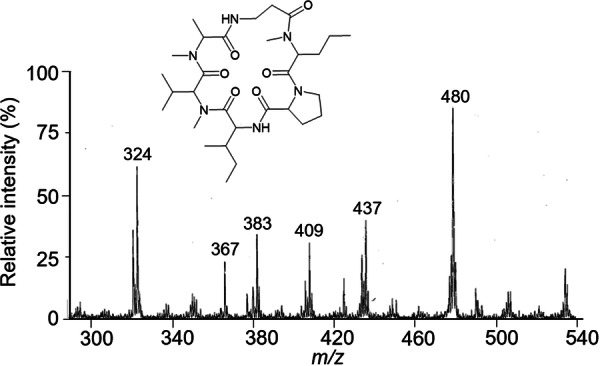
Product ion spectrum of protonated system *m/z* 593 (as base peak, nonreported) desorbed in fast atom bombardment using B/E linked scan mode, double sector mass spectrometer. Figure adapted from Cavelier et al. (1999).

Serrawettin W2, a surfactant exolipid (3‐hydroxydecanoic acid, 3OH‐C_9_COOH) produced by *Serratia marcescens*, was first studied (Matsuyama et al., 1992), by negative ion FAB before and after its hydrolysis (*m/z* shifted by +18 *m/z*) followed by methylation of the resulting C‐terminal site (*m/z* shifted by +14 *m/z*). The presence of five residues is shown to be linked to the exolipid. MIKE‐CID on the resulting ester after protonation allows us to give the sequence as (OH‐C_10_CO‐Leu‐Ser‐Thr‐Phe‐Ile‐COOCH_3_) using both the **b** and **y** product ion series according to the Roepstorff‐Fohlmann‐Biemann nomenclature (Biemann, 1988; Roepstorff & Fohlman, 1984). From this study performed using a reversed geometry double sector B–E instrument, it appears that the resolution of the signals in MIKE‐CID was poor due to the KE release occurring during the dissociation processes. The use of the B/E linked scan using E/B double sector instrument allowed to avoid the effect of KE release which results in broadening signal. Thus, it improves the signal resolution allowing the separation of m and (m + 1) peaks. This mode was used to study fragmentation of protonated destruxins (DTX‐A and DTX‐B) from *Meturhizium anisopliae* (a fungi) and synthetic Lac‐6 DTX‐E (with an ester linkage with endocyclic lactic acid [Lac] as ^O^Ala) and its analogues (Hpy‐6 DTX‐E, Hpp‐6 DTX‐E, and Hpy(TMS)‐6 DTX‐E with the methyl side chain substituted by ethynyl, phenyl, and trimethyl silyl ethynyl groups, respectively) (Loutelier et al., 1995). These precursor ions were desorbed by FAB using 6 keV neutral Xe beam and glycerol as matrix target. Their fragmentations are discussed later (see §3.2.2.3). From the ring‐opening at the methylated amide linkage, the classic **b** series *(vide infra)* can be provided until b_2_. Furthermore, an additional series starting with the formal loss of the neutral N‐methyl dipeptide was unexpectedly provided resulting in a new **b** series of internal fragment ions with an N‐acyl group as the terminal group. These dissociations are described as charge‐remote processes in which excited [M + H]^+^ electronic states are involved in such reactions (not discussed further here). However, charge‐promoted processes must take place since a part of the internal energy distribution corresponds to the vibrational energy of protonated molecules in their ground state. The E/B linked scan in multi‐sector instruments for dissociations of relative high mass precursor ions (e.g., from 500 Da to one thousand or more). This is also illustrated by Cavelier's work (1999) studying transformation of cyclopeptide analog to CDP (Figure 10). Each of four sequences related to a particular amide linkage cleavage is detected (Cavelier et al., 1999).

Multi‐sector EBE, EBEB, and EBEBE or hybrid BEQ configurations were used to improve the resolution of the product ion spectra, to determine their elemental composition. High resolution can be used to select precursor ions and analyze the fragment ions produced in the collision cell of the fourth field‐free region (FFR4) of a five‐sector tandem instrument. In such an instrument, MS^2^ (in FFR4) and sequential MS^3^ experiments (i.e., the 1st generation of product ions occurs in FFR1 and the second in FFR3) can be performed. Note that from a selected precursor ion, it is possible to obtain the high energy collision spectrum of all the intermediate product ions, which dissociate in the FFR4 leading to the formation of a common fragment ion filtered by an adequate E/B linked scan (Lange et al., 1994). Such intermediate product ion spectra were generated to detect e.g., all the intermediate product ions from dissociations of protonated destruxin‐E (*m/z* 594, obtained by SIMS with 35 keV Cs^+^ beam on glycerol or nitro benzyl alcohol as matrix) which give rise to formation of the common *m/z* 194 product ion (i.e., the N‐acyl dipeptide acylium as described above, Loutelier et al., 1995). In addition, the protonated didemnin (with a peptidic side chain bonded to the amino group of threonine of a cycloheptadepsipeptide with an endocyclic ester linkage) desorbed in FAB, is characterized by a particular behavior towards high collision energy. This precursor ion dissociates to yield product ions following: (i) the loss of 1 or 2 residues (with low‐abundances) after ring‐opening and, above all, (ii) the loss of the neutral ring leading to abundant ions from the charge‐carrying peptidic side chain. Immonium species are also detected. This behavior differs from the dissociations observed at low energies, since ions with low *m/z* ratios due to the side chain are no longer observed and only ions with high *m/z* ratios due to the side chain are detected.

As such commercial tandem instruments have mostly “disappeared” from the laboratories, collisional processes under a high‐energy regime can be performed in instruments involving KE of several keV to several tens of keV for precursor ions colliding with helium as the target gas as it occurs with more recent double time‐of‐flight mass spectrometers (i.e., TOF/TOF tandem) smaller than the multi sector tandems. They quickly became predominant for high resolution structural analysis. However, no convincing example was found in the literature regarding their use for the detailed study of the structure of CDPs.

Finally, the important fact to remember is that these high energy collision spectra, although the collisional efficiency is low, show strong reproducibility (depending only on the studied precursor ion structures) compared to the low energy collision yielding product ion spectra (ion structure‐ and instrument‐dependent, *vide supra*). These high and low energy spectra are characterized by a wealth of complementary information (charge‐remote process *vs.* charge promoted dissociations).

###### Triple quadrupole, ion trap and hybrid HR devices and low energy collision regime

At the end of the 1970s, heavy multisector instrumentation was gradually abandoned in favor of benchtop of low resolution, such as low ion KE tandem instruments such as triple quadrupole introduced by Enke and Yost (1979). These latter allow low resolution MS/MS experiments under low energy collision conditions since only low KE ion beams can be analyzed using quadrupoles (as well as 3D and 2D ion trap). In this tandem, the first and the third quadrupoles are filter (ion mass analyzers) linked by scanning the direct current (DC) voltage and the radio‐frequency voltage amplitude (V_rf_). The second quadrupole is used as a collision cell which works in “RF only” mode meaning that the amplitude V_rf_ alone is scanned for total ion transmission without ion separation. For recording a product ion spectrum, the first quadrupole is fixed on the *m/z* of precursor ion for its selection and the third is scanned for transmission and detection of its product ions generated in the collision cell. These low energy tandem instruments are used for structural analysis, and particularly nowadays for quantification. This is due to (i) the shape of the ion signals owing to the stability of the device distributing either low DC voltage or high RF voltage, and (ii) the ability of each of these devices to go directly from a voltage to the other without scanning all the intermediate values.

Complementary to such ion beam instruments, sequential MS^n^ experiments based on ion storage in ion trap mass spectrometers (first with both *V*_rf_ and DC voltage linked scans and now, *V*_rf_ scan only) also allow low energy collision processes (*vide supra*) under low‐resolution conditions. Although only competitive dissociations mainly occur, consecutive ones can be achieved by performing sequential MS^*n*^ experiments. It can be used, as quadrupole, alone or combined with other analyzers (e.g. with quadrupole or TOF). In fact, the TOFMS‐based instruments are currently combined with quadrupole (Q) analyzer, for ion selection, followed by a quadrupole (q), in “RF only,” as collision cell. These tandems Qq/TOFs are called hybrid instruments since (i) a beam of low KE ions is selected by a Q quadrupole to be subjected to low energy collisions in the q‐cell, followed (ii) by the transmission of all the ions (survivor precursors and product ions resulting from activation) to the TOF analyzer where they are injected after high KE acceleration for their analysis in high resolution.

Other hybrid tandems with one or two multipoles (usually quadrupoles or ion traps) as above, allow reaching very high resolutions (100,000 to 1,000,000) where *m/z* ratio measurements are made by FT processing of the motion frequencies (transient signal) of all ions stored in a high vacuum. They can be recorded using either the ICR (Marshall et al., 1998) as a Penning cell for radial ion frequency measurements, or the orbitrap (Denisov et al., 2012; Makarov, 2000) derived from the Kingdon cell where axial ion frequencies are recorded. Fourier transform processing of the transient (evolution of frequency amplitude with storage time) produces all ion frequencies from which the masses of the ions are determined with very high accuracy, depending on the duration of the transient. Note that the orbitrap can be combined with quadrupole or ion trap analyzers, or both. Also, if the trap resonant excitation (radial excitation) is used to activate the ions, nonresonant excitation can take place in a trap where injection or ejection lens potentials are used (and not the radial excitation). This nonresonant excitation was called HCD by the instrumentation company (Olsen et al., 2007).

###### Fragmentations promoted by charge from positive molecular species under low energy collision conditions

**Figure 11:**
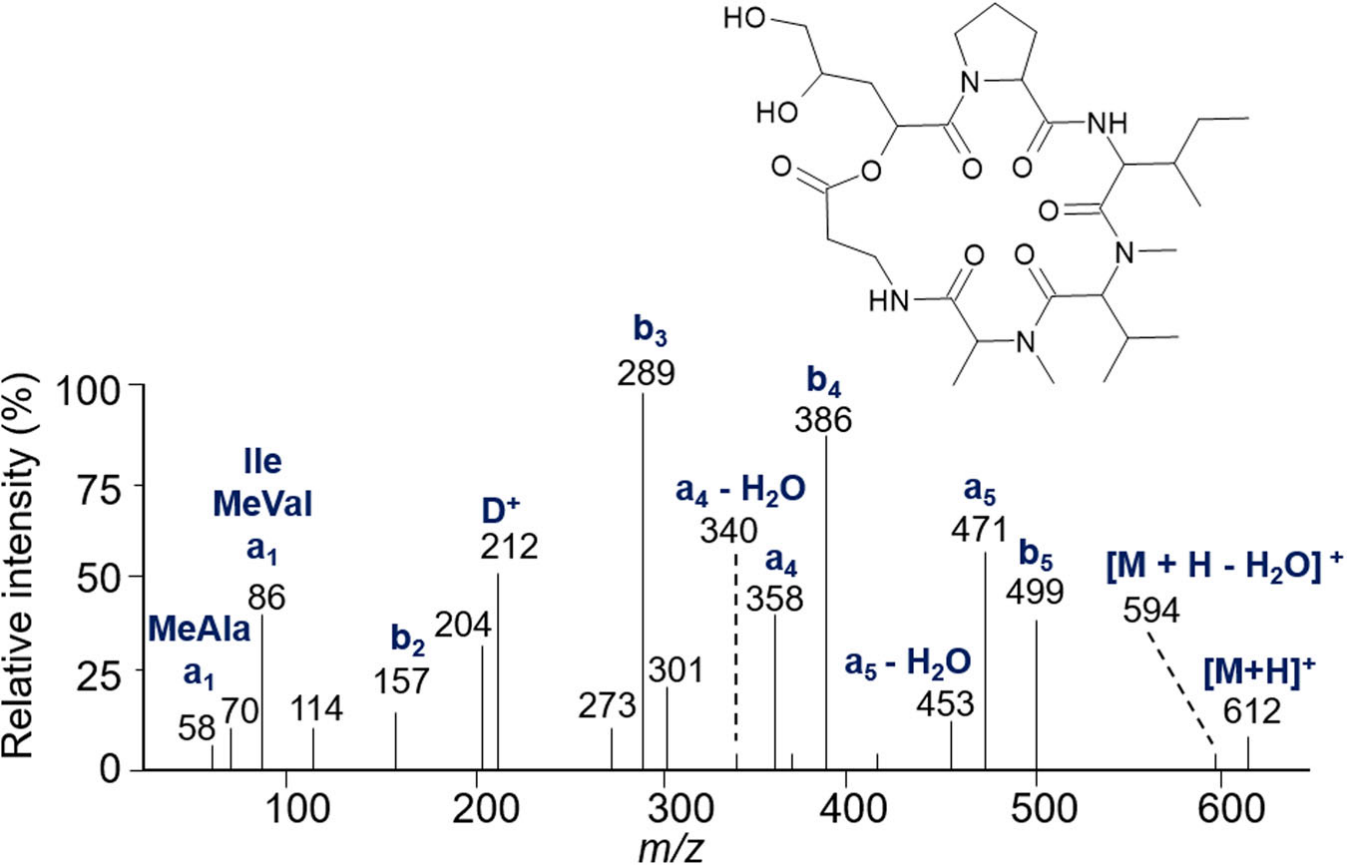
Prompt fragmentations by “in‐source CID” in mass spectra of destruxin Ed recorded under LC‐APCI conditions using a conventional desolvation voltage increased by 30 V using double sector instrument. Figure adapted from Jegorov et al. (1998).

The development of LC‐MS coupling involves ionization (and/or desorption) modes under AP conditions (i.e., APCI and ESI, as the main modes). This analytical approach is now of prime importance, mainly for structural confirmation or elucidation of natural extract from fungi, sponges, bacteria, marine cyanobacteria, or synthetic CDPs (Sivanathan & Scherkenbeck, 2014; Zeng et al., 2023). However, direct infusion into the API techniques has also been used in a few cases as well as in vacuum, using MALDI (Table 4). Under these API conditions, most of the molecular species produced are EE ions, mainly [M + H]^+^ (or/and [M + NH_4_]^+^) as common species generated in APCI and ESI. Their internal energy depends on the used desolvation step conditions. Consequently, their prompt fragmentations (known as “in‐source dissociation” or “in‐source CID”) are under our control through the desolvation conditions which is not the case with “in vacuum” ionization since, the internal energy depends on the ionization step. For instance, “in‐source dissociations” of protonated molecules generated from some 20 destruxins (produced by *Metarhizium anisopliae* and *Trichothecium roseum*) using LC‐APCI/MS, instantly generates their respective sequences (Jegorov et al., 1998). The APCI mass spectrum featured in Figure 11 shows the complete sequence of destruxin Ed, with the ring‐opening at the N‐methyl (NMe) amide linkage rather than at the ester bond often observed as the most reactive (*vide infra*). Thanks to this approach, the structures of destruxin Ed1 and roseotoxin A were obtained.

This review will focus on the fragmentation pathways of positive molecular species since negative ions have been poorly explored for CDPs, as mentioned earlier. Dissociative pathways for negative molecular species will therefore not be addressed, although their use could be important when acid residues (i.e., amino acid with gas phase acidic side chain or di‐hydroxylated fatty acids) are present in the sequence. Under API conditions, prompt dissociations being reduced (using soft desolvation conditions), the structural composition of molecular species and their adduct ions cannot be obtained from only the mass spectra. Nonetheless, under high resolution conditions, their elemental molecular composition can be obtained. The dissociations of ionized CDP mainly generated under API conditions, are formed after their selection, by gas phase activation in MS/MS experiments (Table 3). However, the orientation of fragmentations depends on the structural form of the studied CDP molecule. Therefore, it is relevant to figure out the possible form of the molecular structure in terms of tautomerism when the ionization/desorption takes place. Thus, in other word, in which form the ionized CDP should be considered during its gas phase activation.

####### Types of EE monocharged CDPs species considered for mechanistic interpretation of dissociation processes

The charged molecular species provided by ESI mode are characterized by a great diversity, independently of the polarity of the desorbed ions. Actually, (i) positive monomer or dimer ions such as protonated and alkali‐cationized or ammonium adduct species and (ii) negative ions as deprotonated or anion adduct species (RCO_2_^—^, inorganic anions) are desorbed according to the chosen experimental conditions. This wide variety of ions offers a lot of possibilities for MS/MS analysis. Some of these singly charged molecular species with an EE number generally in their first step lead to regeneration of either [M + H]^+^ or more rarely [M − H]^—^, respectively from ammonium or anion adduct species, whether they are monomers or dimers. The detection of such adduct or dimer ions shows a particular stability towards the desolvation steps, which is due to the hydrogen bond interaction between the molecule and the reactive ion partner. However, a strong increase of the desolvation voltage results in their dissociation into protonated (or deprotonated) CDPs. In alkali‐cationized molecules (i.e., with the most usual Na^+^ and/or K^+^), the cation is stabilized within molecule (in canonic form) through interactions with one (or more) free electron lone pair(s) of the heteroatoms of the molecule possibly yielding a *charge‐solvated* or *cation solvated* structure (CS). These CS forms are often used to describe alkali‐cationized CDPs, as some of them are considered as selective ionophores for a particular alkali metal ion in solution (Bauer et al., 2010; Clark et al., 2018; Rose & Jenkins, 2007). Especially, for certains CDPs a particular adduct ion is chosen for this property although the alkali‐cationized species can possibly be not the most abundant from the desolvation steps. Remember that this is typically the case of the potassiated cereulide considered as ionophore specific of the K^+^ cation as it occurs in solution (Ducrest et al., 2019; Marxen, Stark, Frenzel, et al., 2015).

However, to interpret the neutral losses consisting of one or more residues resulting of covalent bond cleavages (CBCs), we need to consider the opening of protonated or cationized CDPs (as discussed *vide infra).* It is as if a proton or an alkali metal cation was added to an open acyclic system with a positive charge at one end and a negative charge at the other (with balanced charges). This may mean that for alkali‐cationized CDPs, the molecular species formally are in zwitterion forms (may be non‐existing in solution) but that can desorb in positive mode as alkali‐cationized molecules (as well as protonated molecules). They are stabilized by a salt interaction between the zwitterion negative site and the alkali metal cation partner, resulting in the formation of a protonated salt structure (Colsch et al., 2017; 2019; Damont et al., 2019).

As CDPs are coming into their own as compounds with medicinal properties, one of the questions asked is what type of molecular interactions would allow them to penetrate and volt through cells membrane, land in the cytoplasm, and interact with their target? Looking at their structure shows that their molecular make up lends itself to noncovalent *interactions* such as salt bridges as they feature amino acids, as well as quaternary amine. When we hear the word “salt bridges” what immediately comes to mind are Na^+^ Cl^−^ or other metallic noncovalent complexes. However, biological ionic bonds mainly involves interactions between the side chains of charged amino acids: Arg, Lys (basic); Asp, Glu (acidic); Tyr, Trp, Phe (aromatic), and phosphorylated Ser, Thr, Tyr (Jackson et al., 2006; Woods, 2004), or lipids featuring quaternary amines (acetylcholines and sphingomyelins) or phosphate (phospholipids) (Barbacci et al., 2012). Such ion/ion interactions were shown to play crucial roles in GPCR receptors heteromerization (Ciruela et al., 2004) and their stability (Woods & Shelley, 2013), and receptor–drug interactions (Woods et al., 2003). In addition to being widespread, these Coulombic interactions are highly reproducible experimentally and amazingly stable (Woods & Ferré, 2005). Indeed, such complexes are very stable in the gas phase and under ion activation, they yield CBC rather than salt splitting (Darii et al., 2021; Woods & Ferré, 2005). The stronger the acidity of one partner and the basicity of the other, the stronger the interaction force is in the gas phase (Julian et al., 2002). This is a property required to generate zwitterion in desorbed aggregates.

###### Annotation used for the dissociation of the singly positively charged CDPs

Amino acid residues will be noted by the three‐letter code. Alpha‐hydroxy acid residues with a hydrocarbon chain identical to those of nonpolar‐chain amino acids (i.e., Gly, Ala, Val, and Leu/Ile) will be noted as their corresponding amino acid (i.e., ^O^Gly for 2‐hydroxyacetic acid, ^O^Ala for lactic acid, ^O^Val for 2‐hydroxyisovaleric acid, ^O^Leu for 2‐Hydroxyisocaproic acid, and Ile for 2‐hydroxy‐3methylpentanoic acid). For the others, initials of several letters will be used, but will be specified in the relevant text. The nomenclature used to annotate fragment ions from dissociations of the EE positive ions of CDPs (i.e., protonated, alkali‐cationized molecules, and ammonium adduct ions) is based on the Roepstorff‐Fohlmann‐Biemann nomenclature (Biemann, 1988; Roepstorff & Fohlman, 1984) as adapted for cyclopeptides by Ngoka and Gross (1999). It appears more suitable than other pertinent nomenclatures proposed for cyclopeptides (Eckart et al., 1985; Eckart, 1994). Other annotations were proposed for cationized cyclopeptides and CDPs (Eckart et al., 1985; Eckart, 1994; Williams & Brodbelt, 2004). Recently, Liuu et al. introduced a modified nomenclature suitable to annotate the low energy collisional spectra of EE molecular species of CDP such as alkali‐cationized cereulide (Liuu et al., 2024). This annotation combined with Biemann's and Ngoka's nomenclatures was extended to present one where the ring‐opening takes place through competitive cleavages of both the ester and amide linkages.

**Figure 12:**
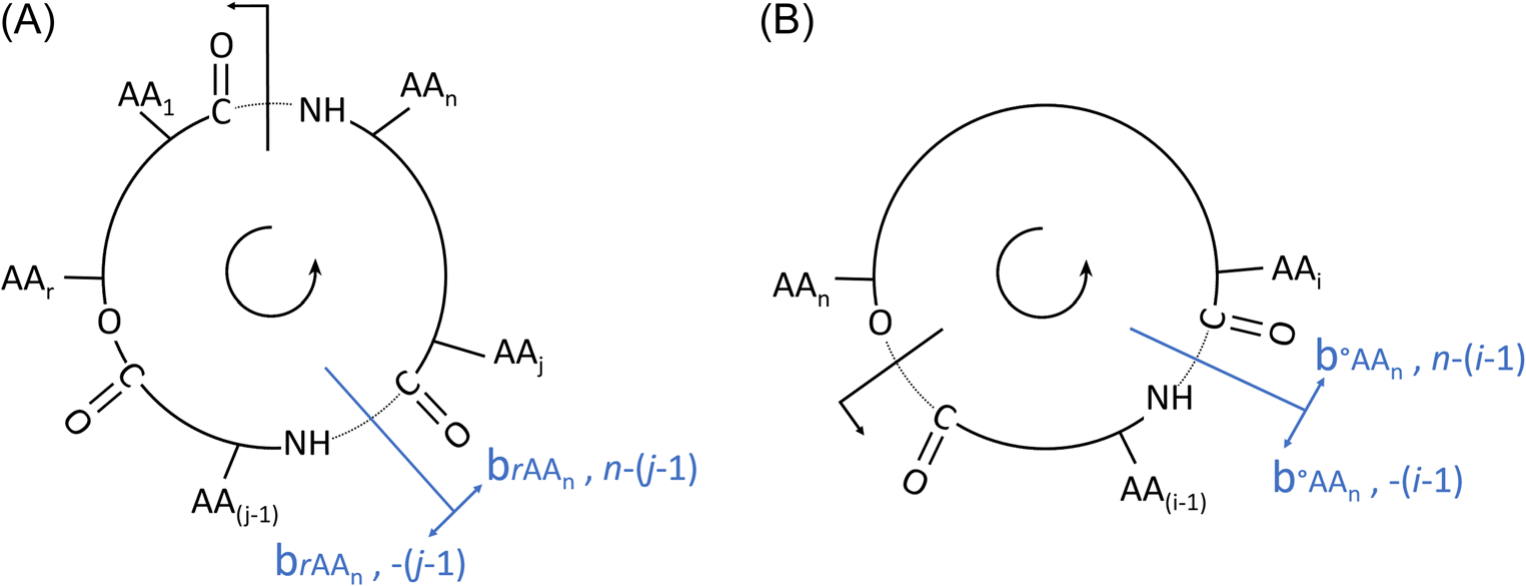
Illustration of the used descriptor for the **b** and **b**‐like series generated from dissociations based on a first cleavage (A) at an amide linkage and (B) at an ester bond from protonated cyclodepsipeptide.

In the largely applied Biemann's peptide cleavage nomenclature for protonated noncyclic peptides with an amino group and a carboxylic group at their ends, fragment ions related to the N‐terminus are noted as **a** and **b** ions, and fragment ions related to the C‐terminus as **y** ions. In the case of cyclic peptides, only the direct **a** and **b** fragment ions related to the N‐terminus sequence of linear peptide, are detected. The **y** series (C‐terminus sequence) is missed due to the absence of such terminus in a cyclic system (except from hydrolyzed CDPs resulting in linear peptide). Note that the (**b** + H_2_O) ions cannot be associated to the **y** ions although they have the same *m/z*. On the other hand, since only the N‐terminal sequence is to be considered and by convention this site is located on the left side of the open chain, the direction of the fragmentations will be in this direction, that is, counterclockwise. This orientation was considered long ago, in the case of EI of CDPs. Thus, the main fragmentations of the molecular ion of sporidesmolide III were described (Russell et al., 1964), where the prompt fragment ions of the mass spectrum are analogous to those of the **b** series (not yet defined as such). Consequently, this counterclockwise orientation will characterize fragmentations that will take place from protonated (or alkali‐cationized) CDPs made of n residues (nAA, one letter code was used to annotate the amino acid (AA) residue). The N‐(or O‐) terminus of **H**_**2**_ **N**‐CH(R_n_)‐CO‐ (or **HO**‐CH(R_n_)‐CO‐) arising from the opening of protonated CDP (same annotation for cationized substrate) should be first considered. This occurs between the two residues by the amide or ester linkage cleavage (shown as **‐\‐**) [HN‐(R_1_)CH‐**CO‐\‐NH**‐CH(R_n_)‐CO] and [HN‐(R_1_)CH‐**CO‐\‐O**‐CH(R_n_)‐CO‐] residues where for the amide bond cleavage, the chosen R_1_ side chain must be the smallest. From ring‐opening, the H_2_N group is present as a N‐terminus at the one end, and at the other end as an acylium site or more likely, a rearranged form into protonated oxazolone (Paizs & Suhai, 2005; Polce et al., 2000; Yalcin et al.,^1995^) appears which favors proton mobilization (acylium isomerization mechanism into protonated oxazolone described below and in §3.2.2.3.4.1.1, §3.2.2.3.4.1.2, and §3.2.2.3.4.2). This mobile proton promotes the release of the (HN‐(R_1_)CH‐**CO**, AA_1_) residue (i.e., the lightest) observed in the collisional spectrum of the EE molecular species. To locate this AA_1_ residue loss resulting from the second cleavage, the AA_1_ position, is annotated as the *r* position relative to the closer ester linkage in the counterclockwise orientation. The **b** product ion formed by the loss of the first amino acid AA_1_ residue located at the *r* position from the first ester linkage, is annotated in subscript as **b**_*r*AA_. For the AA_(*j*‐1)_—AA_J_ amide bond cleavage (with 1 < *j* < *n*) as the second cleavage resulting in the AA_1_—AA_(_ *_j_*_−1)_ neutral loss and the formation of a new acylium rearranged into protonated oxazolone at the C‐terminus then, the corresponding product **b** ion will be written as **b**_*r*AAn,(*n*‐(*j*−1))_ ion (Figure 12). In this case, the charge is retained in the counterclockwise fragment with a new acylium end or a new oxazolone form (the **b** series), and the subscript positive sign is attributed for (*n*‐(*j*−1)), that is, **b**_*r*AAn,(*n*‐(*j*‐1))_.

On the contrary, if the AA_*n*_—AA_*j*_ neutral is lost, then the subscript negative sign is introduced, that is, **b**_*r*AAn,‐(*j*‐1)_. If the initial rupture occurs at an ester linkage (‐HN‐CH(R_*n*_)‐**CO‐\‐O**‐CH(R_1_)‐CO‐) then, the opened CDP is written b_°__AAn_ without number before ^O^AAn. The product ion generated by a 2^nd^ cleavage between the AA_(*i*−1)_ and AA_*i*_ residues, will be noted as **b**_°__AAn,(*n*−(*i*−1))_.

###### Dissociations of EE CDP ions: Role of the charge and proton mobilization (prototropy)

Today, low energy collisions *(vide supra)* are the most popular mode used for activation of singly charged EE ions, although other activation modes exist and can be commercially fitted to the activation cell (e.g., IRMPD and EID) and were explored for CDP analysis. The following discussion will be limited to their ion activation properties and their application for CDP analysis as reported in Table 5. In general, these charged molecular species, vibrationally excited in ground, dissociate by heterolytic cleavages (through direct bond rupture, rearrangements, charge migration ….) essentially driven by the charge (Paizs & Suhai, 2005) according to the considered structure of the studied CDP. For positive molecular species, since proton (as well as after its mobility) promotes fragmentations, the coexistence of the protomer mixture (from desolvation steps or ion activation) must yield competitive cleavages from various possible proton locations. The fragmentation mechanisms must respect the rules of chemistry, especially those of the chemical reactivity as detailed in organic chemistry textbooks.

**Figure 13:**
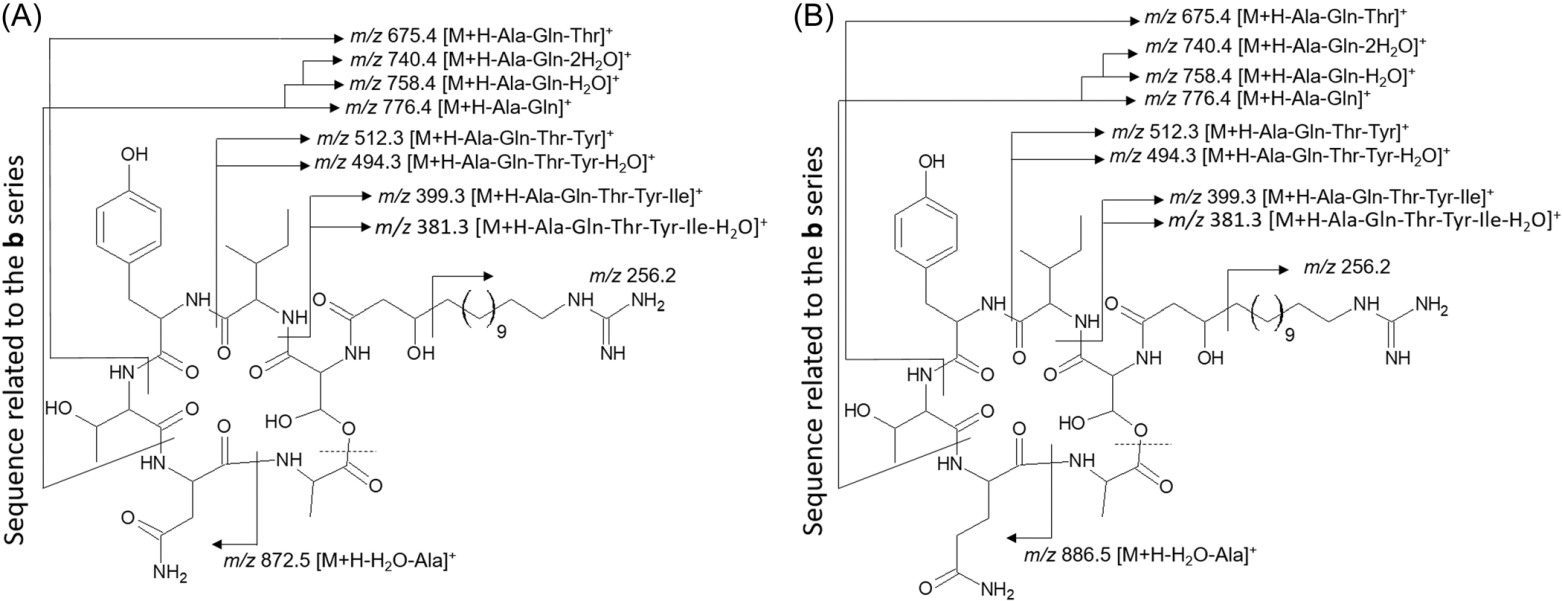
Symbolic fragmentations of the protonated molecules of the fusaricidin E family based on the **b** series processes with selective cleavage at the ester linkage containing (A) asparagine and (B) glutamine. Figure adapted from Reimann et al. (2017).

First of all, in the early 1960s, to rationalize the fragmentations characteristic of EI mass spectra of CDP, prompt fragmentations of molecular ion (with an odd number of electrons) were simply represented by a linear segment (oblique or orthogonal) on the bond to be broken, without explaining the cleavages by chemical mechanisms (Kiryushkin et al., 1967). Thus, neither the homolytic or heterolytic nature of these bond cleavages, nor whether they are simple ruptures or rearrangements, are specified although the first interpretation attempts were successful (Macdonald & Shannon, 1964). Unfortunately, the absence of fragmentation mechanism can be illustrated by the “symbolism” of molecular ion fragmentation interpretation of symmetrical and asymmetrical 12‐membered cyclotetradepsipeptides displayed in their EI mass spectra (Wulfson et al., 1964). Several successful formal interpretations were done for CDP from *Pithomyces chartarum* (Macdonald & Shannon, 1964). Again in 2017, the same approach was used for elucidation of fusaricidin E (Reimann et al., 2017) where compared to fusaricidin A (Kajimura & Kaneda, 1996), (i) a Val residue is exchanged by a Phe residue, (ii) the Asn residue for one homolog becomes a Gln (Figure 13), and (iii) the Ile is replaced by a Val. Fortunately, for several years now, mechanisms interpreting these fragmentations have been proposed and demonstrated for protonated linear and cyclic peptides (as EE species formed from an API source) from product ion spectra (Paizs & Suhai, 2005).

####### Dissociation of protonated CDPs (or ammonium/CDP adduct ions)

API methods can provide ammonium [M + NH_4_]^+^ adduct ions and/or protonated [M + H]^+^ molecules. For [M + NH_4_]^+^, ammonium and neutral partners interact together by hydrogen bonding which releases ammonia to give rise to formation of [M + H]^+^. The proton is covalently attached to lone‐electron pair of a heteroatom. It is mobilizable to migrate to lone‐electron pair of other conformationally neighbored site through hydrogen bonding intermediate. Such a proton transfer depends on the internal energy carried by the protonated molecule after desolvation steps. The respective [M + H]^+^ behavior towards low energy collision in the MS/MS experiments is independent of the API mode (i.e., APCI and ESI) which suggests that the distribution of the various [M + H]^+^ protomers is similar when they are formed through either gas phase ionization (i.e., APCI) or desorption (i.e., ESI). This can be explained by the mobile proton model (Paizs & Suhai, 2005) resulting in a similar protomer mixture before ion excitation.

The first question is the ring‐opening's orientation, which can take place from an amide bond or an ester bond for CDPs. In fact, the answer is not that simple. On the face of it, it could depend solely on the protonated site, but in fact it all depends on the collision energy and entropy effect (e.g., resulting in conformational hindrance). Indeed, the proton linked to the heteroatom constitutes a true catalyst (known in solution as “acid catalysis”) which after collisional activation, promotes the cleavage of a geminal bond to the carbon atom bound to the protonated heteroatom. Alternatively, this proton can competitively migrate to another “leaving group” and can induce its release by cleavage of its linkage to the carbon atom of the CDP skeleton. An old fragmentation rule introduced in positive CI can predict which bond is preferentially broken and which neutral is released. It is the one carrying the “leaving group” with the lowest proton affinity (Jardine & Fenselau, 1976). However, the experimental results are not so straightforward, since this depends on the internal energy of the system: at the lowest energies, rearrangements are favored, so the loss of the lowest basic group is observed, whereas at the highest energies, single ruptures are favored, so the loss of the most basic group can be favorably detected. This may explain the highly variable orientation of ring‐opening depending on whether the bond is an amide or an ester and may appear somewhat contradictory in the literature. In addition, this means that cleavage at the N‐methyl amide bond will be favored over that of the unmethylated amide bonds.

######## Dissociations of protonated CDPs with one or several NMe amide and ester linkages

**Figure 14:**
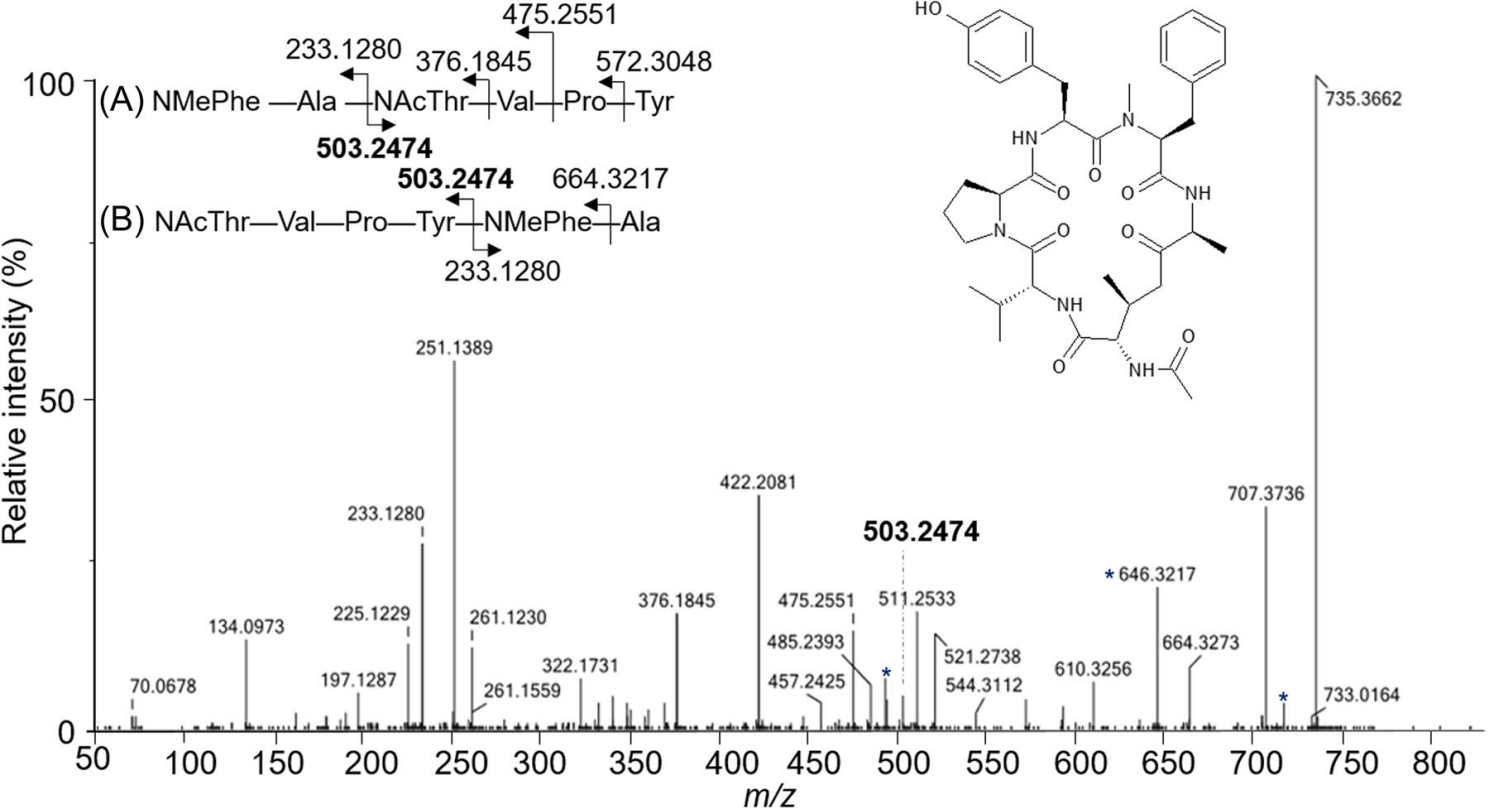
Product ion spectra of the protonated cyclohexadepsipeptide FJ120DPA (*m/z* 735) with the **b** sequence from (A) the N‐methyl amide linkage and (B) the ester linkage under high‐resolution conditions LC‐ESI‐MS/MS using Qq/TOF (the asterisk annotating certain signals is linked to the loss of water at *m/z* 735, *m/z* 664, and *m/z* 503, corresponding to the **b ion** series resulting from ring opening at the ester bond. Figure adapted from Hwang et al. (2019).

In all cases, the open form results in a secondary amine MeN(H)‐ (or a primary alcohol HOCH_2_‐ or secondary alcohol HOCHR‐ depending on the terminal residue) at the N‐terminus, while the C‐terminus is an acylium group (or a protonated oxazolone‐stabilized form) (Afonso et al., 2010; Paizs & Suhai, 2005; Polce et al., 2000; Yalcin & Harrison, 1996). In other words, the open structure is that of a **b** ion which dissociates yielding the product ions as the shorter **b** (and/or **a**) series but, also into other series such as (**b**‐H_2_O) when (i) the C‐terminus is hydroxylated or (ii) a serine or threonine residue is present in the sequence. The set of these product ions are also detected from CDPs without methylated amide linkage. In addition, the (**b** + H_2_O) series is also detected. This description of the product ion sequence is very useful to determine or confirm the CDP structure especially when a non‐amino acid (e.g., hydroxylated fatty acid) is partially included in the ring with the exocyclic aliphatic side chain yielding an ester linkage at one of its ends or a N‐acyl (NAc) amino acid such as Ser or Thr. This case is illustrated by the collisional spectrum of the protonated cyclohexadepsipeptide as FJ120DPA with the cyclo[NMePhe‐Ala**‐\‐**NAcThr‐Val‐Pro‐Tyr**‐\‐**] sequence (competitive ring‐openings are annotated by **‐\‐**) isolated from *Aspergillus ochraceopetaliformis* (Hwang et al., 2019). The presence of both the N‐methyl amide and ester linkages may differentially orient the competitive fragmentations of [M + H]^+^ (*m/z* 735) resulting in product ions of interest as indicated in the open‐ring structures (Figure 14). Although a wide fragmentation diversity characterizes its product ion spectrum, diagnostic ions are displayed but not the most abundant (Hwang et al., 2019). Two main **b** series, with each expected consecutive residue losses, appear to be initiated by the ring cleavage (i) at the NMe Phe/Tyr amide linkage (Figure 14A), and (ii) at the NAc Thr/Ala ester bond (Figure 14B) with a common product ion pair (*m/z* 503 and *m/z* 233). This pair of complementary ions suggests molecular isomerization into ion/dipole complex before dissociation from which interpartner proton transfer can occur (*vide infra)* (Afonso et al., 2010). Its formation is likely favored by the presence of secondary amino group (NMePhe) at one end of sequence, related to *m/z* 233, with a relatively higher proton affinity than that of the primary amino group at the end of sequence related to *m/z* 503 (explaining why the *m/z* 233 abundance is larger than the *m/z* 503 abundance). In addition, the loss of water from (i) the precursor ion, and (ii) the **b** series (through ring‐opening at the ester bond), together with the absence of the methylamine loss (through ring opening at the N‐methyl amide linkage) are consistent with the CI rule: the lower proton affinity neutral is preferentially released (Jardine & Fenselau, 1976) *(vide supra)*.

In another case, an effort to develop a systematic interpretation approach for the dissociation of the dudawalamide A extract from marine cyanobacteria *Lyngbya majuscule* (Liu et al., 2009) shows that cleavage orientation is favored at both NMe amide and proline linkages compared to the one at the ester bond which is hindered. The ring‐opening at the ester linkage compared to the NMe amide bond also occurred but is minor for cytotoxic veraguamides A and B (homologous to A by –14 *m/z*), alkynyl bromide‐containing CDPs from the marine cyanobacterium cf. *Oscillatoria margaritifera* (Mevers et al., 2011). The presence of the side chain influences the ring‐opening orientation. The A form ([M + H]^+^, *m/z* 766) is the most abundant. It carries the exocyclic alkynyl bromide side chain based on 8‐bromo‐3‐hydroxy‐2‐methyloct‐7‐ynoic acid with the polar β‐hydroxy acid head included in the ring, producing both ester and amide endocyclic linkages. In addition, the second aliphatic side chain with a 2‐hydroxy‐3‐methyl‐pentanoic acid (*Hmpa*) is similarly included in the ring (Mevers et al., 2011). The B form ([M + H]^+^, *m/z* 752) is characterized by the same peptide sequence where the *Hmpa* residue is modified into the 2‐hydroxy‐3‐methylbutyric acid (*Hmba*). The collisional spectrum of the veraguamide B mainly displays product ions with relative abundances similar to those of the A form, but their *m/z* ratios are shifted by –14 *m/z* (except one common *m/z* product ion generated from the loss of neutral containing the *Hmba* residue for B and the *Hmpa* unit for A). The stepwise formation of these product ions is initiated by competitive ring‐opening at the NMe amide and ester linkages followed also by these two favored cleavages. Surprisingly, the presence of bromide (or not) and/or unsaturation does not seem to affect the main double residue loss which involves: (i) the NMe amide ring‐opening, and (ii) cleavages at ester linkage between 8‐bromo‐3‐hydroxy‐2‐methyloct‐7‐ynoic acid and the second NMeVal residue (Mevers et al., 2011).

**Figure 15:**
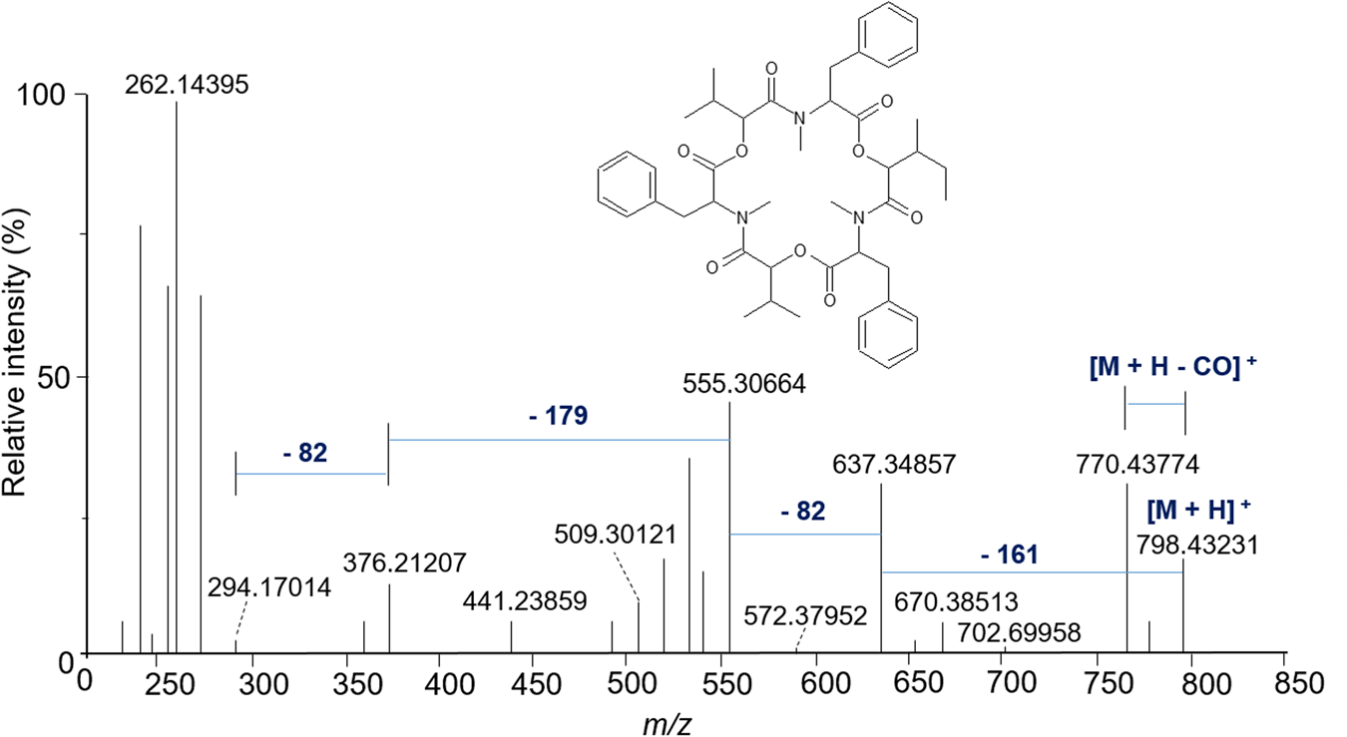
ESI product ion spectrum of the protonated beauvericin A, *m/z* 798 (as beauvericin structure where one Val residue is exchanged by one Ile/Leu) using LIT/Orbitrap instrument. Figure adapted from Daniel et al. (2017).

These competitive ring cleavages also characterize the dissociation of beauvericin A, a cyclohexadepsipeptide constituted by alternating methylated amide and ester linkages (Daniel et al., 2017). There are three complete and complementary sequences (except for each sequence, the last one or two terminal residue(s)) resulting from competitive ring‐opening cleavages of each ester bond (Figure 15). As for the three sequences associated with the cleavage of the methylated amide bond, their representative product ions are either common to those linked to the ester bond cleavage, or absent (one out of two).

However, the situation is not quite as simple, as the neighboring residues play an important role in orienting the ring‐opening in unexpected ways. This can be illustrated by the following example. The viequeamides A and B isolated from *Rivularia* sp. (with two endocyclic esters and a NMe amide) are major forms of the family of 2,2‐dimethyl‐3‐hydroxy‐7‐octynoic acid covalently bonded to CDPs (with five residues and one 2‐hydroxy‐3‐methyl‐pentanoic acid) (Boudreau et al., 2012). This occurs through an ester linkage (with the hydroxy group) and an amide bond (at the carboxylic site). In the extract, four other isoforms (C, D, E and F) were found and in particular, two isomers E and F (with one of two Val modified into either Leu/Ile, or NMeVal) were characterized by an unexpected behavior since their ring‐opening as initial cleavage seems to involve neither the NMe amide bond nor an ester bond but an NH amide bond.

####### Dissociations of CDPs with only NH amide and ester linkages

In the case of lipo‐CDPs, the side chain may play an important role in the fragmentation orientation. Only ester CBC of the fusaricidin E and homologue allow total sequencing with the Gln and Asn localization, both included between the Ala and Thr residues in the sequences, up to 15‐guanidino‐3‐hydroxy pentadecanoic acyl side chain by cleavage of the α‐β bond of the exocyclic amide linkage (Figure 13, Reimann et al., 2017). Although the dissociation mechanisms are not documented, the described fragmentation sequence involves the complete **b** series accompanied by the loss of one or two water molecule(s). They are related to the OH sequence end (related to the ester CBC) and the presence of hydroxylated amino acid herein, the threonine residue. The water is released from non‐amino acid hydroxylated side chain (e.g., hydroxylated fatty acids) for certain **b** product ions. Water elimination is expected since hydroxyl groups are characterized by lower proton affinity than that of amide or ester group. Interestingly, the hydroxy fatty acid‐like side chain is detected, very probably, due to the high gas phase basicity of the guanidino group.

Under ESI conditions, the iso‐isariin B and isariin E, extracted from the entomopathogenic fungus *Beauveria feline* (Langenfeld et al., 2011), yield the [M + H]^+^ (*m/z* 596 and *m/z* 666, respectively) and [M + Na]^+^ ions (*m/z* 618 and *m/z* 688, respectively). Both protonated molecules dissociate into the collision cell of Qq/TOF by a stepwise process. First, molecular species isomerization takes place by the exclusive ring‐opening at the ester linkage between the valine residue and the hydroxy group of the 3‐hydroxy‐4‐methyl octanoic acyl part (i.e., *Hma*, (C_4_H_9_)‐CH(CH_3_)‐CH(OH)‐CH_2_‐CO‐). After their ring‐opening, amide bond cleavage occurs (Bythell et al., 2009; Polce et al., 2000) giving rise to the formation of **b**, (**b**‐H_2_O), and (**b**‐CO) (or **a**) ions series (Langenfeld et al., 2011) which is consistent with previous studies of about a dozen of isariin homologs, whose ring is always opened at the ester bond (Ravindra et al., 2004; Sabareesh et al., 2007). The almost total sequence is obtained, with the last two residues, as it depends on the exocyclic chain(s). For instance, in the case of non‐ribosomal WS9326A produced by *Streptomyces asterosporus* strains (DSM 41452) (Zhang et al., 2018), the exocyclic chain being an aromatic system disubstituted with aliphatic chains is much less basic than the guanidino group (*vide supra*), and is not detected as a fragment ion. This explains why such a sequence is not fully described by the product ion spectrum of this protonated CDP.

**Figure 16:**
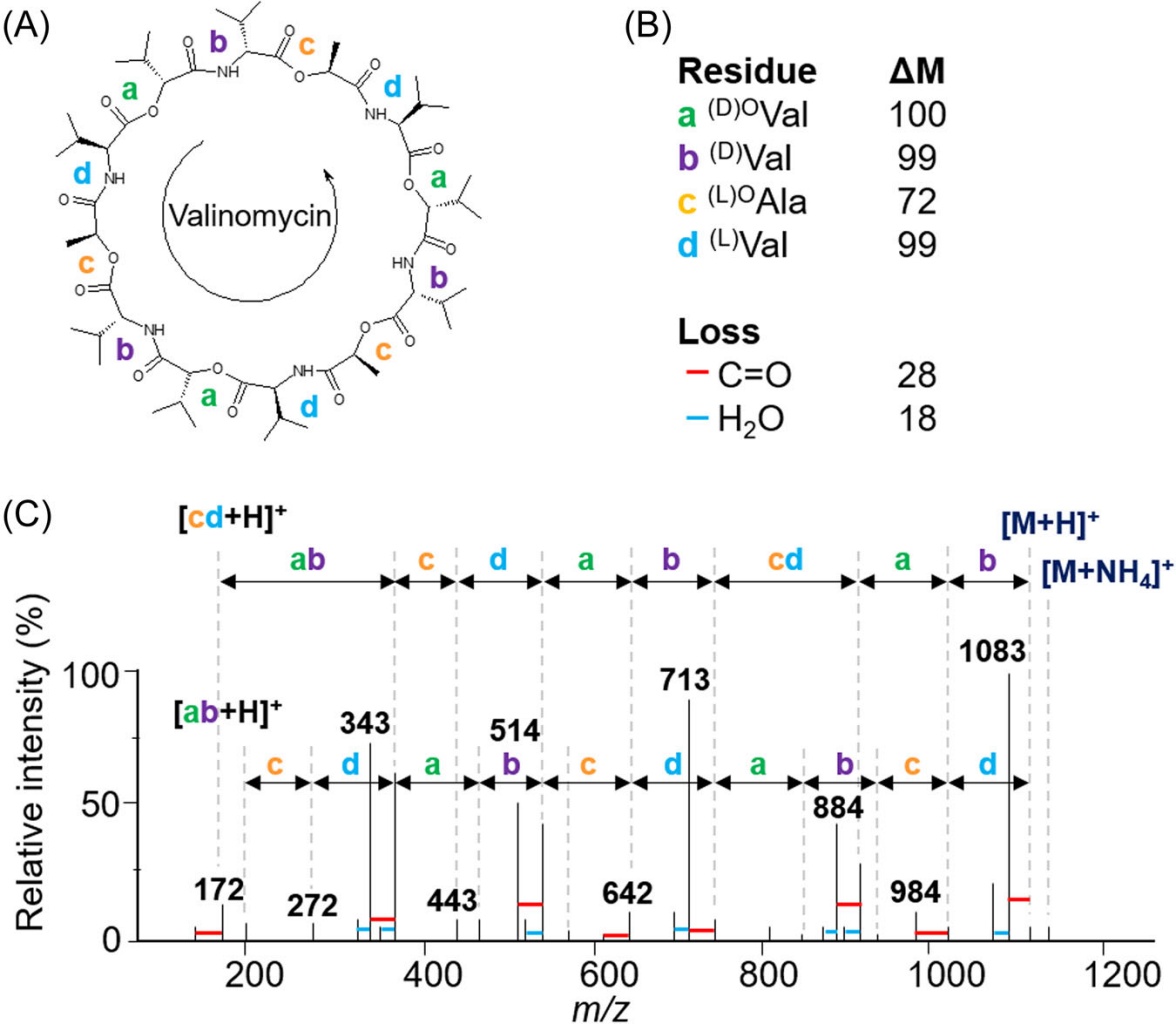
(A) Structure of valinomycin from *Escherichia coli* (the arrows on circle indicate the orientation of the bond cleavage for the **b** fragment ion series), (B) mass of each of the residues, and (C) ESI product ion spectrum of ammonium adduct species recorded under low energy collisions using triple quadrupole instrument (the H_2_O and CO losses are noted by a blue and red segment, respectively). Figure adapted from Jaitzig et al. (2014).

Without the presence of (i) an NMe amide and (ii) non‐amino acid side chain, protonated CDPs always involve the ester linkage to open the ring and produce the **b** ion series (accompanied by the other derivate series) with an NH_2_ (or NHMe) terminal or OH terminal. This is the case for beauverolide S, a nonribosomal CDP, specific markers of fungal infections (Jegorov et al., 2006). Amazingly, the same trend characterizes dissociations of the valinomycin from *Escherichia* coli (Jaitzig et al., 2014) as displayed in the product ion spectrum of the protonated species (Figure 16). The **a** series appears more abundant than the **b** series which differs from the previous examples. Two almost complete series (and derivate series) arise with different abundance according to the ester cleavage resulting in the ring‐opening: (i) the major one at the ^(D)^Val‐^(L)^°Ala linkage, and (ii) the minor one between the ^(L)^Val and ^(D)^°Val residues. This difference in cleavage efficiency is difficult to justify, even considering that compared to that occurring at the ^(D)^Val‐^(L)^°Ala linkage, the approach of the reagent to the ^(L)^Val‐^(D)^°Val ester bond may be made more difficult due to the steric hindrance created by the two isopropyl groups, depending on the conformation.

Recently, pagoamide A isolated from *Derbesi*a sp, a marine chlorophyte, consists of thiazolic side chain linked to the exocyclic chain of 11 amino acid residues. Its structure was obtained using molecular networks based on fragment ion spectra. Chirality of these residues was obtained using the Marfey's method (*vide supra* §3.1) and GC‐MS or LC‐MS (Li, Yu, et al., 2020). The dissociation, which begins with the opening of the ester, continues by eliminating residue by residue resulting in the **b** series up to the vicinal threonine at the side chain, although a lot of fragment ions remain uninterpreted. It's worth noting that when the polar head of the CDP's hydrocarbon side chain is a hydroxy acid and is included as amide and ester linkages in the CDP ring, the orientation of dissociations (ring‐opening and consecutive bond cleavage) depends on the proximity of these two linkages in pagoamide A. This behavior is also illustrated by the dissociations of the isariins I, II and III and the isaridins I, II and III as discussed later by comparison with cationized CDPs (Banerjee et al., 2011). Note that the presence of the exocyclic side chain might hinder the sequence determination as it occurs with the protonated plitidepsin (under Aplidin trade name) A and B (as well as their metabolites) which carry a peptidyl side chain. In fact, only the complementary ion pair due to the cleavage of the linkage between the side chain and the CDP moiety (with charge repartition in these two parts) is displayed in the product ion spectra recorded using triple quadrupole instrument under nonresonant ion activation conditions (*vide infra*, Brandon et al., 2005).

Finally, under low energy collision conditions, protonated CDPs dissociate mainly through stepwise processes into the **b** series and derivate series due to the CO or/and H_2_O loss(es), after the ring‐opening. This first step constitutes the essential step for the orientation of the residue losses which occur through consecutive or competitive pathways. The ring‐opening is dependent on the CDP sequence and is favored by the presence of the NMe amide due to its higher proton affinity compared to that of the NH amide. However, ring‐opening by cleavage of the NMe amide bond can occur in competition with that of the ester bond. This competition depends on the neighboring residues of the cleaved linkage in terms of steric hindrance and ring conformation, as well as the gas‐phase thermochemical properties (e.g., proton affinity) of any exocyclic side chain present. The role of the side chain is due to its direct involvement in the ester bond *via* a hydroxy group. When the polar head of the exocyclic side chain is a β‐hydroxy acid, the exclusive ring‐opening at ester linkages resulting in the **b** series takes place, while both **b** and **y** series are observed from the α‐hydroxy acid *(vide infra).* An important point that remains in the shadows concerns the effects of stereochemistry which, to our knowledge, have not yet been studied. This would make it possible to directly address the chirality of certain amino acids present in the sequence without having to go through chemical or enzymatic hydrolysis followed by GC/MS analysis for their determination. No systematic studies have been carried out using negative ions of intact CDP, such as [M−H]^−^ ions or adduct ions. The only route used involves ring‐opening hydrolysis with FDAA derivatization using Marfey's reagent (1‐fluoro‐2‐4‐dinitrophenyl‐5‐l‐alanine amide, *vide supra* §3.1) (Ishiyama et al., 2000). However, direct CDP analysis in MS needs attention, particularly the presence of highly gas phase acidic amino acid(s) (i.e., Asp and Glu, His or Arg in less extent) or highly basic (i.e., His, Arg, and Lys) in the sequence (not the case in the previous example). In these cases, it should be possible, under API conditions, to generate deprotonated CDPs under sufficient sensitivity to study their dissociations after collisional activation. They should strictly yield the ring‐opening at the ester linkage independently of the amide (methylated or not). Indeed, during the desolvation step based on the dissociation of anionic adducts, an alkoxide can be formed in the terminal position by opening the ester. This is due to the gas phase acidity of hydroxy end group being significantly higher than that of primary or secondary amino end group which cannot then be deprotonated.

####### Dissociation of alkali‐cationized CDPs, a neglected tool of the past yet useful for the future

As shown above for the dissociations of protonated CDPs, ring‐opening is often competitive on ester, N‐methylated, and N‐proline amide linkages, which sometimes confuses the product ion spectra and can therefore be difficult to interpret. Alkali‐cationized CDPs are often present in ESI mass spectra without the need to introduce an alkali salt, as mentioned above in the comments on ESI ionization/desorption. Among the various possible alkali‐cationized species, the sodiated CDPs are the most currently detected and are used, as well as the protonated CDPs, for molecular mass determination. Their abundance is generally sufficient for MS/MS experiments, even if the dissociation efficiency (i.e., collision energy) needs to be optimized. The potassiated CDPs are often detected in lower abondance than sodiated species. However, the potassiated species can be significantly enhanced compared to the sodiated CDPs when K^+^ is naturally introduced with certain CDPs when they are potassium‐specific ionophores in solution (e.g., cereulide; Ducrest et al., 2019; Marxen, Stark, Frenzel, et al., 2015; Walser et al., 2022). But this does not mean that such cationized molecules are more stable than the sodiated species in the gas phase. Concerning the lithiated CDPs, they are very rarely detected. This is also the case for larger alkali metals whose size is greater than that of potassium (i.e., Rb^+^ or Cs^+^).

Early on, a milestone study was carried out on the comparison of dissociations of sodiated and protonated CDPs (Ngoka et al., 1999). Collisions in the keV and tens of eV energy ranges were carried out, and those at low collision energies, although complementary, give rise to the most interesting dissociations. To reach larger sequence recovery from resonant excitation in ion trap instrument, sequential MS^n^ experiments were performed. However, the expected product ions consisting of one or two residues were absent due to chemical reasons (*vide infra*) or/and LMCO. It is required to perform sequential MS^n^ experiments (Ngoka & Gross, 2000) since consecutive dissociations are hindered (except for exothermic cleavages) (Tabet et al., 2021). The low energy collisional spectra show how the orientation of ring‐opening is specific at the ester linkage from sodiated species, whereas with protonated ones, ring‐opening is more random and occurs at both the amide and ester bonds. From the regioselective ring‐opening of the sodiated CDP at the ester bond, competitive cleavages of the ester and amide bonds take place, leading to the exclusive formation of cationized product ions covering almost the entire residue sequence up to the last two residues. The selected examples as beauvericin, didemnin B, and enniatin B1 illustrate this trend. Importantly, the sequence is completely described by the **b**, (**b**‐H_2_O), **a,** and (**a**‐H_2_O) ion series from sodiated CDP decompositions (Ngoka et al., 1999). This is not the case for the sequence obtained from protonated species fragmentation which missed several cleavages. The enniatins A1, B and B1 from *Fusarium* sp. are accompanied by two analogues whose sequences have been elucidated and are characterized by the sequences cyclo[^O^Val‐NMeVal‐^O^Val‐NMeVal‐^O^Val‐NMeThr] and cyclo[^O^Val‐NMeLeu‐^O^Val‐NMeVal‐^O^Val‐NMeThr] named enniatins P1 and P2, respectively (Uhlig et al., 2009). Their respective dissociations yield the **b** and (**b**‐H_2_O) series attributed to the ring‐opening step which occurs competitively at the three ester linkages. The origin of the water loss from the **b** fragment ions of sodiated enniatins P1 and P2 must differ from that described for the sodiated enniatin B1 (Uhlig et al., 2009). Sequence differences between 20 main congeners of beauverolides are obtained from dissociations of protonated and sodiated molecules under low energy collisions and again, they yield only the **b** and **a** series using ion storage experiments (in low resolution with ion trap and very high resolution with FTICR) (Jegorov et al., 2004). It should be noted that the retention of the alkali metal cation on all fragment ions is an important feature since it indicates that only the **b** series (and derived series) is produced with an acylium (and/or oxazolone or diketopiperazine) at one end and a sodium alkoxide salt at the other. This specificity also means that the Na^+^ cation does not migrate to another site (i.e., O^−^Na^+^ salt group is more stable than solvated cation site), unlike the mobile proton. The fragmentation mechanisms of ionized CDPs were also developed in detail later.

Alkali‐cationized CDPs from the family of *Bacillus cereus* emetic toxins have been extensively studied as they are powerful lethal toxins found in contaminated food (Dierick et al., 2005; Dirnhofer et al., 1977; Mahler Hellmut et al., 1997; Naranjo et al., 2011; Shiota et al., 2010; Takabe & Oya, 1976; Temper, 1963). Cereulide is described as an ionophore selective to K^+^ in solution, thus participating to mitochondrial swelling. This toxin provokes nausea and vomiting by acting on the vagus nerve (Glasset et al., 2016; Mikkola et al., 1999). Cereulide represents a wide chemodiversity as at least 18 cereulide variants (isocereulides) are obtained in the extract from *Bacillus cereus*. Among these ones, 14 variants (isocereulide A‐N) (Marxen, Stark, Frenzel, et al., 2015; Walser et al., 2022) were elucidated. Being considered as a K^+^ ionophore (Mikkola et al., 1999; Pitchayawasin et al., 2003), K^+^‐cationized species are chosen to identify cereulide and its congeners (Marxen, Stark, Frenzel, et al., 2015; Walser et al., 2022). For instance, two isomer pairs are identified such as: (i) cereulide as cyclo[^(D)^Ala‐^(L)^ ^O^Val‐^(L)^Val‐^(D)^ ^O^Leu]_3_ and isocereulide G, and (ii) isocereulides A and F, characterized by their potassiated forms at *m/z* 1191 and *m/z 1205,* respectively.

Their respective sequences were elucidated in HRMS with Qq/TOF and ion trap using sequential MS^n^ experiments (with *n* = 5 or 6 steps) and only one tetrapeptide unit of cereulide (^(D)^ ^O^Leu‐^(D)^Ala‐^(L)^ ^O^Val‐^(L)^Val) is modified for the A, F and G variants. First, a pair of residues is inversed in the sequence of the variant G isomer to the cereulide which is modified in cyclo[(^(D)^ ^O^Leu‐^(D)^Ala‐^(L)^ ^O^Val‐^(L)^Val)_2_‐(^(L)^ ^O^Val‐^(L)^Val‐^(D)^ ^O^Leu‐^(D)^Ala)]. Relative to one tetrapeptide unit of the cereulide, ^(L)^ ^O^Val is replaced by ^(L)^ ^O^Leu for variant A in contrast to its isomer, variant F, the sequence becomes (^(L)^ ^O^Val‐^(L)^Val‐^(L)^ ^O^Val‐^(L)^Val) (Marxen, Stark, Frenzel, et al., 2015). More recently, the proposed ^(L)^ ^O^Leu residue of the modified tetrapeptide unit in variant A has been re‐examined and has been replaced by ^(L)O^Ile (Walser et al., 2021). Thus, the corrected variant A sequence was changed to cyclo[(^(D)O^Leu‐^(D)^Ala‐^(L)O^Val‐^(L)^Val)_2_‐^(L)O^Val‐^(L)^Val‐^(L)O^ **Ile**‐^(D)^Ala].

**Figure 17:**
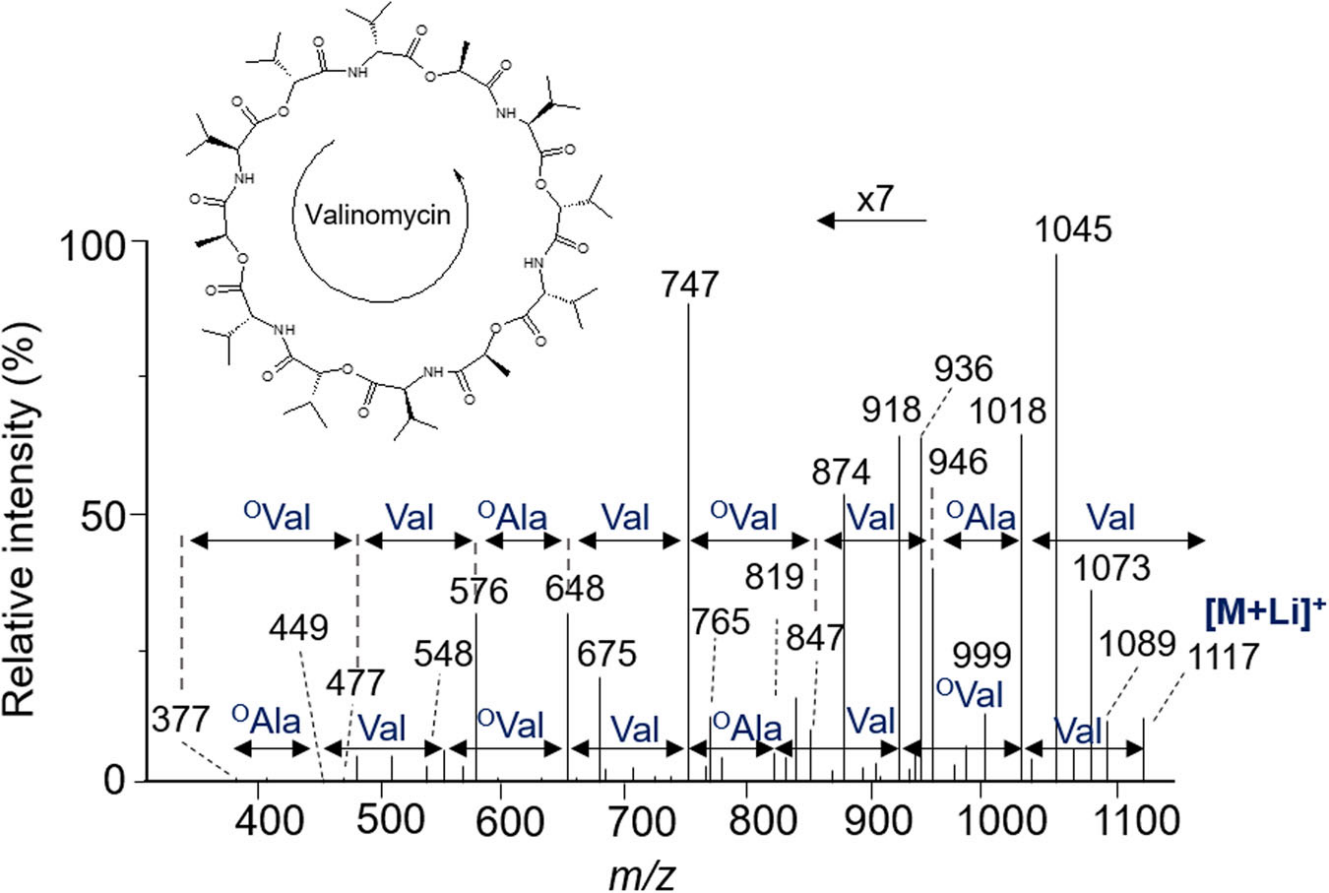
ESI product ion spectrum of the lithiated valinomycin using ion trap instrument. Figure adapted from Ngoka and Gross (2000).

In these examples, the fact that (i) only ester bonds are involved in ring‐opening, and (ii) the potassium cation is retained in the fragment ions, means that this cation could be used as a marker of the sequence mostly characterized by the **b** product ion series (and derivatives). Other alkali metal ions were employed such as the Li^+^ and Na^+^ cations for the cereulide structural investigation using Qq/TOF and Orbitrap‐based tandem (Liuu et al., 2024). Historically, the lithiated CDPs such as valinomycin was used to resolve its sequence (Ngoka & Gross, 2000) using the same ion storage instrumentation with the same limitation in LMCO and low resolution. The residue sequence of the lithiated valinomycin was determined and is characterized by the regioselective ring‐opening at the two ester linkages which results in two **b** product ion series (Figure 17).

In the case of this CDP, in addition to alkali metal ions (Li^+^, Na^+^, K^+^, Rb^+^, Cs^+^), various cationized forms with different classes of metals have been investigated (Williams & Brodbelt, 2004). These include simply and doubly charged metals such as alkaline earth metal ions (Ca^2+^ and Sr^2+^) or transition metals (Fe^2+^, Co^2+^, Ni^2+^, Co^2+^, Cu^2+^, Zn^2+^, and Ag^+^) and another as Pb^2+^. Interestingly, the broadest sequence coverage involves Li^+^, Na^+^ and Pb^2+^. It's not just the properties of alkalis that favor access to the CDP sequence, but also their size. Indeed, adduct ions with Rb^+^ and Cs^+^ lead to a very low fragmentation yield which results in a strong sensitivity degradation. This will be explained later. In the case of doubly charged cations, the stronger interactions between the metal cation and free heteroatom lone‐pair electrons mean that fragmentation will be oriented toward favored processes which prevent complete sequence recovery.

Other classes of CDPs are known to be substituted by an exocyclic chain, either peptidic or aliphatic (without or with one or more functions and/or ramification(s)). In one way, this chain forms either an ester or amide bond with the polar side chain of a residue (e.g., Thr or Ser, Asn or Gln). Alternatively, it can be partially included by two of these functions (e.g., ‐OH or/and ‐COOH), forming ester and amide linkages with the peptide N‐ and C‐terminus resulting into the backbone of a CDP (*vide supra*). Their structures are often investigated by using sequential MS^n^ experiments under resonant excitation conditions in an ion trap for the stepwise sequencing of the cationized CDPs after its ring‐opening which is exclusively oriented on the ester bond cleavage. This can be illustrated by the dissociations of the sodiated tiahuramides A, B, and C. (Levert et al., 2018). These CDPs are constituted by two ester linkages involving two α‐hydroxy acids (i.e., (3 S)‐phenyllactic acid (*Pla*) and (3 S,2 R)−3‐hydroxy‐2‐methyloct‐7‐ynoic acid (*Hmoya*) for tiahuramide A or −7‐enoic acid (*Hmoea*) for the B isoform or the saturated (3 S,2 R)−3‐hydroxy‐2‐methyloct‐7‐anoic acid (*Hmoaa*) for the tiahuramide C). So, their generic structure is cyclo[^(L)^Val‐NMe^(L)^Val‐*Pla‐* ^*(L)*^Pro‐NMe^(L)^Leu**‐\‐** *Hmoxz*] (with *Hmoxz* as *Hmoya, Hmoea* and *Hmoa*a). Regardless of the *Hmoxz* structure, the sodiated product ion of each consecutive fragmentation characterizing the sequence remains common with a variation of +2 *m/z* (for *Hmoea* in tiahuramide B) and +4 *m/z* (for *Hmoaa* in tiahuramide C) compared to that of *Hmoya* in tiahuramide A (Levert et al., 2018). In the case of plitidepsins A/B (aplidins A/B) and their metabolites, after ring‐opening at the ester linkage, several product ions of the CDP allow the determination of major part of the peptide sequence unlike the protonated species which yield ion/dipole leading mainly to two copmplementary product ions (Brandon et al., 2005).

**Figure 18:**
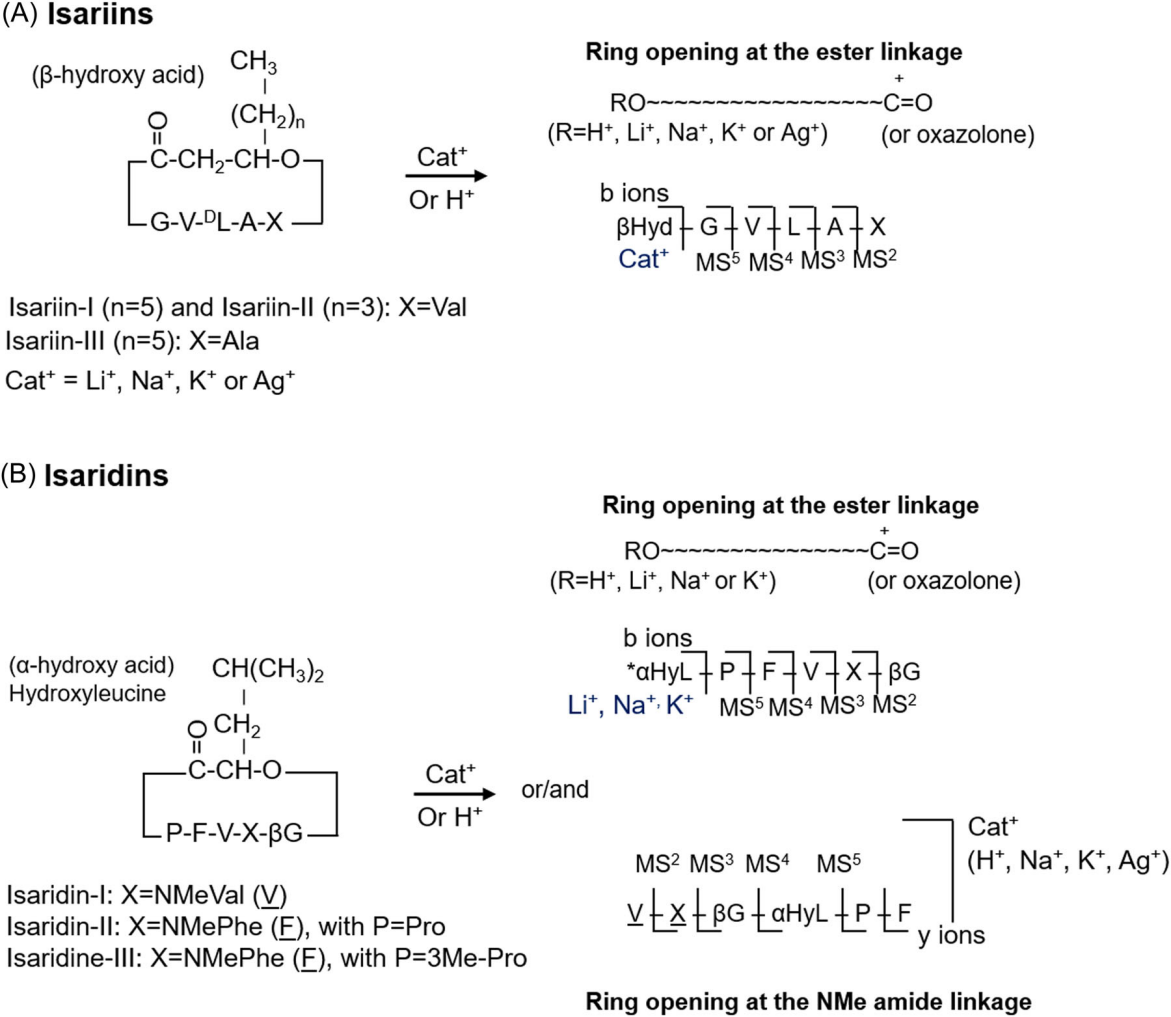
Summary of ring opening and fragmentation sequences for protonated and cationized CDPs (with Cat^+ ^= Li^+^, Na^+^, K^+^, Ag^+^) for (A) isariins I, II and III (different side chain length of ß hydroxy acid; with X being Val or Ala), and (B) isaridins I, II, and III with: (i) the common ‐CH_2_CH(CH_3_)_2_ side chain of α hydroxy acid, (ii) NMe amide linkages for Val (V), and Phe (F), (iii) P being proline or 3Me‐proline and (iv) ßG as: ‐NH(CH_2_)_2_CO‐. In all cases, one and two residues are missed from potassiated species. Figure adapted from Banerjee et al. (2011).

CDPs involving aliphatic β‐hydroxy acids are endocyclically linked by an ester bond and an amide bond with the peptide end groups providing the cyclic system. Such a structure is illustrated by that of the isariin variants I, II, and III (Banerjee et al., 2011). Protonated and Li^+^/Na^+^/K^+^/Ag^+^‐cationized CDPs were characterized in resonant mode using sequential MS^n^ experiments. The spectra of the cationized isariin variants display cationized fragment ions of the **b** series from the ring‐opening at the ester bond, giving the almost complete sequence, except for K^+^ where the series is not complete. In the case of α‐hydroxy acids with NMe residues, such as isaridin variants I, II and III present a different behavior. Cationized with the same cations, the **b** and **y** product ions series are formed in competition depending on the cation considered: (i) with Li^+^, ring‐opening takes place strictly at the ester bond and only the **b** series is produced, (ii) on the contrary with Ag^+^, opening takes place exclusively at an NMe amide bond and only the **y** series is obtained, while (iii) with Na^+^ and K^+^ cations, as well as the proton, both the ester and NMe amide bonds are competitively cleaved and the **b** and **y** series are produced. All these pathways determined by MS^*n*^ are summarized in Figure 18. Moreover, there is always cation retention on the ions produced in the **b** and **y** series (Banerjee et al., 2011).

###### Mechanisms of EE CDP ion dissociation

####### Proposed mechanisms of the protonated CDP dissociations

######## Current fragmentation mechanisms

The main “rule” to remember concerns the role of charge in the heterolytic mechanisms proposed for interpretation of the gas phase dissociation of simply charged ions with an even number of electrons. Indeed, charge (e.g., the proton for positive ions) leads to covalent bond cleavage either directly or post proton migration. This process is an alternative of charge‐remote like processes where the charge is a spectator to the cleavage, as considered for the interpretation of the fragmentation of various beauvericins (consisting of ester, NH amide, NMe amide and N‐proline linkages). The ring‐opening essentially occurs at the NH amide linkage from protonated CDPs (Daniel et al., 2017). The **y**‐like series is described. A similar behavior is seen from protonated viequeamides (Boudreau et al., 2012). For the 22 destruxin congeners consisting of one or two NMe amide bonds and an ester bond, different ring‐opening orientations take place. Depending on the structure, the cleavage of one of the NMe amide bonds (or at N‐amide of proline) induces ring‐opening. The role of ester seems to be limited under these resonant excitation conditions using ion trap instruments (Seger et al., 2004). Alternatively, in protonated enniatins and bassianolides, the ring‐opening takes place at the NMe amide linkage. In the case of the veraguamide familly (Mevers et al., 2011), the role of proton is not described. However, the ring‐opening takes place competitively at both the ester, and NMe amide linkages (without involving the N‐proline amide). In this case, the **b** series is observed instead of the **y**‐like series. Other examples show competitive NMe amide and ester bond ring‐opening, such as the protonated cyclohexadepsipeptide FJ120DPA (*vide supra,* Figure 14) characterized by the **b** series (Hwang et al., 2019).

To date, the interpretation of the orientation of the ring‐opening mainly depends on the structure of the CDPs, although the mechanism of fragmentations yielding the sequence attribution remains unclear. This competitive ring‐opening contrasts with the ester bond cleavage promoted by the mobilizable proton resulting in the ring‐opening of the protonated beauverolide S (Jegorov et al., 2006). For example, protonation at either NMe amide or N‐amide of proline bonds should result *a priori* in more stable species than of the protonated NH amide and the ester linkages due to their lower proton affinities. On the other hand, this means that ring cleavage rate constant of the latter should be larger than that of both the NMe amide and the N‐Proline protonated species. Nevertheless, a reversed situation is observed (*vide supra*). An explanation is proposed by taking into consideration that the endothermic proton transfer from NMe amide to the NH amide and ester linkages requires too much internal energy. However, the collision energy increase may favor the direct NMe amide bond cleavage rather than the expected proton transfer to the NH amide or ester linkage. Indeed, due to its relatively low frequency factor, this proton transfer may be disfavored relatively to the direct cleavage, by the increasing collision energy.

Even if generally, the **b** series is the preferred dissociation pathway, examples from literature do not follow such a trend. This is the case for the kulolide superfamily CDPs such as the viequeamides (Boudreau et al., 2012). For instance, the viequeamide E made of two endocyclic ester and NMe amide linkages (i.e., cyclo[*Dhoya*‐Ile‐NMeVal‐*Pla*‐Pro‐NMeAla**‐\‐**Val]) (with *Dhoya*: 2,2‐ dimethyl‐3‐hydroxy‐7‐octynoic acid). Its collision spectrum displays the **y** product ion series which is surprisingly initiated by the NH amide linkage ring‐opening (annotated as: **‐\‐**) followed by the valine residue neutral loss from ester bond cleavage at the *Dhoya* residue. The same ring‐opening characterizes all six viequeamides studied, and the abundances of their product ions are comparable despite their modifications. Note that this ring‐opening contrasts with the NMe amide or ester linkage cleavages which currently takes place from dissociation of protonated CDPs.

**Figure 19:**
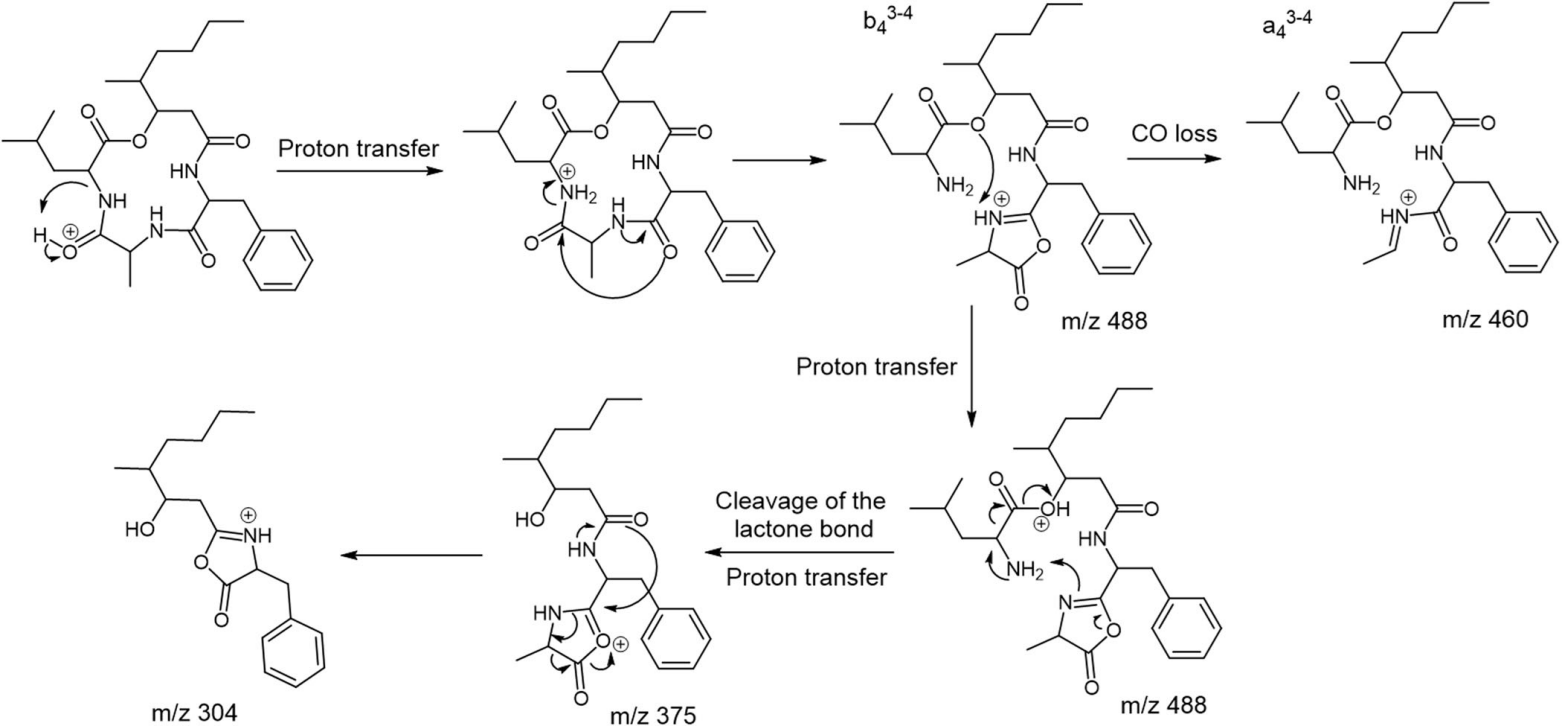
Proposed mechanisms of dissociation of protonated beauverolide I (*m/z* 488). Figure adapted from Jegorov et al. (2004).

Finally, all studies show that it is difficult to predict *a priori* whether the ring‐opening is competitively initiated by the endothermic proton migration or by direct cleavage competition since all possibilities are feasible. A comprehensive review of peptide dissociation by Paizs and Suhai (2005) based on proton mobilization confirmed by reaction calculation and modeling presents different charge‐induced cleavage pathways as applied to dissociation of the protonated extraribosomal cyclotetradepsipeptides such as the protonated beauverolide I (*m/z* 488) (Jegorov et al., 2004). This is a stepwise process which is initiated by inducing the ring‐opening at the ester linkage rather than at the amide bond (Jegorov et al., 2004; Paulo et al., 2019). This step can directly yield hydroxy group at one end of opened ring and acylium (Ngoka & Gross, 2000; Ngoka et al., 1999) at the other end, although the proton mobilization involving the chiral CH site changes to acylium (Daniel et al., 2017; Sabareesh et al., 2007). Alternatively, the formation of oxazolone (or diketopiperazine eventually) end group instead of acylium takes place preferentially either after the ring‐opening step or quasi‐concomitantly with the ring‐opening (Figure 19) (Jegorov et al., 2004).

For each of these ends, the H_2_O and CO losses occur competitively and yield *m/z* 470 and *m/z* 460, respectively. From the opened form of [M + H]^+^ isomerized into protonated oxazolone, after the release of a α‐lactam neutral (Jegorov et al., 2004), rearrangement of the resulting product ion end with the neighboring residue lead to the regeneration of another protonated oxazolone (Figure 19), and so on. While in the direct pathway, the **b** series as product ions (i.e., *m/z* 375 and *m/z* 304 related to the loss of the Leu and Leu‐Ala residues) are provided as well as the consecutive (**b**‐H_2_O) and (**b**‐CO) product ions. In addition, depending on the residue sequence make up, through internal proton transfer into the ion/dipole complex (i.e., based on proton‐bound dimer structure) formation, the **y** series can be provided depending on the collision energy conditions (Paizs & Suhai, 2002, 2005).

######## Stepwise processes through ion‐dipole of protonated CDP before formation of product ions

*S*uch a situation may be encountered from positive ions of low internal energy resulting in long‐life species favorable to molecular isomerization. This involves the bond cleavage(s) yielding fragment ions solvated by their associated neutrals through hydrogen bonding interaction. The closer the proton affinities of the two partners, the longer the lifetime of the ion/neutral complex is (Afonso et al., 2010). As a result, rearrangements such as competitive internal proton transfers are possible before separation of the ion/neutral complex. Following these competitive proton transfer, complementary product ions are released by the splitting of each protomer complex.

**Figure 20:**
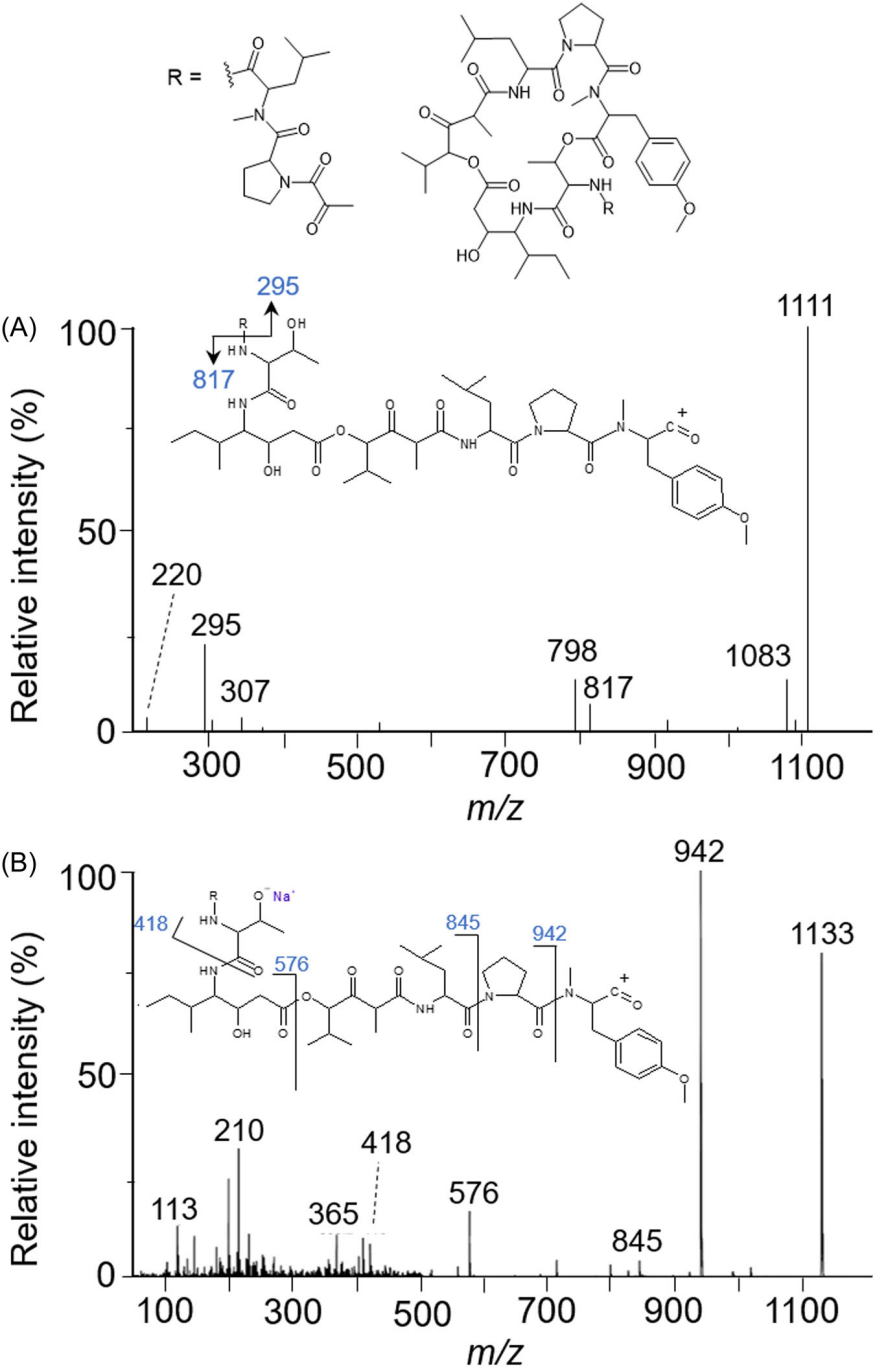
ESI product ion spectra of (A) the protonated and (B) the sodiated plitidepsin A and B mixture from a triple quadrupole instrument. In (A) the cleavage yielding ion/dipole complex is illustrated and (B) the **b** sequence ion is annotated. Figure adapted from Brandon et al. (2005).

Interestingly, in addition to the **b** series observed from dissociations of the protonated cyclohexadepsipeptide from fungi *Aspergillus ochraceopetaliformis (vide supra,* Figure 14, Hwang et al., 2019), product ions are detected resulting from stepwise processes through internal proton transfer before dissociation. The collision spectrum of the protonated FJ120DPA displays two product ions at *m/z* 503 and *m/z* 233 which are complementary in elemental composition (with one more proton, compared to the protonated precursor, within 2.2 ppm of mass error, Figure 14). Their formation is initiated by the protonated CDP opening either at the NMe amide bond or at the ester bond. Consecutive bond cleavages at either ester or NMe amide bond from both these two isomeric open forms yield before dissociation the intermediate ions that are stabilized by hydrogen bonding between the terminus N‐methyl amine of one partner and the terminus oxazolone of the other. They are ion/dipole complexes which dissociate by competitive splitting, into the complementary product *m/z* 503 and *m/z* 233 ions. Such a proton exchange is in favor of the terminus HNMe‐ group to generate *m/z* 233, while the oxazolone forms the complementary *m/z* 503 ion. Indeed, the proton affinity of the secondary amine is higher than that of oxazolone (Nold et al., 1999). Herein, such a stepwise isomerization into ion/dipole before dissociation of the protonated FJ120DPA does not affect the other dissociation pathways yielding the **b** series through classical processes. However, it is not always the case since the formation of ion/neutral complexes can hinder all other product ion series, as illustrated by the comparison of the collisional spectra of plitidepsins (*vide supra*), and their metabolites (Brandon et al., 2005). Especially, the plitidepsin (as a conformer A and B mixture, *m/z* 1110) consists of an exocyclic peptidyl side chain linked to the amino group of the threonine residue included in the ring by its secondary hydroxy group (endocyclic ester linkage) and its carboxylic group giving endocyclic ester and amide linkages, respectively. A second endocyclic ester linkage is introduced with the α‐hydroxy‐methyl‐3‐butyric acid (Figure 20A). Under nonresonant excitation conditions, the protonated plitidepsin dissociated mainly through a major pathway, involving cleavage of the exocyclic amide linkage. The peptidyl side chain is then released with a charge distribution at the complementary product ions: (i) the exocyclic peptidyl side chain acylium at *m/z* 295 and (ii) the CDP moiety (*m/z* 817) (Figure 20A). These complementary ions are generated very likely from ion/dipole complex intermediate, although the mobilizable proton transferred to a partner is not generated from the oxazolone or the diketopiperazine nitrogen atoms which do not carry proton. This means that only the proton at the chiral site (the α position of acylium) is mobilizable resulting in a ketene terminal group after its transfer. On the other hand, product ions from the **b** series are not observed as expected. Indeed, NH site, adjacent to the ring‐opening position, is absent, so the proton induced dissociation is not possible. From the metabolites of plitidepsin such as the apli‐h 1 and 2 congeners (*m/z* 1126), their protonated forms are characterized by product ion spectra with similar trends, since only the complementary *m/z* 295 and *m/z* 832 ion pair is detected (Brandon et al., 2005).

Finally, ring‐opening and the orientation of subsequent fragmentations are highly dependent on the structure of the CDP, that is, the presence of ester and N‐methyl amide bonds, including side chains (considering neighboring groups to endo/exocyclic linkages). As a result, sequences are sometimes difficult to determine, as often certain fragment ion of the **b** series are missed without obvious reason. In addition, there is an overlap of sequences with different endings defined by competitive cyclic openings, making it difficult to determine the CDP sequence. Comparison of fragmentations with CDPs from the same family is an alternative, although only applicable in special cases. In other words, many structural studies of CDPs based on the dissociation of protonated molecular species are not sufficient. MS/MS of protonated molecules is often combined with NMR analysis to confirm proposed structures. However, for complex mixtures where LC is required, high‐resolution MS/MS is essential. It is therefore desirable to find molecular species whose dissociations limit ambiguities by reducing the number of sequences that could overlap. For this, the study of fragmentation of cationized CDPs may be a better alternative.

####### Proposed mechanisms for the dissociation of cationized CDPs provided from a particular specific “in situ derivation”

**Figure 21:**
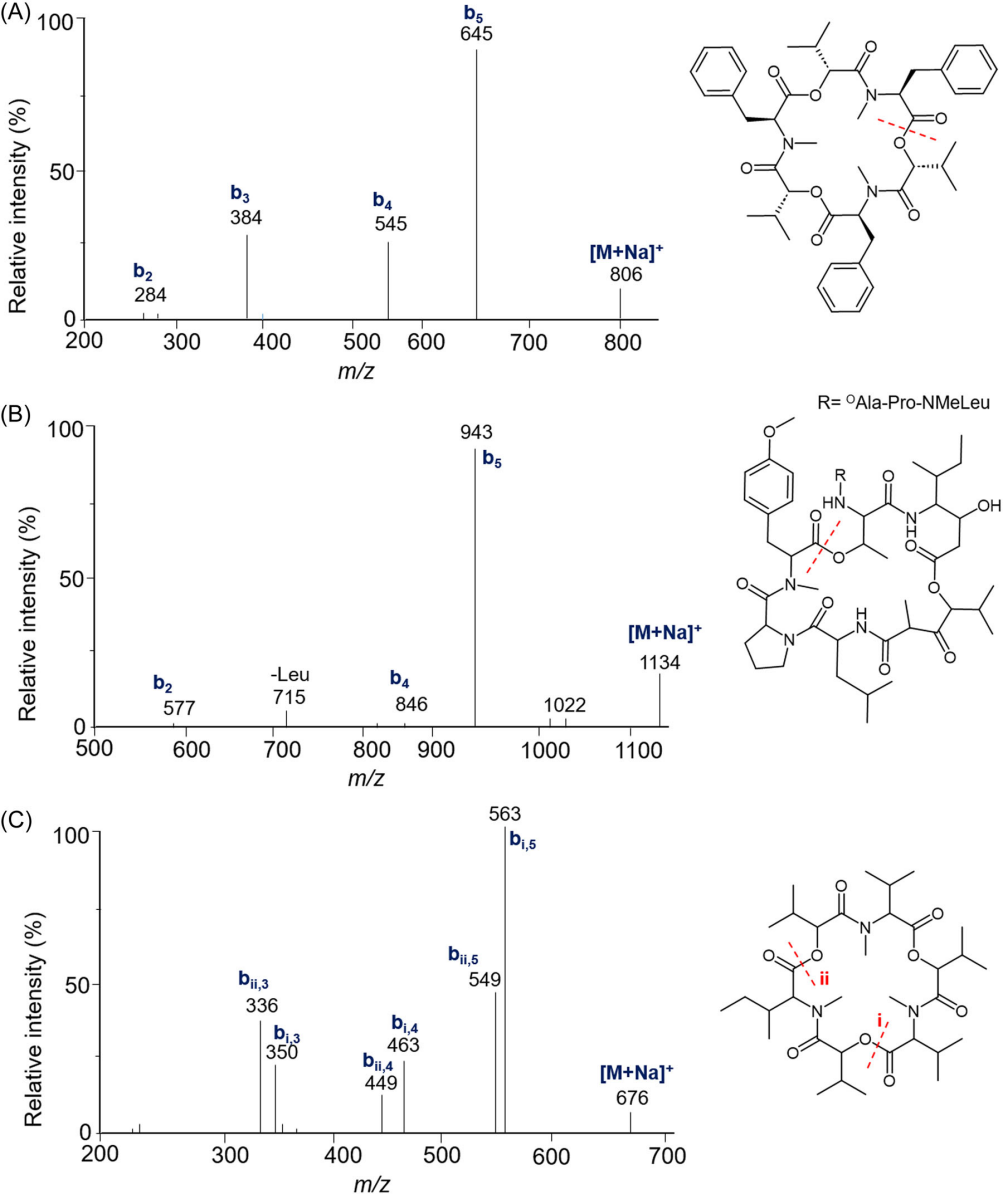
ESI product ion spectra of sodiated CDPs: (A) beauvericin (*m/z* 806), (B) didemnin B (*m/z* 1134), and (C) enniatin B1 (*m/z* 676) from ion trap (dotted lines for the ester linkage cleavage for ring opening). Figure adapted from Ngoka et al. (1999).

The sodiated CDPs are first naturally generated in the FAB source and now in the ESI source. A long time ago, the mechanism of their dissociations was well documented by Gross's team (Ngoka et al., 1999). They proposed, starting with beauvericin, didemnin B, and enniatin B1 as CDP antibiotics, a peptide sequence approach with reduced sequence overlaps from stepwise pathways. The first step of the process is based on sodium alkoxide salt formation resulting in specific ring‐opening of the [M + Na]^+^ precursor ion with an acylium at one end and at the other, the sodiated alkoxide (NaO‐CH) and non‐sodiated azanide (NaNH‐CH). This can be explained by the fact that the gas phase acidity of the hydroxy group end is higher than that of the primary or secondary amino group end. The resulting open CDPs yield exclusively the **b** series with the alkali retention on the product ions which are induced from the acylium end. At that time, the protonated oxazolone end (Polce et al., 2000; Yalcin et al., 1995) instead of the acylium end was not yet well introduced in the literature. Only more recently the evidence of the formation of protonated oxazolone (or eventually, diketopiperazine) intermediates in place of acylium has been used for the spectral interpretation of protonated CDPs (Jegorov et al., 2004). The protonated site of this 5‐membered intermediate, rather than the chiral CH site in the α‐position of the acylium end, provides the mobilizable proton needed to promote dissociation of the sodiated CDP and yields the **b** product ion series. Such an opened structure is called a *protonated salt* form (PS) (Colsch et al., 2017; Damont et al., 2019; Darii et al., 2017) in contrast to the *charge‐solvated* form (CS) where the ring remains intact and the cation is solvated with the lone pairs of electrons of heteroatoms of CDPs as it occurs with crown ethers (Armentrout, 1999; More et al., 1999) or with ionophores (Bauer et al., 2010; Cruz et al., 2007; Rose & Jenkins, 2007; Suwan et al., 1995) or non‐polar amino acids (Cerda & Wesdemiotis, 2000). The sodium alkoxide salt acts as a spectator of the process. When the ion/ion interaction is too weakened by its structural environment, the release of Na^+^ becomes possible (*vide infra*). In the first study of Nogoka et al., dissociation investigation of three sodiated CDPs (beauvericin, didemnin B, and enniatin B1) were done (Ngoka et al., 1999). From sodiated beauvericin, a covalent trimer (cyclo[^O^Val‐NMePhe]_3_, Figure 21A), after ring‐opening by an ester bond cleavage, abundant neutrals with *n* residues (1 ≤ *n* ≤ 3) and those weakly abundant with 4 residues, are competitively lost by cleavages of either the ester bond or of the NMe amide linkage.

The behavior of sodiated didemnin B (with peptidyl side chain with amide linkage to threonine) differs and its abundance is roughly 10% of the base peak (ion at *m/z* 943). This product ion corresponds to the ring‐opening at the ester bond between Thr and NMeTyrOMe, followed by the NMeTyrOMe loss. The abundance of other product ions is very low (Figure 21B). However, sequential MS^3^ experiment on the *m/z* 943 leads to abundant product ions due to competitive losses of residues such as: Pro (ion at *m/z* 846), [Pro+H_2_O] (*m/z* 828), [Pro+H_2_O+Leu] (*m/z* 715). The product ions due to loss of ^O^Val residue (*m/z* 577 ion) and water loss at (*m/z* 559) are also abundant. The loss of the α‐hydroxyl nonnatural amino acid residue (i.e., isostatine) remains a minor event in MS^4^. The water losses detected from each of the previous product ions are consecutive and originate from the free hydroxy group of the nonnatural amino acid residue. The latter example, the sodiated form of the enniantin B1, cyclo[NMeIle‐^O^Val‐(NMeVal‐^O^Val)_2_], dissociates first by competitive ring‐opening at the three ester linkages. Each of them results in overlapping **b** series product ions which explains the exclusive presence of doublets separated by 14 *m/z* (Figure 21C). This old study, albeit somewhat forgotten, is still relevant and should have the merit of blazing new trails for structural studies in the CDP field, as the collision spectra of these sodiated molecules are greatly simplified compared to those of protonated molecules. The importance of such an approach has only recently been reported by Li et al. in LC‐ESI‐MS/MS (Li, He, et al., 2020). The originality of their approach was to combine the product ion spectra of sodiated CDP molecular networks for data treatment. For this purpose, isolated CDPs from *Fusarium* sp. 17‐048 (i.e., enniatins A, A1, A2, B, B1, and B4) and from *Beauveria* sp. 186‐069 (e.g., beauvericin and analogues) were used. From such networks and low energy collision spectra in nonresonant mode, CDPs present in numerous contaminated food samples could be rapidly characterized. It is also worth noting that the structure of certain isomers of enniatins has been confirmed by NMR. Especially, ^13^C‐NMR has enabled us to differentiate NMe Leu from NMe Ile in the case of its congeners (i.e., A, A1, and A4). Despite this resurgence in the use of sodiated CDPs, these cationized forms are not more widely used.

Other differences must be mentioned, especially concerning the specific hydrogen‐bonded ion/dipole from plitidepsin A and B (*vide supra*, Figure 20A) as intermediate occurring through molecular isomerization before dissociation. This stepwise process is hindered by the alkali‐cationized plitidepsins and congeners, since such complementary ion pairs are not detected (Figure 20B). This behavior suggests that the approach of the mobile proton to the amide bond of the exocyclic chain linked to the terminal residue with salt will be prevented since this proton will preferentially neutralize negative charge of the neighboring alkoxide part of the salt, which do not occur from protonated plitidepsin A and B. Thus, this neutralization leads to the release of Na^+^ and the open CDP is therefore lost as a neutral (*vide infra*). As a result of cleavage in the neighboring linkage of the terminal sodium alkoxide salt (and the polar side chain), the mobilizable proton exothermically neutralizes the salt negative charge, thus releasing the Na^+^ cation. This prevents the formation of small size sodiated **b** fragment ions i.e., some of the sequence ions are missing from product ion spectra (Brandon et al., 2005). However, in such situation, the observed product ions resulting from dissociations of sodiated (or lithiated) CDPs and protonated CDPs yielding the **b** series are comparable with a *m/z* shift corresponding to the alkali metal cation mass minus the proton mass. Sodiated beauverolides [M+Na]^+^, the tetrapeptides [Leu/Ile‐*Hmoa*‐Phe‐Ala] with the ß‐hydroxy‐4‐methyl octanoic acid as *Hmoa*, mainly yield the direct water and Leu/Ile losses with sodium retention (ions [M+Na‐H_2_O]^+^, **b**_**3**_ and **a**_**3**_) after the ring‐opening through ester linkage cleavage between Leu/Ile and the *Hmoa* residue, resulting in the PS form using FTICR (Jegorov et al., 2004). Note that again, the lower size sodiated product ions are not detected although LMCO does not occur with this FTMS instrument. A similar **b**_***n***_ limit characterizes the dissociation of two minor enniatins (i.e., P1 and P2 with Thr as residue) from *Fusarium* sp. (Uhlig et al., 2009). Their respective sequence is: (i) cyclo[^O^Val‐**NMeVal**‐^O^Val‐NMe‐Thr‐^O^Val‐NMeVal] and (ii) cyclo[^O^Val‐**NMeLeu**‐^O^Val‐NMeThr‐^O^Val‐NMeVal], with modified residues in bold character. Under resonant excitation conditions using ion trap instrument, ring‐opening occurs competitively at the three ester linkages. The loss of water is important due to the presence of Thr residue from which the hydroxy group can be released by mobilizable proton catalysis. In addition, the exclusive loss of the C_3_H_8_ alkane from enniatin P1 and those of C_3_H_8_ and C_4_H_10_ from enniatin P2 reflect the presence of the NMeLeu residue in addition to NMeVal. Even more important is the size of the smallest product ion, corresponding to the **b**_**3**_ ion of each sequence resulting in ring‐opening at different ester bonds. This limit is above the *m/z* limit of the resonant mode. Using ion storage experiments, product ion spectrum of the sodiated (and lithiated) enniatin A, cyclo[NMeVal**‐\‐** ^O^Val‐(Ile‐^O^Val)_2_], displays the **b** series up to the **b**_**4**_ product ion (Williams & Brodbelt, 2004). The proposed mechanism of formation of the **b** and **y** series was based on the release of a neutral α‐lactams for each step of the sequential MS^n^ experiments. With other alkali‐cationized enniatin A, K^+^, and Rb^+^, no information is provided (Williams & Brodbelt, 2004). Note that the alkali‐cationized valinomycin dissociates in the same way to provide the **b** series until the **b**_**3**_ product ion (Williams & Brodbelt, 2004). Other metal cations were used, especially Pb^2+^ yielding singly charged enniatin A (by removing one proton) which dissociates by steps of double residues release (Williams & Brodbelt, 2004). Various leaded **b**_**1**_ product ions solvated with H_2_O molecule are generated from cationized enniatin A1. A similar behavior was observed from the leaded valinomycin which gives rise to the formation of **b** and **y** series up to the (**b**_**1**_ + H_2_O) and (**y**_**3**_ + H_2_O) product ions. This differs from leaded lipopeptide such as the iso‐surfactin which yields sequence until the **b**_**4**_ and **y**_**6**_ product ions (Williams & Brodbelt, 2004). In all previous studies performed in MS^n^, the alkali metal cations are retained on product ions of *n* ^th^ generation from the product ions of the (*n*−1)^th^ generation until those consisting in 2 or 3 residues and beyond, where the possible release is assumed without evidence.

Interestingly, the observed “product ion cut‐off” related to sodiated (or lithiated) product ions with two or three residues (depending on the residue size) must be enlightened. This was not attributed to LMCO of ion trap instrument since for other instruments such as ion beam Qq/TOF and triple quadrupole or Orbitrap‐based tandem combined with HCD cell, this residue number limit also characterizes the collisional spectra. A similar behavior characterizes the dissociations of various CDPs such as the enniatin and related species and beauvericin when they are studied (i) by MS/MS through nonresonant excitation in the collision cell of a Qq/TOF instrument (Li, He, et al., 2020) and (ii) by sequential MS^n^ experiments, in resonant excitation mode (Ngoka & Gross, 2000). In all cases, the lowest‐size sodiated product ions are made up of two residues.

This “product ion cut‐off” seems to be a general trend for the sodiated CDPs and a possible interpretation of this behavior will be explained and discussed below. In addition, the difficulties encountered in obtaining collision spectra of molecules cationized with K^+^ or Rb^+^ need to be explained.

**Figure 22:**
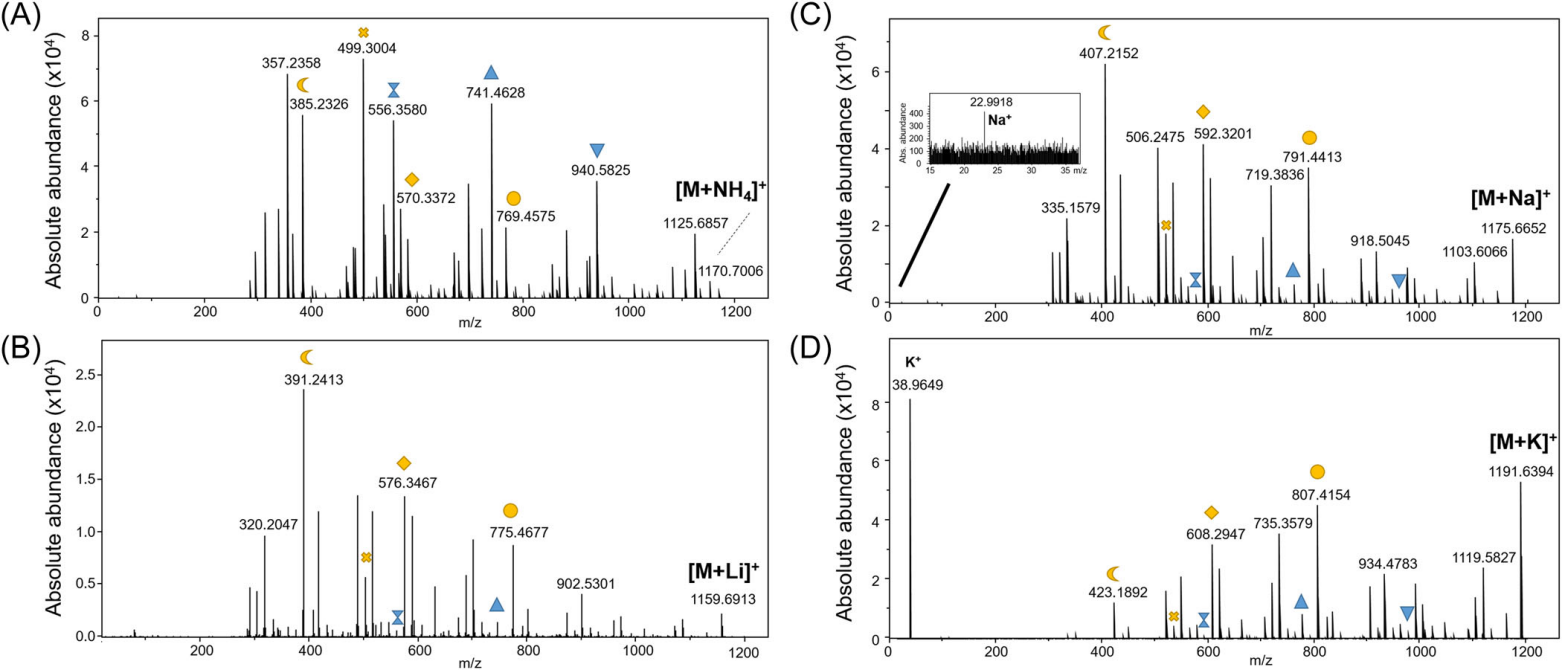
ESI product ion spectra of (A) [M + NH_4_]^+^, *m/z* 1170 (E_Lab_=60 eV), (B) [M + Li]^+^, *m/z* 1159 (E_Lab _= 94 eV), (C) [M + Na]^+^, *m/z* 1175 (E_Lab _= 94 eV), and (D) [M + K]^+^, *m/z* 1191 (E_Lab _= 94 eV) from Qq/TOF instrument. Each of the product ion series is annotated with a common label color in (B), (C), and (D) respectively shifted by 6.01 *m/z,* 21.98 *m/z,* and 37.96 *m/z* from those assigned to the same series displayed in (A). Figure adapted from Liuu et al. (2024).

Among the most studied CDPs models are valinomycin, cereulide, and its congeners. Cereulide, cyclo[Ala‐^O^Val‐Val‐^O^Leu]_3_, is analyzed mainly in its alkali‐cationized form. This choice of alkali metal cations was motivated by the fact that collision spectra were simplified, since only ester bonds were involved in ring‐opening (i.e., cyclo[Ala‐^O^Val‐Val**‐\‐** ^O^leu‐(Ala‐^O^Val‐Val‐^O^Leu)_2_] and cyclo[Ala**‐‐** ^O^Val‐Val‐^O^leu‐(Ala‐^O^Val‐Val‐^O^Leu)_2_]. This decreases the number of overlapping sequences (with different end residues) consisting only of product ions of the **b** series. As cereulide in solution is an ionophore of the potassium cation, potassiated cereulide was chosen for LC‐MS and MS/MS analyses for identification despite the limited sequence described for other potassiated CDPs using mainly ion storage instruments (Marxen, Stark, Frenzel, et al., 2015; Walser et al., 2022). A recent study on collision dissociations of cationized cereulide with alkali metals and ammonium agents was carried out to interpret their product ion spectra (Liuu et al., 2024). Both the resonant and nonresonant excitation modes were used to differentiate the processes taking place along competitive and consecutive pathways, respectively. To avoid ambiguity, the analysis of the product ions was carried out under high‐resolution conditions, using LIT/Orbitrap, Qq/Orbitrap, and Qq/TOF instruments. The use of the Qq/TOF instrument has the advantage of detecting product ions of low *m/z* values (i.e., lower than *m/z* 50) such as Na^+^ (*m/z* 23) or K^+^ (*m/z* 39), not available with the other instruments. However, for that, it is important not to use the default calibration range, but to extend it to include low *m/z* ratios. After this precaution, the product ion spectra can be recorded, revealing the presence of the alkali metal ions formed (Figure 22).

**Figure 23:**
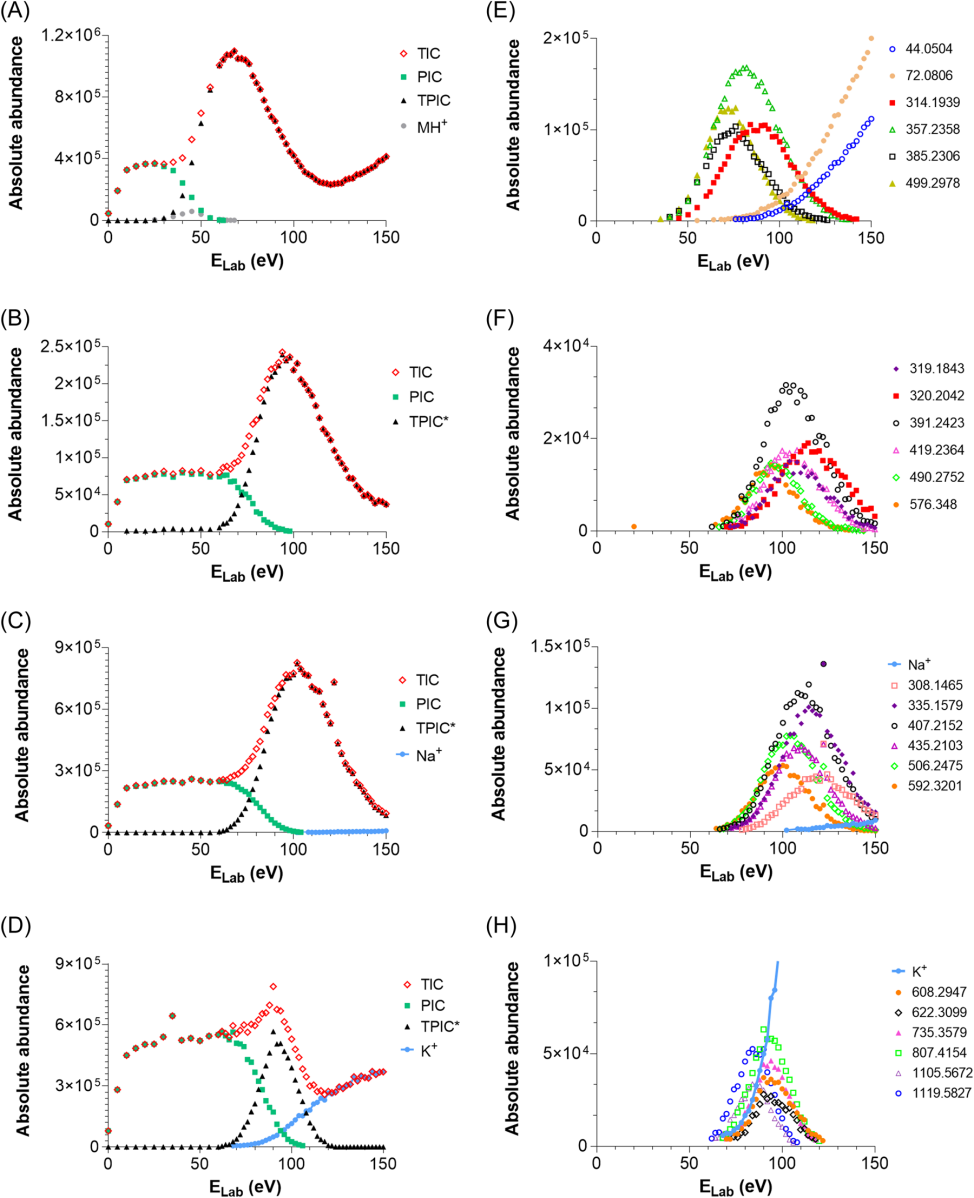
ERMS breakdown curves of the alkali‐cationized cereulide: (A, E) [M + NH_4_]^+^ (*m/z* 1170), (B, F) [M + Li]^+^ (*m/z* 1159), (C, G) [M + Na]^+^ (*m/z* 1175), and (D, H) [M + K]^+^ (*m/z* 1191). In (A–D), the profiles are based on the absolute abundances which correspond to (i) total ion current, TIC (◊), (ii) the total product ion current, TPIC or TPIC* with * without alkali metal ion abundance (▲), (iii) the survivor precursor ion current, PIC (￭) and (iv) the Na^+^ and K^+^ abundances (). In (e–h), only detailed profiles of main product ions, including Na^+^ and K^+^, are reported. In (h), to detail the main fragment ion profiles, the K^+^ absolute abundance was limited to 1 × 10^5^ a.u. Experiments were performed under nonresonant excitation conditions using Qq/TOF, with the *m/z* scale calibration adapted for K^+^ and Na^+^ detection and not for that of Li^+^. Figure adapted from Liuu et al. (2024).

It appears clearly that the product ions of alkali‐cationized species are all displayed into ammonium adduct spectrum with a mass shift corresponding to the retention of the alkali metal cation in each of the product ions promoted by mobilizable proton from the PS form. Furthermore, the collision spectra of [M + Li]^+^ and [M + Na]^+^ ions are almost superimposable, with the same low *m/z* limit, which is not the case for [M + K]^+^, which has a “product ion cut‐off” at a higher value. This behavior is consistent with the different studies of other CDPs, previously discussed. More to the point is the detection of alkali metal cation as base peak from dissociation of [M + K]^+^ (Bauer et al., 2010; Ducrest et al., 2019; Suwan et al., 1995). Despite this difference, identification continues to be based on the [M + K]^+^ dissociations (Walser et al., 2022). To shed further light on this effect and find its origin, the evolution of collision spectra as a function of collision energy (i.e., energy‐resolved MS, ERMS) was studied, with *y*‐axis abundance in absolute values instead of the usual relative values (Figure 23). Such ERMS breakdown curves allow to better demonstrate the instrumental effect on ion discrimination, that is, the effect on scattering of product ions of low *m/z* (a critical effect on alkali metal ion detection) at higher collision energy. These ERMS were compared according to the alkali metal cation and ammonium‐ionized cereulide.

**Figure 24:**
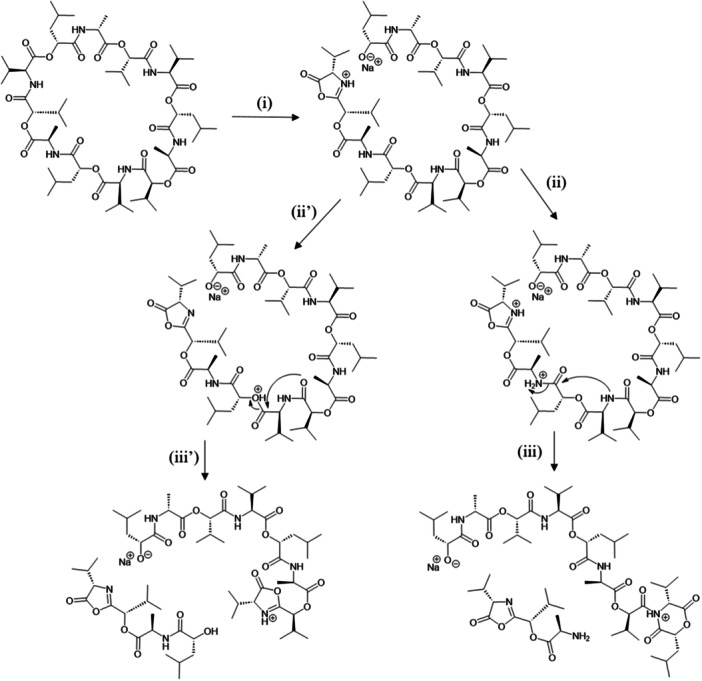
Proposed stepwise mechanims of the alkali‐cationized cereulide consisting in: (i) regioselective ring opening at ester linkages yielding the alkali‐cationized alkoxide salt at one end and at other a terminus oxazolone, followed by proton transfer either to (ii) amide linkage or (ii′) to ester linkage leading to formation of the **b** product ions in the form of either (iii) protonated diketo‐morpholine or (iii′) protonated oxazolone. Figure adapted from Liuu et al. (2024).

From these ERMS of [M + Li]^+^ and [M + Na]^+^ ions, the evolution of their respective total product ion current without alkali metal ion abundance, TPIC* (and TIC), appears very similar with comparable discrimination of the bare Li^+^ and Na^+^ cations and can be explained from the possible mechanisms as described in Figure 24. This behavior contrasts with that of [M + K]^+^, where the bare K^+^ cation appears at the same time as the first product ions (TPIC* in Figure 23) from the PS form. Furthermore, K^+^ becomes the lone ion at the highest collision energies, which in turn leads to the premature disappearance of the product ions thus explaining the “product ion cut‐off.” This clearly means that K^+^ formation comes from (i) small size product ions (within PS form) at the highest energies, and (ii) at the lowest energies, from the intact potassiated cereulide (within CS form). The latter is shown to be provided from dissociation of the CS form, that is, the closed form. In this study, the energy thresholds of Na^+^ and K^+^ are respectively comparable with that of the alkali‐cationized 12‐crown‐4‐ether (Armentrout, 1999) suggesting that these alkali metal cations are released from the CS forms at the highest energy for Na^+^, and at the lowest energy, for the K^+^ cation.

To conclude, data loss occurs from the potassiated cereulide and its use for cereulide identification should not be recommended, as in addition, sensitivity is lowered due to the role played by the CS form which contributes a lot to the [M + K]^+^ ion in contrast to what occurs with the Na^+^ and Li^+^ cations which could be the best precursor ions for CDP analysis purposes.

The exploration of the fragmentations of the CDPs should be developed by using other activation techniques such as EID (Cody & Freiser, 1979; Gord et al., 1993), and MAD (Berkout, 2006, 2009; Cook et al., 2009) for singly charged CDPs which give access to electronically excited ion and thus, could provide complementary structural information. Electron‐transfer‐dissociation can activate doubly and multiply charged positive species by neutralization of one charge. Closely this neutralization, a radical site emerges (i.e., OE ion formation in collision cell) which promotes competitive dissociations of the adjacent bonds. This dissociation occurring before any energy redistribution, the activtion is considered as non‐ergotic (Syka et al., 2004). The product ions are injected and then analyzed in linear ion trap analyzer. On the other hand, selected ion activation can occur in the FTICR cell. The precursor ions can be activated by other commercial excitation modes such as IRMPD (*vide supra*) for singly charged and multiply charged species. The doubly (or more) charged positive species can be also activated by electron‐capture dissociation (ECD) as applied for valinomycin and beauvericin (Cooper et al., 2004).

Now that a strategy based on high‐resolution MS/MS has been developed, particularly in the choice of molecular species to be activated by collision, it is necessary to describe how to obtain analyzable samples before MS analysis. In other words, it's about how to extract and prepare samples in such a way that they can be identified using methods based on the previous approaches and quantified using regulatory approaches.

## 4 DETECTION AND QUANTIFICATION OF CDPs BY MS

In parallel to structural characterization, MS detection and quantification of CDPs was implemented in the food safety and pharmaceutical sectors. Sample preparation before the separation and MS analysis is critical for method robustness and its ability to detect trace levels of analytes, notably for CDPs (Andel et al., 2017). This part focuses on the sample preparation protocols used for extraction of the CDPs for MS‐based trace analysis, either in food matrices (Table 6) or biofluids (Table 7). We excluded the analysis of CDPs in culture supernatants from producing bacteria, algae, fungus and some marine microorganisms, as well as the sample homogenization and test‐portion selection (few grams or a few dozen of microliters for biofluids).

**Table 6:** Summary of the principal steps of extraction protocols, analytical methods and performances for several CDPs in food.

Analytes	Matrix	Sample extraction	Column	Mobile phase composition	Mass analyser	LOQ	LOD	Range	Recovery	Intra day‐repetability	Inter‐ day precision	Reference
Cereulide	Bakery products	− Automatic solid extraction: 100°C, 10 kPa, methanol/pentane (1:1, v/v) − Evaporation and reconstitution in 1 mL of methanol	RP‐8	95% Acetonitrile + 4.9% water + 0.1% trifluoroacetic acid	Ion trap	50 ng/g	‐	50–500 ng/g	>70%	‐	<30%	Jääskeläinen et al. (2003)
Cereulide	Cooked rice, chinese noodle dish, french fries	− 50 mL of acetonitrile − Dilution with valinomycin (IS) and water	RP‐18	A: Water containing 2% trifluoroacetic acid and 10 mM ammonium acetate B: Acetonitrile	TQ	4 µg/kg	‐	4–100 µg/kg	97%–107%	2%–5%	2%–6%	Biesta‐Peters et al. (2010)
Cereulide	Boiled rice and boiled rice supplemented with 10% sunflower oil, milk pudding and liver sausage	− Addition of ^13^C_6_‐cereulide − Extraction with 10 mL ethanol − C18‐SPE, elution by ethanol (1 mL)	RP‐18	A: Water B: Methanol	Q/Trap	30–50 ng/g rice/rice with oil	3–5 ng/g rice/rice with oil	1–100 ng/mL in solvent	104%–111%	‐		Bauer et al. (2010)
Cereulide	Milk, cooked and composite food: rice‐based food, soybean‐based food, noodles, gratin	− Addition of 2–5 g of anhydrous sodium sulfate − Extraction twice with 20 mL of ethyl acetate − Evaporation and reconstitution in ethyl acetate/n‐hexane (1:9, v/v), − SPE on normal‐phase silica gel cartridge, elution by 10 mL of ethyl acetate/methanol (9:1, v‐v), − Evaporation and reconstitution in methanol	RP‐8	A: Water containing 1 mM ammonium formate and 0.1% formic acid B: Acetonitrile with 0.1% formic acid	TQ	0.5 ng/mL	0.1 ng/mL	0.0035−63 µg/g	67%–107%	‐	<10%	Yamaguchi et al. (2013)
Cereulide	Rice, pasta, and related meals	− Addition of ^13^C_6_‐cereulide − 15 mL of methanol − Dilution 1:1 in water	RP‐8	A: Water containing 1 mM ammonium formate and 0.05% formic acid B: Acetonitrile and 0.05% formic acid	TQ	1 ng/g	0,1 ng/g	1–500 ng/g	91‐93%	‐	3%–7%	Zuberovic Muratovic et al. (2014)
Cereulide	Rice, milk, and different ready‐to‐eat meals	− 1 mL of 75% ethanol ‐ MALDI matrix: α‐cyano‐4‐hydroxycinnamic acid	‐	‐	MALDI‐TOF		30 pg/mL	‐	‐	‐	‐	Ducrest et al. (2019)
Cereulide	Cooked rice, fried rice dish, cream pastry with chocolate, hotdog sausage, mini pancakes, vanilla custard and infant formula	Extraction based on ISO 18465 with addition of ^13^C_6_‐cereulide	RP‐18	A: Water containing 10 mM ammonium formate and 1% trifluoroacetic acid B: 100% acetonitrile	Depending of the 11 laboratories (TQ and Ion trap)	Below 1 μg/kg	‐	1–99 µk/kg	96.5% for mini pancakes to 99.3% for fried rice dish	Maximum 14%	Maximum 24%	in't Veld et al. (2019)
Isocereulides A–G	Boiled rice, food and feed samples (rice, puddings, desserts, cereals, baby foods, animal feeds) and two suspicious noodle samples	− Additon of ^13^C_6_‐cereulide − Extraction with 3 mL of acetonitrile	RP‐18	A: Water containing 10 mM ammonium formate and 0.1% formic acid B: Acetonitile/water (85:15, v/v) containing 0.25% formic acid	TQ	0.05 ng/g	0.1 ng/g	0,04–20 ng/mL in solvent	110%–114%	1%–3%	2%–8%	Marxen, Stark, Rütschle, et al. (2015)
Cereulide, beauvericin and enniatins A, A1, B and B1	Maize, wheat, pasta, and rice	− Extraction with acetonitrile/water (84:16 v/v) for all matrices except for rice 100% methanol − Evaporation and reconstitution in initial mobile phase composition	RP‐18	A: Water/acetonitrile/methanol (95:4:1, v/v/v) containing 1 mM ammonium formate and 0.3% formic acid, B: Acetonitrile/methanol/water (78:20:2, v/v/v) containing 1 mM ammonium formate and 0.3% formic acid	TQ	0.3–2.9 µg/kg	0.1–1.0 µg/kg	2–400 µg/kg	84%–106%	2%–20%	3%–20%	Decleer et al. (2016); (2019)
Beauvericin (BEA) and enniatins (ENNs) A, A1, B and B1	Oats, wheat and barley grains	80 mL of acetonitrile/water (84:16, v/v)	RP‐18	Water/methanol/acetonitrile (15:40:45, v/v/v) containing 15 mM ammonium formate	Ion trap	10–13 µg/kg	3.0–4 µg/kg	‐ BEA: 8–512 µg/kg ‐ ENNA 3–30 µg/kg ‐ ENNA1: 8–160 µg/kg ‐ ENNB: 8–152 µg/kg ‐ ENNB1: 14–432 µg/kg	‐ BEA: 115% ‐ ENNs: 80%–104%	‐	Mainly <25%	Uhlig and Ivanova (2004)
Beauvericin and enniatins A, A1, B and B1	Maize	160 mL of acetonitrle/water (84:16, v/v)	RP‐6	A: Water B: Acetonitrile	TQ	‐ BEA: 13 ng/g ‐ ENNs: 17–37 ng/g	‐	‐ BEA: 13–800 µg/kg ‐ ENNs 17– > 1000 µg/kg	‐ BEA: 96%–104% ‐ ENNs : 91%–110%	‐	8‐26%	Sørensen et al. (2008)
Beauvericin and enniatins A, A1, B and B1	Wheat grains and Maize	− 2 mL solvent mixture (acetonitrile/water/acetic acid, 79:20:1, v/v/v) − Dilution 1:2 same solvent	RP‐18	A: Methanol/water/acetic acid (10:89:1, v/v/v) containing 5 mM ammonium acetate B: Methanol/water/acetic acid (97:2:1, v/v/v) containing 5 mM ammonium acetate	Q/Trap	‐ BEA 3–4 µg/kg ‐ ENNs <1 µg/kg in maize (not validated in wheat)	‐	‐ LOQ to 48–864 µg/kg for ENNs ‐ LOQ to 800 µg/kg for BEA in maize ‐ LOQ to 80 µg/kg for BEA in wheat	94%–106%	‐	<2%	Sulyok et al. (2006); Suchowilska et al. (2010)
Enniatins and bassianolides	Maize, haylage, barley, and mixed silage	− Lyophilization − Extraction with (acetonitrile/water/acetic acid, 79:20:1, v/v/v) between 3 and 16 mL dilution 1:1 with the same solvent	RP‐8	A: Water containing 0.1% formic acid B: Acetonitrile containing 0.1% formic acid	Ion trap	‐	‐	‐	‐	‐	‐	Renaud et al. (2017)
Beauvericin and enniatins A, A1, B, and B1	Milk	− 10 mL of acidified acetonitrile (0.5% formic acid) − Addition of QuEChERS salts (8 g of MgSO_4_ and 1.2 g of NaCl) − Evaporation of 1 mL of supernantant and reconstitution in acetonitrile/water/acetic acid (49:50:1, v/v/v)	RP‐18	A: water containing 0.1% formic acid and 5 mM ammonium formate B: methanol	TQ	‐ BEA: 1.95 µg/L ‐ ENNs: 0.08–0.37 µg/L	‐ BEA: 0.59 µg/L ‐ ENNs: 0.02–0.11 µg/L	‐ BEA: 3.12–200 µg/L ‐ ENNs: 0.78–200 µg/L	‐ BEA: 91%–100% ‐ ENNs: 86%–120%	‐ BEA: 4% ‐ ENNs: max 5%	‐ BEA: 7% ‐ ENNs: max 8%	González‐Jartín et al. (2021)

Abbreviations: BEA, beauvericin; ENN, enniatin; IS, internal standard.

**Table 7:** Summary of the principal steps of extraction protocols, analytical methods and performances for several CDPs in biofluids.

Analytes	Matrix	Sample extraction	Column	Mobile phase composition	Mass analyser	LOQ	LOD	range	recovery	intra day‐repetability	inter‐ day precision	Reference
Enniatin B (ENNB) and Beauvericin (BEA)	Mice urine and serum	1.5 mL of acetonitrile	RP‐18	A: methanol/water/acetic acid (10:89:1, v/v/v) containing 5 mM ammonium acetate B: methanol/water/acetic acid (97/2/1,v/v/v) containing 5 mM ammonium acetate	Q/Trap	− BEA: 0.15 µg/kg urine and 0.10 µg/kg Serum − ENNB: 0.10 µg/kg urine and 0.05 µg/kg serum	‐	0.01–100 µg/L in solvent	BEA: 85‐90% ENNB: 88‐95%	<8%	<14%	Rodríguez‐Carrasco et al. (2016)
Cereulide	Porcine urine and plasma	− Ethanol, volume depending on weight 10–40 mL − Dilution 1:5 in water − C18‐SPE, elution with 2,5 mL of ethanol	RP‐18	A: water B: methanol	Q/Trap	‐	‐	‐	‐	‐	‐	Bauer et al. (2018)
Largazole thiol	human or rat plasma	− 1mL ethyl acetate − addition of harmine, as IS − Evaporation and reconstitution in 200 µL of methanol/water (60:40 v/v) acidified with 0.1% of formic acid	RP‐8	A: water containing 0.1% of formic acid B: methanol containing 0.1% of formic acid	TQ	12.5 ng/mL		12.5–400 ng/mL	101‐ 106%	7‐13%	7‐10%	Yu et al. (2014)
LY355703	Dog and Mouse Plasma	− Addition of LY354504 as IS − 200 µL of acetonitrile − Addtion of 1 mL of water − C18‐SPE, elution with 1 mL of acetronitrile − Evaporation of the supernatant and reconstitution in water/acetonitrile/propan‐2‐ol (50:45:5, v/v/v)	HP‐cyano	acetonitrile/water/propan‐2‐ol (63:30:7,v/v/v)	TQ	2 ng/mL	‐	2‐530 ng/mL	84‐107%	<9%	<8%	Berna et al. (1998)
Plitidepsin (Aplidin®)	Human plasma, whole blood, and urine	− Addition of didemnin B (IS) − 2 mL acetonitrile containing 1% formic acid. − 2 mL of chloroform − Evaporation of the supernatant and reconstitution in water/acetonitrile/formic acid (49.5:49.5:1, v/v/v)	RP‐18	A: water continaing 0.5% of formic acid B: acetronitrile containing 0.5% of formic acid	TQ	1 ng/mL	0,25 ng/	1‐200 or 250 ng/mL	46‐60%		<16%	Celli et al. (2004)
Plitidepsin (Aplidin®)	Human plasma, whole blood, and urine	− Extraction under basic condition (1 M aqueous ammonia) with TBME − Evaporation and reconstitution in acetonitrile/water (50:50, v/v) containing formic acid	RP‐18	A: water containing 0.1% formic acid and 5 mM ammonium acetate B: acetonitrile containing 0.1% formic acid	TQ	0.1 ng/mL	‐	0.1–100 ng/mL	>90%		<6%	Andel et al. (2017)
Romidepsin FK228	Human and mouse plasma	− 1.6 mL ethyl acetate − Addition of harmine (IS) − Evaporation of the supernatant and reconstitution in 50 µL of methanol/0.2% formic acid (55:45, v/v)	RP‐18	A: water containing 0.2% of formic acid B: methanol containing 0.2% of formic acid	Q	2 ng/mL		2–1000 ng/mL	Human plasma: 101.5% to 106.4%	0,6‐4%	0,6‐7%	Chen et al. (2008)
Valinomycin	Soybean, urine and plasma	− Soybean: 500 mL of water/ethanol (40:60, v/v) − Nanoparticles based microextraction	‐	‐	MALDI‐TOF		20–68 nM	0.2–1.0 M				Kailasa & Wu (2010)
Valinomycin	Human urine and plasma	− 2 µL of organic octanol − 1 µL of microdroplet contact at 240 rpm, 5 min − MALDI Matrix: α‐cyano‐4‐hydroxycinnamic acid	‐	‐	MALDI‐TOF		120 nM to 170 nM	0.5–2.5 mM				Wu et al. (2011)

Abbreviations: BEA, beauvericin; ENN, enniatin; IS, internal standard.

Because of the biological activities of CDPs, detection and/or quantification methods were developed to highlight the presence of toxic CDPs in diverse contaminated food samples or to determine the absorption/distribution/elimination profils in animals or humans after administration of CDP drug candidates. MS acquisition parameters for detection/quantification of CDPs were derived from the ionization conditions and fragmentation mechanisms already presented above (Section 3.2) and will not be developed further in this section. We illustrated rather the separation techniques and sample preparation protocols implemented to facilitate MS analysis of food and preclinical/clinical matrices. Such matrices contain a high diversity of small molecules, peptides, or proteins, all potentially responsible for deleterious matrix effects for MS‐based methods (Cortese et al., 2020).

Separation before MS, whatever the target matrix and the CDPs, were based primarily on reversed‐phase LC, to some exception (Berna et al., 1998): the chemistry of the stationary phase varied from C_6_ to C_18_, with various endcapping, depending on the manufacturer. Most of the separations were performed in gradient mode with mobile phase composition based on water/acetonitrile and/or methanol and other salts or acids adapted to each method to enhance the quality of the separation and of the ionization. Column's dimension, including particle size and the inherent flow rate were adapted to lower the run time to a few minutes in some studies (Decleer et al., 2016; 2019; Renaud et al., 2017), depending on the analytical system and the maximum acceptable pressure. After LC separation, most of the publications reported positive ionization with ESI, with some exception for APCI (Berna et al., 1998; Uhlig & Ivanova, 2004; Zöllner & Mayer‐Helm, 2006) whatever the MS or MS/MS system. Lastly, it is noticeable that some documented methods allowed quick direct analysis without LC separation using direct sample introduction *via* MALDI sources, both for food or biofluid samples (Ducrest et al., 2019; Wu et al., 2011).

### 4.1 Detection and quantification of CDPs in food samples

Some *Bacillus cereus* strains produce a small, highly heat‐resistant CDP toxin named cereulide. Cereulide is responsible for emetic syndrome in *Bacillus cereus* food‐borne outbreaks (Glasset et al., 2016; Rouzeau‐Szynalski et al., 2020). Several MS‐based methods have been reported to detect or quantify cereulide in food samples since 2002 (Häggblom et al., 2002). For solid‐liquid extraction, changes in the solvent extraction protocol were illustrated, using various solvents such as methanol (Zuberovic Muratovic et al., 2014), ethanol (Ducrest et al., 2019), acetonitrile (Biesta‐Peters et al., 2010), or ethyl acetate (Yamaguchi et al., 2013), as well as an increasing panel of food matrixes, including milk (Ducrest et al., 2019), rice (Ducrest et al., 2019), french fries or pasta (in't Veld et al. 2019) or some processed food, including baby food (Marxen, Stark, Rütschle, et al., 2015; in't Veld et al. 2019). Solid phase extraction (SPE) was also documented either on normal phase silica with ethylacetate/methanol elution (Yamaguchi et al., 2013) or on reversed C_18_ phase followed by elution with ethanol (Bauer et al., 2010). Note that accelerated solvent extraction was also previously reported with methanol/pentane (Jääskeläinen et al., 2003).

A notable improvement in the robustness of the methods was linked to the introduction of a synthetic stable‐isotope labeled ^13^C_6_‐cereulide in the protocol, instead of valinomycin, allowing stable isotope dilution analysis (SIDA), also called isotope dilution assays (IDA), for standardization and quantitative analyses (Bauer et al., 2010; Biesta‐Peters et al., 2010; Zuberovic Muratovic et al., 2014; in't Veld et al. 2019). The performance of the MS methods was evaluated during intra‐laboratory validation studies. For instance, Muratovic et al. reported precision between 3% to 7% and a limit of quantitation at 1 ng/g in rice and pasta (Zuberovic Muratovic et al., 2014). Interestingly, a study by Veld et al. in 2019 reported the inter‐laboratory evaluation of a method using acetonitrile extraction (in't Veld et al. 2019), ^13^C_6_‐cereulide as internal standard counterbalancing any matrix effects and multiple reaction monitoring (MRM) quantitation of cereulide in diverse samples (e.g., cooked rice, cream, sausage, pancakes, and infant formula). Results from the 11 participating laboratories showed quantitation level below 1 ng/g, excepted for one laboratory, good recoveries (from 96.5% to 98.3%), repeatability and reproducibility of the method with maximum CV at 14% and 24%, although different MS instruments were used. This method was standardized under the EN ISO 18465 reference.

The structural diversity of cereulide was recently considered. A new quantitative method was established for cereulide and seven isocereulide variants (A‐G). The method was validated in several food types: after acetonitrile extraction, quantification limits reached 0.5 ng/g with recoveries between 100% and 114% and good reported inter‐day precision. Isocereulides A to G were identified and quantified in two suspicious noodle samples, up to 268 ng/g, cereulide being predominant with 6198 ng/g (Marxen, Stark, Rütschle, et al., 2015).

Other CDPs are of particular concern in the food sector. Beauvericin, enniatins, destruxins and bassianolides belong to the family of mycotoxins and are produced by fungi. Similar extraction methods to cereulide were reported, using solvent extraction with water and acetonitrile acidified or not (Decleer et al., 2016; 2019; Sørensen et al., 2008; Suchowilska et al., 2010; Sulyok et al., 2006), or the more recent so‐called QuEChERS protocol (quick, easy, cheap, effective, rugged, and safe) (González‐Jartín et al., 2021; Koike et al., 2024), commonly used for pesticides analysis in food. With such protocol for multi‐toxins analysis in milk or fried rice, the extraction step with acetonitrile is followed by a liquid–liquid partitioning (demixion) resulting from the addition of inorganic salts. In this way, the polar components of the matrix remain in the aqueous layer leading to an extract suitable for MS analysis. For the toxins studied in milk, a second step of dispersive SPE clean‐up induced a reduction of recoveries due to adsorption on the C_18_ media and was thus abandoned.

### 4.2 Detection and quantification of CDPs in animal or human biofluids

Bioanalytical methods for clinical or preclinical studies require careful validation according to the regulations applicable when evaluating drug candidates (Moein et al., 2017). Accuracy and precision of the methods reported for CDPs were established with quality controls and met the requirements for bioanalytical assays, ranging between 85% and 115% and ± 15%, respectively.

The antitumor activity of selected CDPs (i.e., romidepsin FK228, plitidepsin commercialized under “Aplidin” name) led to pharmaceutical evaluation as anticancer drug candidates (Andel et al., 2017; Celli et al., 2004; Chen et al., 2008). After administration to animals or humans, measurements of circulating concentrations were required to determine the drug candidates' pharmacokinetic properties. To this aim, MS‐based methods were developed for the absolute quantification of CDPs in animal or human biofluids. After extraction of the CDPs, quantification was achieved mainly by reverse phase liquid‐chromatography (RP‐LC) coupled with single or MS/MS operated in MRM mode (Andel et al., 2017; Yu et al., 2014). Absolute quantification of the CDP levels was achieved by spiking a blank matrix with a standard solution of the CDPs and a stable isotopically labeled version, when available, used as an internal standard to compensate for the matrix effect (Andel et al., 2017).

Protocols for extracting CDPs from plasma or urine showed differences with the food safety sector. Usual approaches in bioanalytical laboratories were used, including precipitation/extraction of plasma/blood protein by organic solvents (Berna et al., 1998), liquid/liquid extraction (LLE) with organic solvent of different polarities: ethylacetate (Yu et al., 2014), methyl tert‐butyl ether (Chen et al., 2008), chloroform (Celli et al., 2004) or ethanol, eventually followed by SPE on a C_18_ cartridge (Bauer et al., 2018) or a combination thereof (Berna et al., 1998). For instance, van Angel et al. combined protein precipitation under basic condition and LLE for ptilidepsin quantitation in urine, human plasma, or whole blood down to 0.1 ng (Andel et al., 2017). Kailasa et al. reported an original protocol for valinomycin based on functionalized Ag_2_Se nanoparticles as effective extracting probes. A limit of detection (LOD) down to 20 nM was reached, and clean mass spectra were obtained by MALDI‐MS, even in plasma or urine samples (Kailasa & Wu, 2010). This antibiotic was also originally determined in urine and plasma by single drop microextraction followed by MALDI‐MS analysis reaching comparable detection limits (120 nM) (Wu et al., 2011).

Cereulide distribution was also studied in porcine urine and plasma, *via* ethanolic LLE followed by a C_18_‐SPE despite no method performance are described (Bauer et al., 2018). Regarding mycotoxin determination in biological samples, they were the subject of two reviews referring to LC‐API/MS or HRMS (Arroyo‐Manzanares et al., 2021; Zöllner & Mayer‐Helm, 2006). Interestingly, the distribution of enniatin B and beauvericin as potential anticancer drugs were investigated in mice urine and serum by acetonitrile extraction followed by MS analysis on a Q/Trap instrument with recoveries above 85% (Rodríguez‐Carrasco et al., 2016).

## 5 CONCLUSION AND OUTLOOK

Precise structural characterization of CDPs is essential for a comprehensive understanding of their biological and functional properties. NMR methods were used for a long time to obtain thorough structural characterization of CDPs, including insight into their stereochemistry and conformation, and were sometimes limited by their intrinsic low sensitivity. The limitations of these approaches were remedied by using MS‐based ones. Although GC‐EIMS methods proved relevant in obtaining insight into the nature and branching of the different building blocks constituting CDPs, ESI‐HRMS exhibited an unsurpassed ability to resolve their structural make up in a straightforward manner. Low energy fragmentation of adduct species (e.g., [M + Na]^+^, [M + Li]^+^ and less [M + K]^+^) of CDPs desorbed under ESI conditions also provides valuable and easy‐to‐interpret product ion spectra that allow CDP sequencing. Contrary to the expectations based on state‐of‐the‐art techniques mentioned in the introduction, the use of alkali‐cationized CDPs, and in particular the sodiated ones, makes the structural study of CDPs easier than the analysis of cyclopeptides. Indeed, unlike cationized cyclopeptides are essentially in *charged solvated* form, resulting in very little ring fragmentation, sodiated CDPs, mainly in *protonated salt* forms, allow mobile proton‐induced cleavage of the covalent bond after regioselective ring‐opening at the ester bond, to give the **b** series product ions.

Interestingly, some of these MS‐based methods were combined with efficient chromatographic separations and sample preparation protocols for quantitative applications in a diversity of matrices from the food safety and pharmaceutical sectors.

Although powerful, such ESI‐HRMS/MS strategies do not elucidate CDP's conformation and stereochemistry. In the future, strategies based on alternative fragmentation methods, such as those based on electron attachment or capture, could become effective additional tools for achieving this goal. Also, combining HRMS(/MS) with ion mobility spectrometry should facilitate separation of CDP (stereo)isomers and therefore holds great promise for gathering the different structural layers of information required to explore and better understand the huge chemical and functional diversity of naturally occurring CDPs.

## AUTHOR CONTRIBUTIONS

**Sophie Liuu**: Conceptualization; supervision; visualization; writing—original draft; writing—review and editing. **Annelaure Damont**: Writing—original draft; writing—review and editing. **Alain Perret**: Writing—original draft; writing—review and editing. **Olivier Firmesse**: Writing—original draft; writing—review and editing. **François Becher**: Writing—original draft; writing—review and editing. **Gwenaëlle Lavison‐Bompard**: Writing—original draft; writing—review and editing. **Amandine Hueber**: Writing—original draft; writing—review and editing. **Amina S Woods**: Writing—original draft; writing—review and editing. **Ekaterina Darii**: Writing—original draft; writing—review and editing. **François Fenaille**: Writing—original draft; writing—review and editing. **Jean‐Claude Tabet**: Conceptualization; writing—original draft; writing—review and editing.

## Footnotes

1 Wherever possible, the nomenclature used will follow the mass spectrometry recommendations of IUPAC (Murray et al., 2013).

2 Amino acids (His, Arg, Ser, and Thr) considered acid in the gas phase, the acidity as the enthalpy variation ΔH°_acid_(M) being related to the equation: M → [M‐H]^+^ + H^+^.

3 Comparable internal energy (with electronically excited levels) can be reached using EID (Cody & Freiser, 1979; Gord et al., 1993) which takes place from 10 eV electron interaction with selected ions. This mode should be developed in future for its instrument independent measurement. On the other hand, another fragmentation mode allows to provide formally similar internal energy and concerns low energy interaction with He* excited metastable atom beam. This mode is called as metastable atom‐activated dissociation (MAD) (Berkout, 2006; 2009; Cook et al., 2009).
